# DksA Controls the Response of the Lyme Disease Spirochete *Borrelia burgdorferi* to Starvation

**DOI:** 10.1128/JB.00582-18

**Published:** 2019-01-28

**Authors:** William K. Boyle, Ashley M. Groshong, Dan Drecktrah, Julie A. Boylan, Frank C. Gherardini, Jon S. Blevins, D. Scott Samuels, Travis J. Bourret

**Affiliations:** a Department of Medical Microbiology and Immunology, Creighton University, Omaha, Nebraska, USA; b Department of Medicine, UConn Health, Farmington, Connecticut, USA; c Division of Biological Sciences, University of Montana, Missoula, Montana, USA; d Laboratory of Bacteriology, Gene Regulation Section, Division of Intramural Research, Rocky Mountain Laboratories, National Institute of Allergy and Infectious Diseases, National Institutes of Health, Hamilton, Montana, USA; e Department of Microbiology and Immunology, University of Arkansas for Medical Sciences, Little Rock, Arkansas, USA; NCBI, NLM, National Institutes of Health

**Keywords:** *Borrelia burgdorferi*, DksA, Lyme disease, gene expression, global regulatory networks, stringent response

## Abstract

The pathogenic spirochete Borrelia burgdorferi senses and responds to changes in the environment, including changes in nutrient availability, throughout its enzootic cycle in Ixodes ticks and vertebrate hosts. This study examined the role of DnaK suppressor protein (DksA) in the transcriptional response of B. burgdorferi to starvation. Wild-type and *dksA* mutant B. burgdorferi strains were subjected to starvation by shifting cultures grown in rich complete medium, Barbour-Stoenner-Kelly II (BSK II) medium, to a defined mammalian tissue culture medium, RPMI 1640, for 6 h under microaerobic conditions (5% CO_2_, 3% O_2_). Microarray analyses of wild-type B. burgdorferi revealed that genes encoding flagellar components, ribosomal proteins, and DNA replication machinery were downregulated in response to starvation. DksA mediated transcriptomic responses to starvation in B. burgdorferi, as the *dksA*-deficient strain differentially expressed only 47 genes in response to starvation compared to the 500 genes differentially expressed in wild-type strains. Consistent with a role for DksA in the starvation response of B. burgdorferi, fewer CFU of *dksA* mutants were observed after prolonged starvation in RPMI 1640 medium than CFU of wild-type B. burgdorferi spirochetes. Transcriptomic analyses revealed a partial overlap between the DksA regulon and the regulon of Rel_Bbu_, the guanosine tetraphosphate and guanosine pentaphosphate [(p)ppGpp] synthetase that controls the stringent response; the DksA regulon also included many plasmid-borne genes. Additionally, the *dksA* mutant exhibited constitutively elevated (p)ppGpp levels compared to those of the wild-type strain, implying a regulatory relationship between DksA and (p)ppGpp. Together, these data indicate that DksA, along with (p)ppGpp, directs the stringent response to effect B. burgdorferi adaptation to its environment.

**IMPORTANCE** The Lyme disease bacterium Borrelia burgdorferi survives diverse environmental challenges as it cycles between its tick vectors and various vertebrate hosts. B. burgdorferi must withstand prolonged periods of starvation while it resides in unfed Ixodes ticks. In this study, the regulatory protein DksA is shown to play a pivotal role controlling the transcriptional responses of B. burgdorferi to starvation. The results suggest that DksA gene regulatory activity impacts B. burgdorferi metabolism, virulence gene expression, and the ability of this bacterium to complete its natural life cycle.

## Abstract (precis)

The Lyme disease bacterium Borrelia burgdorferi survives diverse environmental challenges as it cycles between its tick vectors and various vertebrate hosts. B. burgdorferi must withstand prolonged periods of starvation while it resides in unfed Ixodes ticks. In this study, the regulatory protein DksA is shown to play a pivotal role controlling the transcriptional responses of B. burgdorferi to starvation. The results suggest that DksA gene regulatory activity impacts B. burgdorferi metabolism, virulence gene expression, and the ability of this bacterium to complete its natural life cycle.

## INTRODUCTION

The pathogenic spirochete Borrelia burgdorferi transits through considerably different environments to complete its enzootic cycle (1–3). Ixodes ticks acquire B. burgdorferi during a blood meal from an infected mammalian host. Thereafter, B. burgdorferi persists in the tick midgut through the molt. A subset of midgut-localized B. burgdorferi spirochetes are transmitted to a vertebrate host when the next blood meal is acquired by the tick, which may occur up to 10 months after the initial acquisition (4, 5). As Ixodes ticks progress through their life stages, the dynamic milieu of the midgut presents B. burgdorferi with multiple challenges, including variations in osmolarity, pH, temperature, and nutrient availability, as well as oxidative and nitrosative stresses (3, 5–8).

B. burgdorferi spirochetes respond to changes in their environment through alterations in replication, metabolism, and outer surface protein expression (1, 3, 9–11). B. burgdorferi is a fastidious organism and an extreme amino acid auxotroph (12–14). The tick midgut following a molt and prior to a blood meal is a nutrient-limited and challenging growth environment for B. burgdorferi. Following a blood meal, nutrients are absorbed and sequestered from the tick midgut. B. burgdorferi responds by ceasing replication and upregulating genes required to utilize available carbon sources, glycerol and chitobiose (15–17). The expression of genes encoding tick-associated outer membrane proteins (*ospA* and *lp6.6*) and those for nitrosative and oxidative defenses (*napA, also known as dps* or *bicA*, and *uvrB*) is also required for transmission from the tick vector (3, 6, 9, 15, 18–22).

The stringent response contributes to the ability of bacteria to respond to environments with limited nutrients. Under starvation conditions, the stringent response directs resources from cellular replication through the repression of rRNA synthesis, while aligning resources to maintain protein synthesis by upregulation of aminoacyl-tRNA synthesis and glycolysis pathways. The hallmark of this response is the production of the signaling molecules guanosine pentaphosphate and guanosine tetraphosphate [(p)ppGpp] (23, 24). While the production of (p)ppGpp has many consequences for the bacterium, the primary outcome is a global shift in transcription by the interaction of (p)ppGpp with RNA polymerase (25, 26). The stringent response typically results in reduced DNA replication, translation, and fatty acid synthesis, as well as increased amino acid synthesis, glycolysis, and persistence-related gene expression, which together promote bacterial survival (24, 27–29). In B. burgdorferi, recent studies have shown that (p)ppGpp plays an important role in controlling the expression of genes required for survival within Ixodes scapularis (30–32).

A B. burgdorferi (p)ppGpp synthetase, Rel_Bbu_, is required for the global regulatory effects of (p)ppGpp (30, 31). Starvation of B. burgdorferi in the defined culture RPMI 1640 medium induces the stringent response and a measurable increase in (p)ppGpp production (30, 33). The transcriptomic response to cellular starvation provided insights into Rel_Bbu_-mediated regulation. The presence of (p)ppGpp increases the expression of genes that promote B. burgdorferi survival within Ixodes ticks, including glycerol and chitobiose utilization pathways, and *napA*. In addition, (p)ppGpp represses the expression of flagellar, DNA replication, and translation-related genes, suggesting that control of these genes under starvation conditions *in vivo* is due to the stringent response. Consistent with these phenotypes, Rel_Bbu_ functions in persistence in ticks and transmission from infected nymphs to mice (30).

The B. burgdorferi stringent response, mediated through (p)ppGpp, plays a key role in survival within I. scapularis (30); however, the role of DnaK suppressor protein (DksA) has not been investigated. DksA has emerged as an important accessory regulator of the stringent response in other bacteria (34). In Escherichia coli, DksA is specifically required for upregulation of amino acid biosynthesis, tRNA synthesis, and cellular utilization of alternative sigma factors (such as RpoS) that integrate the stringent response (28, 35–39). In addition, DksA holds a key regulatory role in the life cycle of several bacterial pathogens and is implicated in virulence gene expression (34, 40–42). In enterohemorrhagic E. coli, DksA-dependent regulation is required for the enterocyte effacement response during intestinal colonization (43, 44). In Pseudomonas aeruginosa, the stringent response mediates colonization of surfaces by biofilm formation (45). Salmonella enterica requires the stringent response to respond to acidic, oxidative, and nutrient-limited environments within macrophages (46, 47). In these cases, DksA works synergistically with the stringent response and is indispensable for adaptation. As seen in other bacteria, B. burgdorferi responds to starvation by the production of (p)ppGpp (30, 33), but the contribution of DksA to the regulation of the stringent in the spirochete response is unknown.

In this study, we expand the understanding of the B. burgdorferi stringent response by characterizing the role of a DksA ortholog during adaptation to nutrient limitation. We generated a *dksA* mutant strain of B. burgdorferi and starved the spirochetes in RPMI 1640 medium to evaluate the role of DksA during the stringent response. Compared to Barbour-Stoenner-Kelly II (BSK II) medium, RPMI 1640 medium lacks numerous nutrients required for the growth of B. burgdorferi, including fatty acids, oligopeptides, and *N*-acetylglucosamine, along with a lower concentration of glucose (48–50). During starvation in RPMI 1640 medium, B. burgdorferi ceases replication and increases the synthesis of (p)ppGpp (30, 33). A whole-transcriptome analysis using the widely used custom B. burgdorferi Affymetrix microarray chip (51–54) was used to examine the responses of wild-type and *dksA* mutant spirochetes to starvation. The following results indicate that starvation of B. burgdorferi in RPMI 1640 medium led to a DksA-dependent shift of the global transcriptome and support the designation of the *bb0168* gene product as a functional DksA.

## RESULTS

### Characterization of a putative DksA encoded by *bb0168*.

**FIG 1:**
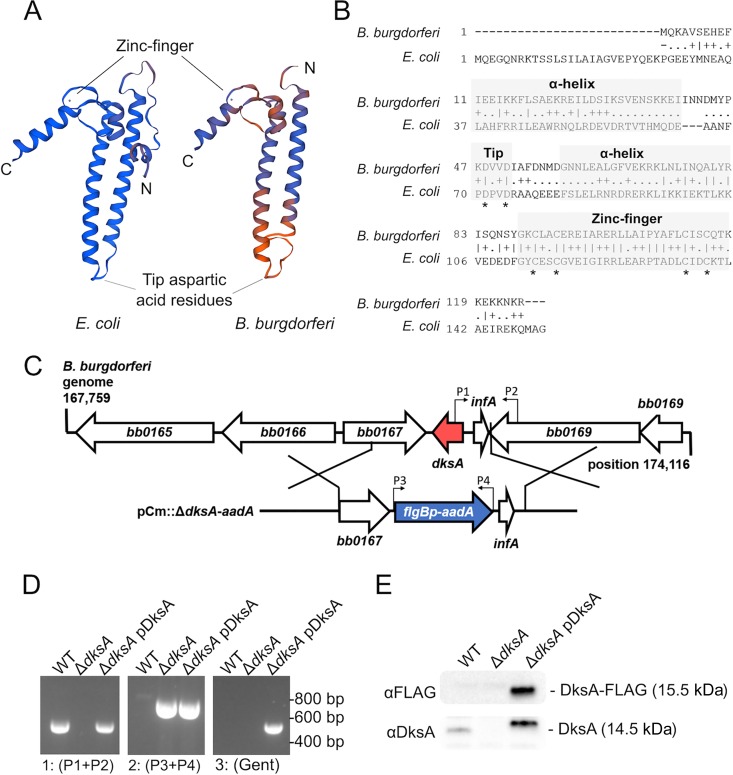
Amino acid sequence analysis and mutagenesis of conserved *B. burgdorferi bb0168*-encoded DksA. (A) SWISS-MODEL of *E. coli* and *B. burgdorferi* DksA proteins illustrate predicted structural similarities based on a high-resolution crystal structure (PDB 1TJL). The color scale from blue (high) to orange (low) indicates score estimating model quality. Peptide N and C termini are indicated for each model. (B) Amino acid sequence alignment of *B. burgdorferi* and *E. coli* DksA proteins. The boxes indicate regions where *B. burgdorferi* DksA likely contains conserved coiled-coil α-helices and a zinc finger based on HHPred homology modeling. The asterisks indicate key conserved aspartic acid and cysteine residues. (C) A schematic of the *bb0168* (*dksA*) genomic location and homologous recombination mutagenesis strategy. The open reading frame identity and direction are indicated by large arrows, and the positions of the primers used in panel D are indicated by small arrows above the genes. (D) Homologous recombination between the *B. burgdorferi* genome and the plasmid encoding the 600-bp segment containing *dksA*-flanking regions and the *aadA* antibiotic resistance cassette (blue) was confirmed by PCR. The Δ*dksA* mutant strain no longer possesses the *dksA* sequence (red) as detected by PCR using the primers P1 and P2, and it contains the *aadA* gene, as detected with primers P3 and P4. Additionally, the Δ*dksA* strain was *trans* complemented with the pBSV2G-based pDksA plasmid and confirmed by the presence of *dksA* detected by PCR using the primers P1 and P2, *aadA* gene detected with the primers P3 and P4, and gentamicin (Gent) resistance gene (*aacC1*) with the primers *aacC1* F/R primers. (E) Complementation was further confirmed by Western blotting using antibodies targeting the FLAG (top) and DksA (bottom) epitopes.

DksA homologs are encoded in many bacterial genera, including Borrelia species. The structure of DksA has been extensively characterized in E. coli (55, 56). Protein interaction studies have demonstrated that the E. coli DksA protein’s α-helices in the coiled-coil motif interact with the RNA polymerase secondary channel, and that the coiled coil-tip aspartic acid residues exert DksA function in the RNA polymerase core (57–59). In addition, DksA harbors a zinc finger domain that potentially modulates its protein function (60, 61). A SWISS-MODEL was generated for the 125-amino-acid DksA protein encoded by the B. burgdorferi *bb0168* open reading frame (ORF) (GenBank accession no. AAC66562) using an E. coli DksA crystal structure (PDB 1TJL) as the template (57), and the model was visualized alongside the 150-amino-acid E. coli DksA protein for comparison (Fig. 1A). The B. burgdorferi DksA harbors an N-terminal 31-amino-acid truncation and is nearly 3 kDa smaller than the E. coli DksA, at 14.5 kDa compared to 17.5 kDa. The B. burgdorferi DksA model also predicts three additional amino acids in an unstructured region between the C-terminal end of the first α-helix and coil tip compared to the DksA crystal structure (PDB 1TJL). The B. burgdorferi DksA has only 23.6% amino acid sequence identity to E. coli DksA; however, the SWISS-MODEL local quality estimate indicates high similarity within the coiled-coil motif and the C-terminal region (0.6 to 0.9 quality score). Moreover, an alignment of the E. coli and B. burgdorferi primary DksA amino acid sequences using the HHPred (62) algorithm predicts conservation of key amino acids in DksA, including the coiled-coil-tip aspartic acids in the α-helices, and the cysteines forming the zinc finger motif (Fig. 1B). Alignment of the amino acid sequences of DksA among *Borrelia* species with Clustal Omega (63) indicates high amino acid sequence identity within the *Borrelia* genus (see Fig. S1 in the supplemental material).

### Generation of B. burgdorferi Δ*dksA* mutant strain and *trans*-complemented Δ*dksA* pDksA mutant strain.

To study the role of *dksA* in the B. burgdorferi stringent response, a *dksA* mutant of B. burgdorferi (Δ*dksA*) was generated in the B31-A3 background. The entire *dksA* (*bb0168*) ORF was replaced by homologous recombination with a B. burgdorferi *flgB* promoter-driven streptomycin resistance cassette (*flgBp-aadA*) used for selection (Fig. 1C). The *dksA* mutant strain (Δ*dksA*) was complemented in *trans* with the shuttle vector pBSV2G (64) containing a *dksA* ORF fused to a sequence encoding a C-terminal FLAG epitope tag along with 600 bp of *dksA* upstream sequence (pBSV2G::*dksA*-FLAG, pDksA). The presence of the chromosomal copy of the *dksA* gene was determined by PCR (Fig. 1D). The expression of DksA_FLAG_ protein in the Δ*dksA* pDksA mutant strain was confirmed by Western blotting using antibodies against FLAG and DksA epitopes (Fig. 1E).

### Adaptation of the Δ*dksA* and Δ*rel*_Bbu_ mutants to prolonged starvation.

**FIG 2:**
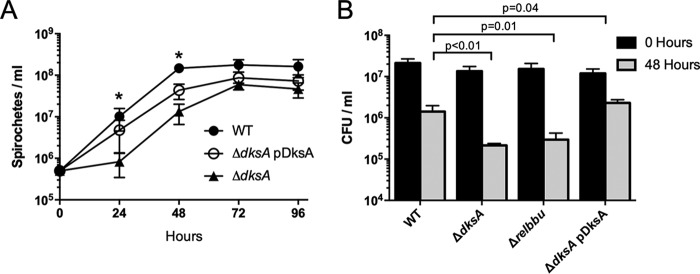
Evaluation of *B. burgdorferi* growth rate and survival during long-term starvation. (A) Growth of wild-type (WT), Δ*dksA* mutant, and Δ*dksA* pDksA mutant (passaged at 5 × 10^5^ spirochetes ml^−1^) in BSK II medium was assayed by enumeration at 24-h intervals. Data points represent the mean of the values from four biological replicates. Error bars represent standard deviations, and asterisks indicate *P* values of <0.05 by one-way ANOVA. (B) Wild-type (WT), Δ*dksA* mutant, Δ*rel*_Bbu_ mutant, and Δ*dksA* pDksA mutant cultures grown to 5 × 10^7^ spirochetes ml^−1^ density in BSK II medium were pelleted and resuspended in RPMI 1640 medium for 0 or 48 h prior to growth in semisolid BSK II medium. Data represent the means of the results from three independent experiments. The *P* values were calculated by ANOVA with a Dunnett’s multiple comparison for spirochete density following 48 h of starvation in RPMI 1640 medium.

B. burgdorferi wild-type and Δ*dksA* mutant strains are morphologically similar during logarithmic growth in BSK II medium under microaerobic conditions (5% CO_2_ and 3% O_2_). Wild-type, Δ*dksA* mutant, and Δ*dksA* pDksA mutant spirochetes maintain similar maximal growth rates during logarithmic growth (Fig. 2A). The Δ*dksA* mutant exhibited a prolonged lag phase and lower cell densities at stationary phase than both wild-type and Δ*dksA* pDksA mutant strains when passaged at equivalent densities (*P* < 0.05). When cultures were inoculated at a low density of 1 × 10^5^ spirochetes ml^−1^, the Δ*dksA* mutants exhibited elongated morphology compared to the wild type at early time points and at stationary phase (Fig. S2). The relative lengths of spirochetes were measured using ImageJ (65), and the Δ*dksA* mutant was significantly longer than the wild type (*P* = 0.004) at stationary phase (Fig. S2).

To determine if DksA affects survival during nutrient stress, wild-type, Δ*dksA* mutant, Δ*rel*_Bbu_ mutant, and Δ*dksA* pDksA mutant spirochetes were cultured to 5 × 10^7^ spirochetes ml^−1^ and then starved in RPMI 1640 medium for 0 or 48 h, and the number of CFUs was determined by plating cells in semisolid BSK II medium. A recent study demonstrated that a *rel*_Bbu_ mutant B. burgdorferi (Δ*rel*_Bbu_) exhibited a defect in adapting to starvation in serum-free RPMI 1640 medium (30). We generated a Δ*rel*_Bbu_ mutant strain in the B31-A3 background, as described previously (30), and, consistent with previous results, Δ*rel*_Bbu_ mutant cultures yielded significantly lower numbers of CFU following 48 h of starvation in RPMI 1640 medium compared to wild-type cultures (Fig. 2B). Following prolonged starvation, Δ*dksA* mutant cultures exhibited a reduction in CFU similar to Δ*rel*_Bbu_ mutant cultures. The Δ*dksA* pDksA mutant restored CFU to wild-type levels following starvation, suggesting that DksA functions in the adaptation of B. burgdorferi to starvation.

### Global transcriptome of the *dksA* mutant during logarithmic-phase growth.

**FIG 3:**
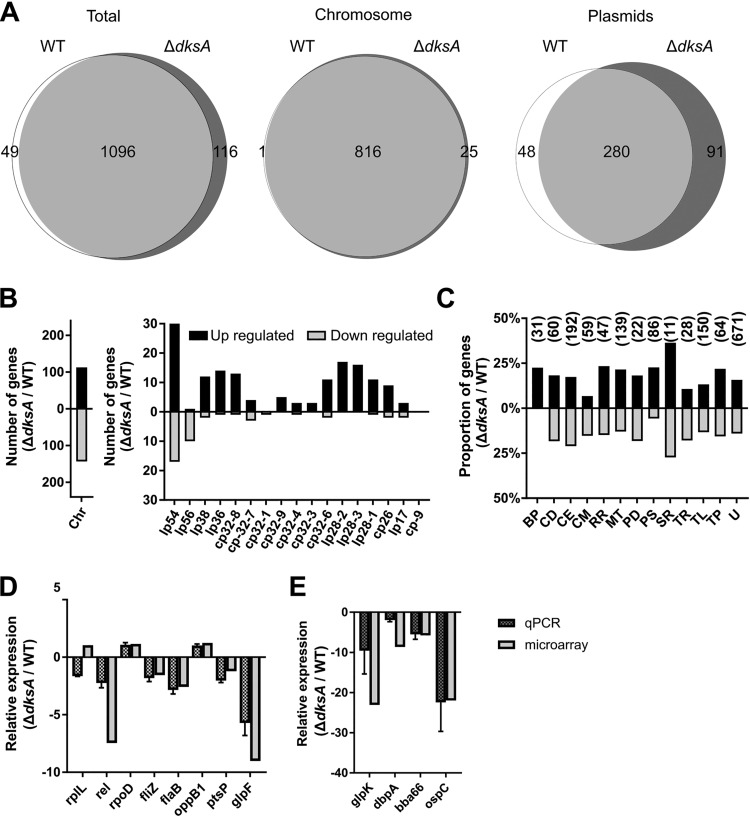
Relative RNA expression between wild-type (WT) and Δ*dksA* mutant during logarithmic-phase growth. (A) Venn diagrams illustrate the total number of genes expressed by the WT and Δ*dksA* mutant during mid-logarithmic phase. Expression of individual genes was determined by detection of a microarray hybridization signal above background among three biological and three intrachip hybridization replicates (left). Genes expressed by both the WT and Δ*dksA* mutant are represented in the intersection of the two circles, and the genomic location (chromosome or plasmid) is indicated (middle and right). (B) The number of genes upregulated (higher levels in Δ*dksA* mutant than in WT) or downregulated greater than 2-fold and their genomic location of chromosome (Chr), linear (lp), or circular (cp) plasmids. Only comparisons with FDR-adjusted *P* value of <0.05 are shown. (C) Differentially expressed genes were functionally categorized with the following abbreviations: BP, bacteriophage; CD, cell division; CE, cell envelope; CM, chemotaxis and motility; RR, DNA replication and repair; MT, metabolism; PD, protein degradation; PS, pseudogene; SR, stress response; TR, transcription; TL, translation; TP, transporter proteins; and U, unknown. The bars indicate percentages of genes upregulated and downregulated relative to the total number of genes of each category, and numbers above the bars indicate total numbers of genes within the respective functional group. (D and E) The differential regulation of select genes with high microarray signal quality or genes implicated in stringent response and infectivity were confirmed by RT-qPCR. Differential expression data by RT-qPCR and microarray are presented side by side and organized by function: ribosome (*rplL*), stringent response (*rel*_Bbu_), transcription (*rpoD* and *fliZ*), motility (*flaB*), transport (*bb0332* and *glpF*), metabolism (*ptsP* and *glpK*), and lipoproteins (*dbpA*, *bba66*, and *ospC*). Data represent the mean of four biological replicates, and error bars indicate standard deviations.

To investigate DksA-dependent transcription during growth in nutrient-rich medium, RNA was harvested from wild-type and Δ*dksA* mutant cultures and analyzed by microarray. For these comparisons, genes were considered expressed if the hybridization signal for an ORF was significantly above background (Fig. 3A). To evaluate differential expression, we constrained the reporting of genes to only the genes differentially expressed by 2-fold (linear scale) or more and disregarded genes whose average hybridization signals were below background levels or when microarray false-discovery rate (FDR)-adjusted *P* values were 0.05 or more. The differentially regulated genes were then categorized by genomic location (chromosome or plasmid) (Fig. 3B) and function based on gene ontology (Fig. 3C) to gain insights into DksA-dependent gene expression during logarithmic-phase growth.

During mid-logarithmic-phase growth, the Δ*dksA* mutant exhibited an altered transcriptional profile compared to wild-type spirochetes, suggesting that DksA is important for gene regulation during growth. The Δ*dksA* mutant expressed 1,212 genes compared to 1,145 genes in the wild type (Fig. 3A) located across the chromosome and numerous circular and linear plasmids (Fig. 3A and B). The differential regulation analysis revealed that 268 genes were more highly expressed in the Δ*dksA* mutant than in the wild-type strain (Table S1), while 186 transcripts were expressed at lower levels by the Δ*dksA* mutant (Table S2). Because both Δ*dksA* and Δ*rel*_Bbu_ mutants are susceptible to starvation in RPMI 1640 medium, we assessed the overlap of the putative DksA and Rel_Bbu_ regulons by matching genes similarly regulated by either the Δ*dksA* or Δ*rel*_Bbu_ mutant. Overlap with two previous transcriptomic studies identifying genes differentially regulated in the Δ*rel*_Bbu_ mutant indicate that up to 115 genes are cooperatively regulated by DksA and Rel_Bbu_ (Tables S1 and S2). The genes encoding glycerol utilization proteins, *glpF* and *glpK*, and oligopeptide transporters, *oppA1* and *oppA2*, were similarly downregulated in the Δ*dksA* and Δ*rel*_Bbu_ mutants compared to the wild type. The expression of genes encoding tick-associated outer membrane proteins, *ospA* and *lp6.6*, and the antioxidant defense gene *napA* was also similarly regulated in the Δ*rel*_Bbu_ mutant strain. The Δ*dksA* mutant additionally expressed genes associated with stress responses at higher levels than the wild-type strain (Table S1), including those encoding chaperones (*dnaK* and *dnaJ*), those encoding DNA repair proteins (*ligA* and *uvrB*), and numerous bacteriophage genes (*bbl01* and *bbn23*). In addition, the Δ*dksA* and Δ*rel*_Bbu_ mutants both exhibit increased expression of selected genes encoding ribosomal proteins (*rpmA*, *rplB, rplV, rpsS*, and *rpsC*), suggesting that both (p)ppGpp and DksA are required to suppress these genes. These results suggest that Rel_Bbu_ and DksA regulons partially overlap.

To validate the microarray findings, quantitative real-time PCR (RT-qPCR) was performed comparing wild-type and Δ*dksA* mutant spirochetes during logarithmic-phase growth (Fig. 3D and E). RT-qPCR confirmed the relative expression of genes that produced high microarray signal quality (*oppB1* and *ptsP*), are implicated in the stringent response (*rel*_Bbu_, *glpF*, and *glpK*), have housekeeping functions (*rplL*, *rpoD*, and *flaB*), or are required for infectivity (*dbpA*, *bba66*, and *ospC*). Many of these genes (*rplL*, *rpoD*, *fliZ*, *flaB*, *bb0332*, and *ptsP*) are highly expressed genes, with nearly 100 transcripts per 1,000 transcripts of 16S rRNA during logarithmic-phase growth. Transcriptional studies have indicated that the glycerol utilization pathway is a key metabolic pathway regulated by the stringent response (30, 31). Two genes, *glpF* and *glpK*, encoding the glycerol transporter and kinase, respectively, were expressed at lower levels in the Δ*dksA* mutant than in the wild type, indicating an overlap in the regulation of the glycerol utilization pathway (Table S2). Eleven of the 12 genes assayed exhibited the same direction and similar magnitude of relative expression in the RT-qPCR and microarray results. These data corroborate the findings of our microarray experiments and indicate a global effect of DksA on transcription.

### DksA mediates transcriptional responses to starvation.

**FIG 4:**
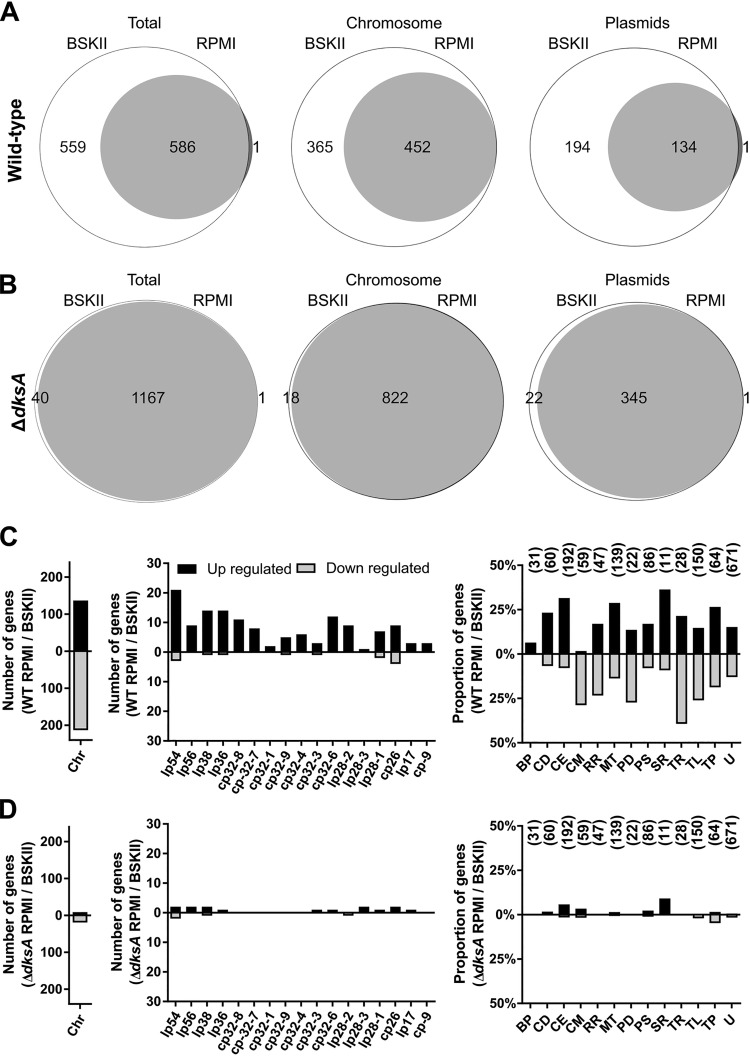
DksA mediates transcriptional responses to starvation. (A and B) Venn diagram illustrates the number of genes expressed during mid-logarithmic-growth phase (BSK II medium) or during starvation (RPMI 1640 medium) by wild-type (WT) *B. burgdorferi* (A) or by the Δ*dksA* mutant strain (B). The data are represented as the total number of genes (left) or divided into number of chromosomal (Chr) or plasmid-carried genes. Genes expressed exclusively during mid-log phase or during starvation are represented outside the union of the two circles, whereas the genes expressed in both are represented within. (C and D) The number of differentially expressed genes by cultures of WT (C) and Δ*dksA* mutant (D) strains during starvation (RPMI 1640 medium) compared to mid-logarithmic-phase cultures (BSK II medium). Bars represent the number of genes differentially expressed on the chromosome (Chr), on the various plasmids, or the percentage of genes differentially expressed within the annotated functional categories relative to genes within the respective functional groups. The bars indicating proportions in the following categories: BP, bacteriophage; CD, cell division; CE, cell envelope; CM, chemotaxis and motility; RR, DNA replication and repair; MT, metabolism; PD, protein degradation; PS, pseudogene; SR, stress response; TR, transcription; TL, translation; TP, transporter proteins; and U, unknown. Numbers above the bars indicate the total number of genes within respective functional groups. Genes were considered differentially expressed if comparisons with FDR-adjusted *P* value of <0.05 and differential expression of 2-fold or more.

DksA orthologs regulate transcription in model bacteria (39, 56, 66–70). Therefore, we evaluated the role of DksA in the B. burgdorferi stringent response by comparing differences of the transcriptional responses of wild-type and Δ*dksA* mutant strains to starvation in RPMI 1640 medium. For microarray analysis, RNA was harvested from cultures grown to mid-logarithmic-growth phase followed by 6 h of incubation in serum-free RPMI 1640 medium. In wild-type spirochetes undergoing starvation, there was a dramatic reduction in the number of genes exhibiting above-background microarray hybridization signals. While 1,145 genes were expressed in wild-type spirochetes during logarithmic growth in BSK II medium, only 587 genes were detected in wild-type spirochetes following starvation in RPMI 1640 medium, revealing a global reduction in transcription (Fig. 4A). A total of 274 genes were upregulated and 226 genes downregulated in response to starvation, indicating a restructuring of the wild-type transcriptome (Table S3), consistent with previous results obtained using differential RNA sequencing analysis (30). In contrast, the Δ*dksA* mutant undergoing starvation retained expression of the majority of genes expressed during logarithmic growth in BSK II medium (Fig. 4B). Within this sizable subset of genes expressed in the Δ*dksA* mutant, only 47 genes were differentially regulated (Table S4). Thus, transcriptional remodeling of the genome during nutrient stress is to a great extent dependent on DksA.

Genes differentially expressed by wild-type spirochetes undergoing starvation were organized by gene location and functional category to characterize the transcriptional response. In wild-type spirochetes, transcriptional downregulation in response to starvation is mostly limited to chromosomally carried genes (Fig. 4C). Two hundred thirteen of the total 226 downregulated genes were on the chromosome. Downregulated chromosomal genes are overrepresented in the following four functional categories: chemotaxis and motility, DNA replication and repair, transcription (and transcriptional regulation), and translation. Among the chemotaxis and motility genes, 13 of the 17 downregulated genes encoded flagellar components (Table S3). Genes encoding DNA replication proteins were also downregulated, including *gyrA* and *gyrB* (3.4- and 2.4-fold lower, respectively), encoding DNA gyrase; *dnaB* (3.2-fold lower), encoding the replicative DNA helicase; and *dnaN* (5.2-fold lower), encoding the β-clamp of DNA polymerase. The expression of DNA replication and flagellar synthesis genes is required for cell division, and B. burgdorferi CFU do not increase during starvation in RPMI 1640 medium. Additionally, we identified 39 downregulated genes encoding translation machinery, including 19 genes encoding ribosomal proteins, suggesting a reduction in ribosome synthesis. A total of 17 genes in the transcription functional category were also differentially regulated during starvation. Genes encoding core transcriptional machinery were among the 11 downregulated genes, including *rpoA* and *rpoZ* (6.2-fold and 3.5-fold lower, respectively), encoding RNA polymerase subunits; *rpoD* (3.7-fold lower), encoding the housekeeping sigma factor; and *nusB* (7.6-fold lower), encoding the transcription antitermination factor. Conversely, *csrA* (6.8-fold higher), encoding the carbon storage regulator, *dksA* (4.4-fold higher), and *rpoS* (3.8-fold higher), encoding the alternative sigma factor, were among the upregulated transcriptional regulator genes. In summary, levels of a large portion of RNA transcripts encoding crucial components of the replication, transcription, and translation machinery were decreased in wild-type spirochetes undergoing starvation. Given the functions encoded by these downregulated genes, our observations are consistent with stringent responses among other bacteria. None of the genes in these four functional categories listed above were differentially regulated in the Δ*dksA* mutant; therefore, the downregulation of these genes during starvation appears to be DksA dependent (Fig. 4D).

Typically, the stringent response activates the expression of genes encoding enzymes for amino acid synthesis, glycolysis, and persistence mechanisms. Consistent with the stringent response, B. burgdorferi spirochetes undergoing starvation also upregulate genes in the functional categories of translation, metabolism, and transcription. The expression of genes that potentially increase translational efficiency was upregulated (Table S3). These genes include *infA* (4.75-fold higher), encoding a translation initiation factor, *efp* (2.8-fold higher) and *tuf* (5.0-fold higher), encoding peptide elongation factors, and genes for five aminoacyl-tRNA synthetases required for the synthesis of Asp-tRNAasp, His-tRNA^His^, Ile-tRNA^Ile^, Leu-tRNA^Leu^, and Val-tRNA^Val^, which recognize 33% of codons utilized by B. burgdorferi open reading frames (71). The B. burgdorferi genome lacks many genes encoding amino acid biosynthesis pathways, and the bacterium imports oligopeptides into the cell through transporters to support protein synthesis. Four oligopeptide transporter genes were upregulated, *oppA5* (6.2-fold higher), *oppF* (5.8-fold higher), *oppD* (2.5-fold higher), and *oppB* (2.5-fold higher). The transcriptional profile of genes involved in translation and oligopeptide transport in the Δ*dksA* mutant did not overlap with the transcriptional profile of wild-type spirochetes during starvation. Additionally, wild-type spirochetes upregulated the following five genes encoding enzymes involved in glycolysis during starvation: *pfk* (2.4-fold higher), encoding 1-phosphofructokinase; *fbaA* (2.1-fold higher), encoding fructose-bisphosphate aldolase; *gapdh* (5.1-fold higher), encoding glyceraldehyde 3-phosphate dehydrogenase; *gmpA* (5.5-fold higher), encoding phosphoglycerate mutase; and *eno* (5.7-fold higher), encoding enolase. B. burgdorferi lacks an electron transport chain and ferments sugars to lactate for the generation of ATP. During starvation of wild-type spirochetes, no genes encoding enzymes involved in glycolysis or transporters for glucose, fructose, and chitobiose were downregulated. In contrast, the Δ*dksA* mutant strain exhibited lower transcript levels of genes encoding key glycolysis enzymes enolase (*eno*) and pyruvate kinase (*pyk*) during logarithmic growth. In addition, the Δ*dksA* mutant strain did not share the breadth of upregulation in genes encoding glycolysis enzymes in response to starvation compared to wild-type spirochetes.

### Increased expression of plasmid-carried genes in response to starvation conditions.

Wild-type spirochetes undergoing starvation also differentially expressed genes carried on the numerous circular and linear plasmids (Fig. 4C). Differentially expressed genes were largely limited to those encoding lipoproteins and hypothetical proteins, with 91% of those genes upregulated. These upregulated genes include those encoding nine OspE-related proteins (*erp*) and eight multicopy lipoproteins (*mlp*) carried on cp32s, with 3.1- to 9.8-fold and 4.8- to 13.1-fold upregulation, respectively (Table S3). Also upregulated were *revA* (6.4-fold higher) and *bbk32* (2.6-fold higher), encoding fibronectin-binding proteins. Specifically, the gene product of *bbk32* regulates the classical pathway of complement and is important for infection (72, 73). The biological significance of lipoprotein regulation during starvation in RPMI 1640 medium is unknown but likely is related to the interaction of the spirochete with its vector or hosts. Overall, protein expression and the level of immunogenic protein expression by wild-type and Δ*dksA* mutant spirochetes remain relatively constant following 6 h of incubation in RPMI 1640 medium (Fig. S3). Starvation is not thought to induce the mammalian infection-associated RpoN-RpoS cascade (1, 74, 75) and, as expected, transcription of the RpoS-regulated genes *dbpA* and *ospC* was not upregulated in response to nutrient limitation in wild-type spirochetes.

Compared to the wild-type strain, the Δ*dksA* mutant upregulated the expression of *revA*, *dbpA*, and *ospC* genes in response to starvation (Table S4). The Δ*dksA* mutant did not share the increased expression of *erp* or *mlp* genes with the wild-type strain during starvation. We investigated the possibility that these genes were constitutively upregulated in the Δ*dksA* mutant because the expression of many plasmid genes was higher than in the wild-type strains during logarithmic growth (Fig. 3B). A total of 41 plasmid-borne genes encoding lipoproteins were differentially expressed by the Δ*dksA* mutant during logarithmic growth (Tables S1 and S2). However, the *revA*, *bbk32*, *erp*, and *mlp* genes had no clear pattern of constitutively higher expression by the Δ*dksA* mutant strain. Moreover, we found that genes encoding lipoproteins under the control of RpoS regulation, which are important for B. burgdorferi transmission, such as *bba66*, *dbpA*, and *ospC*, were expressed at lower levels by the Δ*dksA* mutant during logarithmic growth. The stringent response regulator Rel_Bbu_ also regulates genes involved in transmission, including *rpoS*, *bosR*, and *ospC* (30). These results suggest DksA and the stringent response are required for the regulation of the transmission-associated lipoprotein genes *bba66*, *dbpA*, and *ospC*.

**FIG 5:**
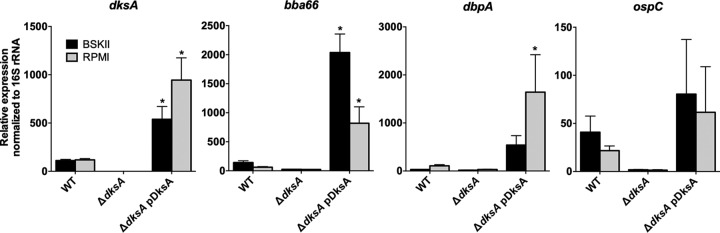
Overexpression of DksA in the Δ*dksA* pDksA mutant strain coincides with increased expression of plasmid-carried infectivity genes. RT-qPCR was performed on RNA extracted from wild-type (WT), Δ*dksA* mutant, and Δ*dksA* pDksA mutant mid-logarithmic-phase cultures (BSKII) and cultures starved in RPMI 1640 medium. Incubation in RPMI 1640 medium did not induce significant changes in expression of *dksA*, *bba66*, *dbpA*, or *ospC* for wild-type spirochetes. Error bars represent standard deviation calculated from four biological replicates. ANOVA with a Dunnett’s multiple-comparison test was performed for values between strains under BSK II and RPMI 1640 conditions. The asterisk indicates a *P* value of <0.01 for expression level comparison between WT and Δ*dksA* mutant or between WT and Δ*dksA* pDksA mutant.

To confirm that the disparate expression of *bba66*, *dbpA*, and *ospC* was DksA dependent, the expression of these genes was compared by RT-qPCR using RNA isolated from the wild-type, Δ*dksA* mutant, and Δ*dksA* pDksA mutant strains during logarithmic growth and under starvation conditions (Fig. 5). The expression of *bba66*, *dbpA*, and *ospC* was lower in the Δ*dksA* mutant than in the wild-type strain, indicating that regulation of these genes under starvation is not significant. In our complemented strain, the Δ*dksA* pDksA mutant, *dksA* was overexpressed, which coincided with higher levels of expression of the *bba66*, *dbpA*, and *ospC*. This observation supports the hypothesis that the expression of a subset of plasmid-encoded lipoproteins is either directly or indirectly dependent on DksA. The higher levels of *dksA* expression from the pDksA plasmid are consistent with the higher copy number of the parent shuttle vector (5 to 10 copies per genome) (76). Additionally, RT-qPCR was performed for *rpoD*, *fliZ*, and *ptsP* to evaluate the effects of *trans* complementation in the Δ*dksA* pDksA mutant strain. In the wild-type and Δ*dksA* pDksA strains, *rpoD*, *fliZ*, and *ptsP* are downregulated in response to starvation, while the Δ*dksA* mutant failed to similarly regulate these genes (Fig. S4A). RT-qPCR-based comparisons of gene expression between logarithmic growth and starvation conditions corroborated microarray findings and indicated that starvation-driven transcriptional regulation of chromosomally carried *rpoD*, *fliZ*, and *ptsP* was restored in the Δ*dksA* pDksA mutant strain. We also assayed for the restoration of glycerol utilization gene expression (Fig. S4B). While the Δ*dksA* mutant showed reduced levels of expression of *glpF* and *glpK* compared to the wild-type strain, the Δ*dksA* pDksA mutant strain did not exhibit restored expression of these genes, suggesting as-yet-unknown intricacies in their regulation. These results suggest that the cellular levels of DksA have the potential to play a key regulatory role in controlling plasmid-borne gene expression in B. burgdorferi.

### The Δ*dksA* mutant strain overproduces (p)ppGpp.

**FIG 6:**
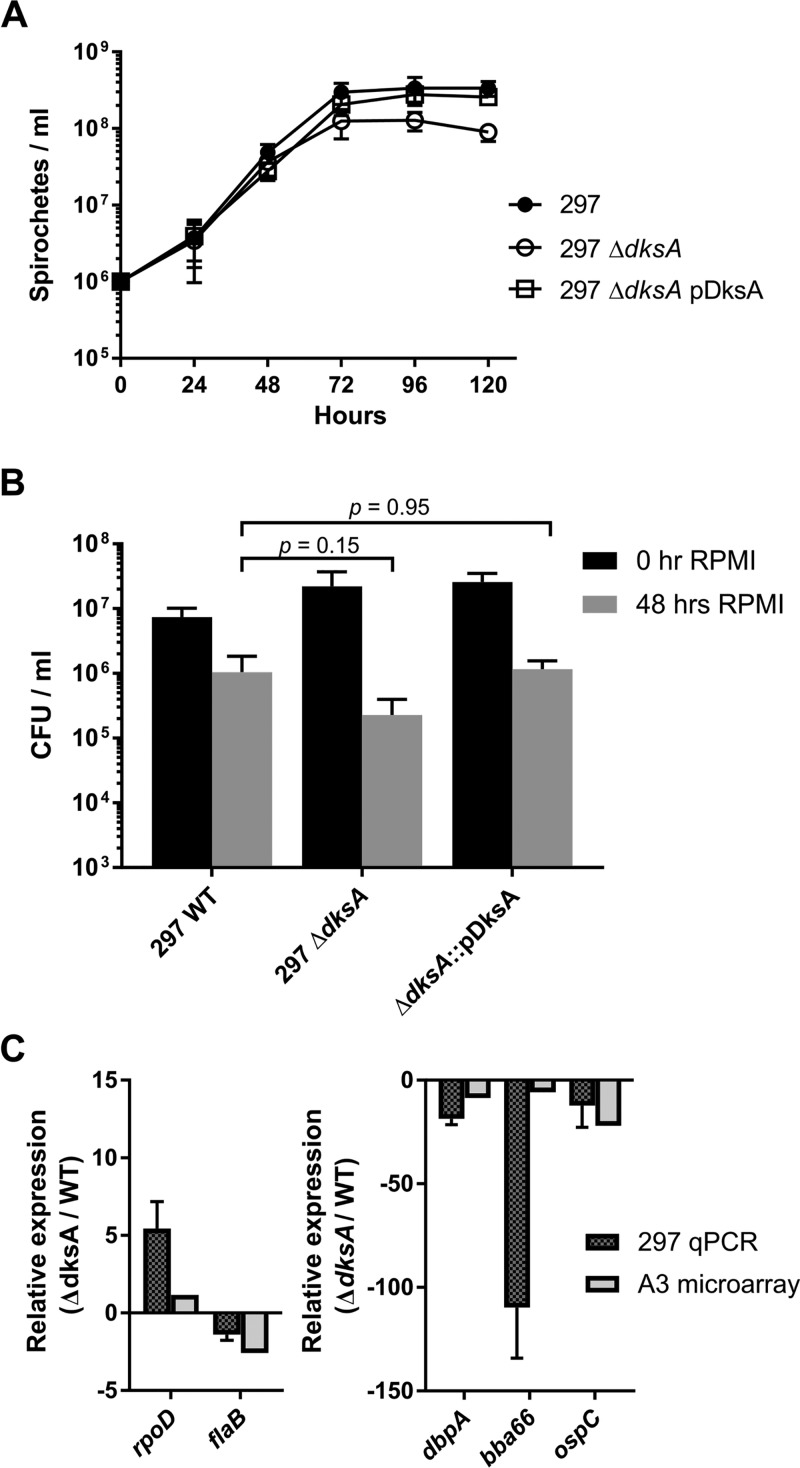
Evaluation of growth, RPMI 1640 survival, and relative RNA expression phenotypes for B. burgdorferi 297 wild-type (WT) and the 297 Δ*dksA* mutant strains. (A) Spirochetes were enumerated by microscopy. Values represent average from two replicates, and bars indicate standard deviation. (B) Mid-logarithmic-phase cultures of 297 wild-type, Δ*dksA* mutant, and Δ*dksA* pDksA mutant strains grown in BSK II medium were pelleted and resuspended in RPMI 1640 medium for 0 or 48 h before plating on semisolid BSK II medium, and CFU were enumerated following growth. The *P* values represent ANOVA with Dunnett’s multiple-comparison results from three replicate experiments. (C) Comparison of *dksA*-dependent gene expression in B31-A3 by microarray and 297 by RT-qPCR. Differential expression data of housekeeping genes (*rpoD* and *flaB*) and surface-expressed lipoprotein genes (*dbpA*, *bba66*, and *ospC*) are represented side by side. Relative expression values from RT-qPCR in the 297 strains represent 3 biological replicates and were normalized to 16S rRNA. Bars represent standard deviation.

**FIG 7:**
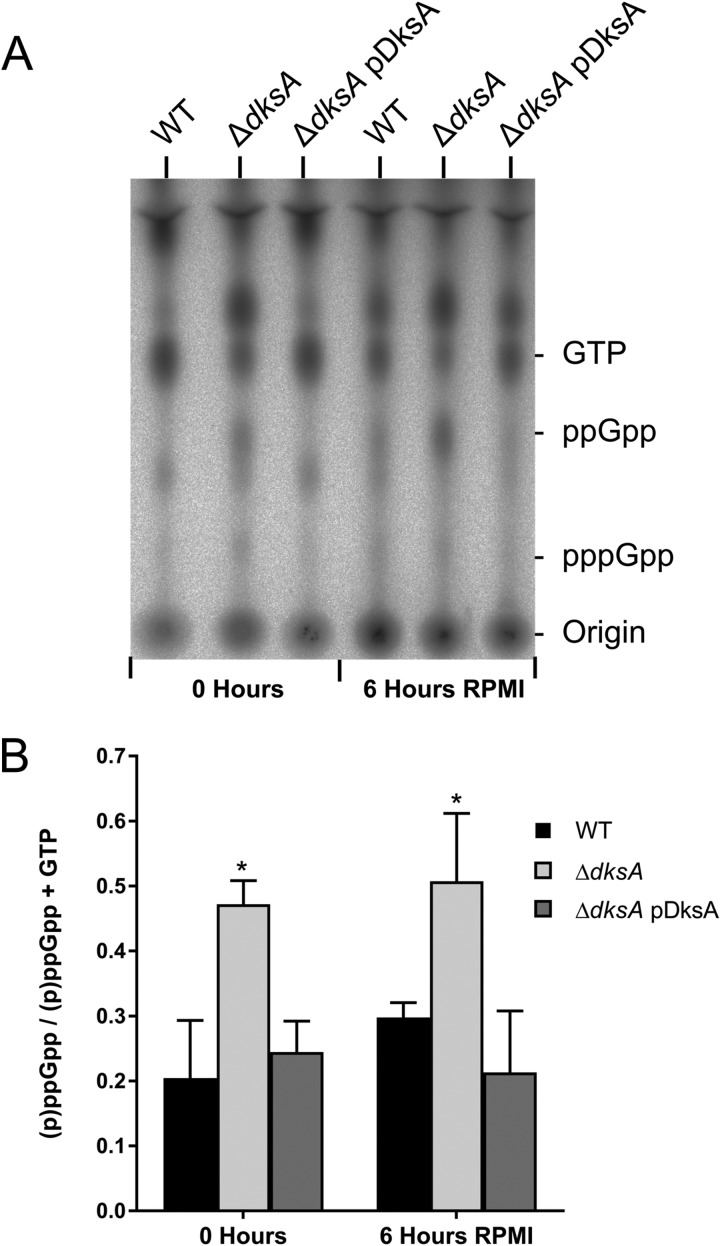
The Δ*dksA* mutant strain constitutively overproduces (p)ppGpp. (A) Representative TLC image for analysis of radiolabeled nucleotides from 297 wild-type (WT), Δ*dksA* mutant, and Δ*dksA* pDksA mutant strains cultured in BSK II medium with [^32^P]orthophosphate. Spirochetes were grown to 1 × 10^8^ spirochetes ml^−1^ (0 hours) and starved in RPMI 1640 medium (6 hours RPMI) before nucleotides were isolated and resolved by TLC. (B) Quantification of (p)ppGpp levels by densitometry. The values represent mean (p)ppGpp levels normalized to (p)ppGpp plus GTP from three independent experiments. Error bars represent standard deviation. Asterisks indicate *P* values of <0.05, as determined using one-way ANOVA with Tukey’s *post hoc* test.

The production of (p)ppGpp and transcriptional regulation of *dksA* are intertwined in E. coli, and (p)ppGpp also acts independently of DksA, resulting in transcriptional repression (56, 77). We measured the production of (p)ppGpp by thin-layer chromatography (TLC) in the B. burgdorferi 297 wild type, the isogenic Δ*dksA* mutant, and the complemented Δ*dksA* pDksA strain to test the potential interplay between (p)ppGpp production and DksA expression. Similar to the respective nonisogenic B. burgdorferi B31 A3 strains, B. burgdorferi 297 strains exhibit *dksA*-dependent phenotypes. The 297 Δ*dksA* mutant culture reaches lower densities during stationary phase (Fig. 6A). Following 48 h of starvation, the 297 Δ*dksA* mutant culture produces fewer CFU per milliliter, although these results only produced a *P* value of 0.15 in an analysis of variance (ANOVA) with Dunnett’s multiple-comparison test (Fig. 6B). When RNA expression levels by 297 Δ*dksA* and wild-type strains are compared by RT-qPCR, the direction of differential expression of *rpoD*, *flaB*, *dbpA*, *bba66*, and *ospC* genes was similar to that of the B31-A3 strain (Fig. 6C). The 297 background strains were cultured to early stationary phase (1 × 10^8^ spirochetes ml^−1^) in BSK II medium containing [^32^P]orthophosphate, and nucleotides were isolated before (0 h) or after starvation (6 h in RPMI 1640 medium) and separated by TLC. The amount of (p)ppGpp in each strain was quantified by scanning densitometry from three independent experiments (Fig. 7A), as previously described (30). While starvation in RPMI 1640 medium was previously demonstrated to increase (p)ppGpp in wild-type spirochetes, we did not detect statistically significant starvation-induced (p)ppGpp (Fig. 7B). We found that the Δ*dksA* mutant strain had significantly elevated levels of (p)ppGpp compared to the wild-type and complemented strains not only during starvation (6 h in RPMI 1640 medium) but also during growth in BSK II medium (0 h). The overproduction of (p)ppGpp in the Δ*dksA* mutant strain may represent a compensatory mechanism to overcome the loss of DksA. Given the 500 genes differentially regulated by wild-type spirochetes in response to starvation (Table S3), 186 of these genes were already similarly differentially expressed by the Δ*dksA* mutant strain relative to the wild-type strain during growth in BSK II medium. The microarray data suggest that while the Δ*dksA* mutant strain acts like a (p)ppGpp-deficient strain in the transcription of genes encoding the glycerol utilization pathway, oligopeptide transporters, ribosomal proteins, and others, the elevated levels of (p)ppGpp may play a role in the overall phenotype of the transcriptome in the Δ*dksA* mutant strain.

## DISCUSSION

We report that the B. burgdorferi genome encodes a 14.5-kDa DksA ortholog that is involved in the transcriptional response to nutrient limitation and regulation of plasmid-carried genes. The stringent response, mediated through (p)ppGpp, is required for B. burgdorferi to adapt to the changes between the host and vector environments, marking a shift in nutrient sources (30). Therefore, we set out to characterize the role of DksA as a transcriptional regulator of the B. burgdorferi stringent response by simulating transition from a nutrient-rich to a nutrient-limited environment. Our microarray results showed that transcript levels of 500 genes changed in response to nutrient limitation (see Table S3 in the supplemental material). The majority of the transcriptional changes were DksA dependent, with the expression of only 47 genes being DksA independent under nutrient-limiting conditions (Table S4). During mid-logarithmic growth, we found transcript levels of genes encoding ribosomal proteins (*rpmA*, *rplB, rplV, rpsS*, and *rpsC*) and stress response genes (*dnaK*, *dnaJ*, and *uvrB*) to be elevated in the Δ*dksA* mutant and the regulation of 41 plasmid-borne lipoprotein genes to be DksA dependent (Tables S1 and S2). The transcript levels of plasmid-carried lipoprotein genes *bba66*, *dbpA*, and *ospC* were independently confirmed to be DksA dependent in both the 297 and A3 backgrounds (Fig. 5 and 6), suggesting a pivotal role of DksA in expression of these genes. Moreover, the effects of a *dksA* deletion are likely not polar as complementation of the Δ*dksA* mutant strain with a plasmid encoding a FLAG epitope-tagged DksA led to rescue of the Δ*dksA* phenotype. B. burgdorferi possesses over 20 linear or circular plasmids (78, 79). A disproportionate number of outer membrane lipoproteins are encoded on these plasmids, and some have been shown to be required for virulence mechanisms, such as evasion of immune complement and antigenic variation (80–82). Regulation of these gene products can be complex, as exemplified by the expression of *ospC* on plasmid cp26, which is controlled by many factors, including plasmid supercoiling, oxygen levels, pH, and several transcriptional regulators (1–3, 83). This transcriptional study provides additional evidence that the stringent response plays a role in the regulation of control of outer membrane lipoproteins.

Our microarray analyses suggest a partial overlap between the DksA and the (p)ppGpp regulons of B. burgdorferi. The Δ*rel*_Bbu_ and Δ*dksA* mutants both express lower levels of oligopeptide transporter genes *oppA1* and *oppA2* and glycerol utilization genes *glpF* and *glpK*, while (p)ppGpp may independently regulate the glycerol utilization gene *glpD* (30, 31) (Table S2). The expression levels of the genes *ospA* and *lp6.6*, encoding tick-associated outer membrane proteins, and *napA*, an antioxidant defense gene, were reduced in Δ*dksA* mutants, suggesting that the regulation of these genes requires the cooperation of DksA and (p)ppGpp. In addition, the Δ*dksA* and Δ*rel*_Bbu_ mutants both display poor adaptation to starvation, since the number CFU during prolonged starvation in RPMI 1640 medium was reduced. Wild-type spirochetes reduce the transcription of replication, flagellar, and ribosomal genes in response to starvation in RPMI 1640 medium and, at the same time, upregulate genes required for protein synthesis and glycolysis. An explanation for the poor adaptation to starvation by Δ*dksA* and Δ*rel*_Bbu_ mutants is the inability of the mutant strains to reduce the transcription of growth- and motility-related genes to remain viable. The coordinated activity of DksA and (p)ppGpp is likely required for a proper response to starvation. In E. coli, DksA-dependent and (p)ppGpp-dependent regulation overlap to coordinate the starvation-induced stringent response (39, 56, 66–70).

**FIG 8:**
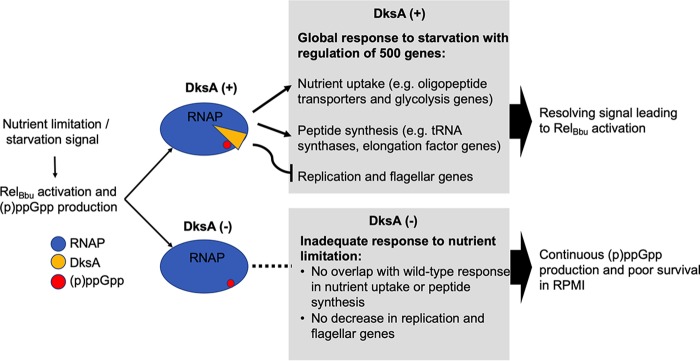
Model of the *B. burgdorferi* stringent response. Both DksA and (p)ppGpp interact with the RNA polymerase (RNAP) to exert transcriptional regulation under starvation conditions *in vitro*. In the absence of DksA, (p)ppGpp-dependent gene regulation appears largely lost, despite the apparent overproduction of (p)ppGpp in DksA-deficient *B. burgdorferi*.

The two regulators DksA and (p)ppGpp have a close regulatory relationship in B. burgdorferi. Two recent transcriptional studies of B. burgdorferi have demonstrated that Δ*rel*_Bbu_ mutants overexpress *dksA*, suggesting that the production of (p)ppGpp represses *dksA* (30, 31). While the role of *dksA* upregulation in Δ*rel*_Bbu_ mutant spirochetes is unclear, we now demonstrate that DksA plays a major role in transcriptional control of gene expression in B. burgdorferi. The transcriptomic data indicated the Δ*dksA* mutant exhibited expanded gene expression of select genes during mid-logarithmic growth and was unable to remodel the transcriptome during starvation. While the mechanism by which DksA imposes selectivity on gene transcription in B. burgdorferi remains to be explored, we found that DksA affects (p)ppGpp levels (Fig. 7). Levels of (p)ppGpp in the Δ*dksA* mutant were higher than levels in the wild-type cells during nutrient limitation. Moreover, Δ*dksA* mutant spirochetes produced these levels of (p)ppGpp prior to incubation in RPMI 1640 medium, suggesting altered Rel_Bbu_ activity in the absence of DksA. We propose that the stringent response in B. burgdorferi requires both DksA and (p)ppGpp (Fig. 8).

The DksA-dependent stringent response regulon potentially intersects with other regulatory mechanisms. The RNA-binding protein CsrA is thought to be a posttranscriptional regulator of motility genes (84, 85). Downregulation of expression of motility-associated genes, such as *flaB*, during starvation may occur through CsrA. Since (p)ppGpp is overproduced in the Δ*dksA* mutant, we cannot differentiate the effects of (p)ppGpp from DksA-dependent regulation. (p)ppGpp is known to act independently of transcription by interacting with GTPases and riboswitches (23, 86). Additionally, ATP and GTP homeostasis is likely altered by the consumption of these nucleoside triphosphates when (p)ppGpp is produced at high levels in the Δ*dksA* mutant. In addition, the genes encoding xanthine/guanine permease, a ribose/galactose ABC transporter, and adenine deaminases were also upregulated in the Δ*dksA* mutant (Table S1), potentially altering the flux of ATP or GTP pools. The genes encoding transmission-associated lipoproteins, *cspZ*, *ospD*, *mlpD*, and *ospE*, had higher expression in the Δ*dksA* mutant; the expression of these genes is known to be controlled by cyclic di-GMP produced by Rrp1 (87, 88). The regulation of cyclic-di-GMP synthesis may be altered in the Δ*dksA* mutant. Transcription of the infectivity-associated lipoprotein genes *ospC* and *dbpA* was decreased in the Δ*dksA* mutant. The *ospC* and *dbpA* genes are regulated through a complex regulatory cascade involving RpoN and RpoS (1, 74, 89, 90). As the production of (p)ppGpp alters phosphate homeostasis of in other bacteria (26, 91), a potential point of regulatory interplay is the response regulator Rrp2, which can regulate the RpoN-RpoS cascade (1, 90). The regulation of Rrp2 phosphorylation is currently unknown, but the alteration in the levels of phosphorylation in metabolic intermediates or in adenosine nucleotides may impact Rrp2 phosphorylation (92). Regulators sensitive to phosphate and nucleotide homeostasis in B. burgdorferi may contribute to the phenotype exhibited by the Δ*dksA* mutant.

The overall contribution of DksA to the transcriptional response of B. burgdorferi under nutrient-limited conditions may not be fully understood using the custom Affymetrix microarray platform used in this study, as both intergenic and antisense noncoding RNAs (ncRNAs) are not accounted for (32). Future transcriptomic studies using transcriptome sequencing (RNA-seq), which facilitate the identification and quantitation of ncRNAs, along with mRNAs, will expand the efforts presented in this current study to understand the global regulatory role of DksA in B. burgdorferi.

In summary, we found that the B. burgdorferi genome encodes a DksA that contains conserved amino acid resides in the coiled-coil tip and in the zinc finger important for DksA function in E. coli and Salmonella spp. The data presented here support the hypothesis that DksA is a functional transcriptional regulator in B. burgdorferi. We demonstrated *dksA*-dependent phenotypes in two strains of B. burgdorferi, B31-A3 and 297. The Δ*dksA* mutants in both B31-A3 and 297 backgrounds exhibit a long-term survival defect in RPMI 1640 medium and constitutively increased expression of housekeeping genes, such as *flaB* and *rpoD*. Finally, the DksA-dependent global transcriptional changes reported here suggest that DksA is fundamental for B. burgdorferi to adapt to environmental challenges invoking the stringent response. One caveat is that nutrient-limiting conditions used in this study do not fully mimic conditions *in vivo*, and further experiments in the animal model are required to more fully understand how DksA facilitates adaptation of B. burgdorferi to environmental challenges within the mammalian host and tick vector.

## MATERIALS AND METHODS

### Bacterial strains and growth conditions.

Low-passage-number B. burgdorferi B31-A3 (93) and 297 (94) strains, their respective *dksA* and *rel*_Bbu_ mutants, and a *trans*-complemented Δ*dksA* pDksA mutant strain were cultured in BSK II medium (50) at pH 7.6 under microaerobic conditions (5% CO_2_, 3% O_2_) at 34°C. BSK II medium was freshly prepared within 2 weeks prior to use and was inoculated with B. burgdorferi at a cell density of 1 × 10^6^ spirochetes ml^−1^ and grown to mid-logarithmic-phase (3 × 10^7^ to 5 × 10^7^ spirochetes ml^−1^) density. Spirochete densities were determined by dark-field microscopy, with eight fields counted per time point and four biological replicates. Cultures from frozen stocks were passaged two times before performing the assays. The construction of mutant strains is described in the supplemental material. The mutant strains and their plasmid profiles were determined by PCR analysis, as described previously (95, 96) (see Table S5 in the supplemental material).

### Incubation of spirochetes in RPMI 1640 medium.

Incubation of spirochetes in RPMI 1640 medium and growth in semisolid BSK II medium were determined under microaerobic conditions (5% CO_2_, 3% O_2_, 34°C). Mid-logarithmic-growth cultures were pelleted by centrifugation at 3,200 × *g* for 20 min at room temperature. The BSK II supernatant was discarded, and the pellet was resuspended in the original volume of RPMI 1640 medium (49) with 2.0 mM l-glutamine (Sigma-Aldrich, St. Louis, MO). The spirochetes were incubated for 6 h to compare transcription between strains or for 0 to 48 h to compare survival following long-term incubation. For quantification of viable spirochetes, B. burgdorferi were plated in 25 ml pg semisolid BSK II medium, as previously described (97), after culture density was reduced by serial dilutions in BSK II medium.

### RNA extraction.

Total RNA was extracted from 14-ml cultures at a density of 5 × 10^7^ spirochetes ml^−1^ in BSK II or RPMI 1640 medium. B. burgdorferi cells were pelleted by centrifugation at 4°C and 3,200 × *g* for 17 min. Pellets were washed once in HN buffer (10 mM HEPES, 10 mM NaCl [pH 8.0]) and then dissolved in 1 ml of RNAzol reagent (Sigma-Aldrich, St. Louis, MO) for RNA isolation according to the kit protocol. RNA integrity was confirmed by evaluation of rRNA following gel electrophoresis. The RNA was quantified by Take3 plate spectrophotometry in a Cytation 5 multimode plate reader (BioTek, Winooski, VT).

### RT-qPCR analysis.

cDNA synthesis was performed with approximately 1 µg of RNA with the RNA high-capacity cDNA reverse transcription kit (Applied Biosystems, Foster City, CA). The quantitative PCR (qPCR) amplification was performed in Bullseye EvaGreen master mix (MIDSCI, Valley Park, MO) using oligonucleotide primers specific to the gene of interest (Table S5) and detected using the CFX Connect real-time PCR detection system (Bio-Rad, Hercules, CA). All quantification cycle (*C_q_*) values were calculated by the CFX regression method. The *C_q_* values of raw RNA inputs into the cDNA reaction (minus the reverse transcriptase [RT] control) ensured that samples were DNA free. The 16S rRNA transcript levels were utilized as the reference. Typically, rRNA levels are significantly reduced during the stringent response, and DksA in E. coli has specifically been implicated in controlling the expression of *rrnB1*; however, the *C_q_* values of 16S rRNA were less responsive to various conditions and strains than other commonly used B. burgdorferi reference genes, such as *flaB* and *rpoD* (Fig. S4). The RT-qPCR data were analyzed in Excel (Microsoft, Redmond, WA) using the Δ*C_q_* method to represent transcript levels relative to 16S rRNA. Graphing and statistical comparisons were performed with Prism (GraphPad, La Jolla, CA).

### Microarray analysis.

Fragmented biotin-dUTP-labeled cDNA was prepared from purified RNA by following the Affymetrix (Santa Clara, CA) prokaryotic target preparation protocol. The cDNA was hybridized to an Affymetrix-based Rocky Mountain Lab custom chip 1. Each Affymetrix chip contains three intrachip locations for strand-specific 16 antisense perfect match and mismatch probe sets against each of the 1,323 open reading frames (ORFs) of the B. burgdorferi strain B31 genome. One chip was used to assay for the transcriptome per biological sample. Initial quality analysis was performed on the Affymetrix Command Console version 3.1, and hybridization signals were normalized by the Affymetrix expression console version 1.1.2800 using scaling based on average cell intensity. Average normalized signal intensities for an ORF from three intrachip locations and three biological replicates (a total of 9 observations) were used for subsequent calculations. Signal intensity principal-component analysis was performed using Genomics Suite software version 6.6 6.13.213 (Partek, St. Louis, MO), verifying that variability among biological replicates remained small compared to variability between strains and conditions. An ANOVA was performed within the Partek Genomic Suite to obtain multiple test-corrected *P* values using the false-discovery rate method, with a threshold of 5% false-discovery rate (98). Fold change values and signal confidence were calculated in custom Excel templates. Importantly, our Δ*dksA* mutant strain lacked lp-5, -21, -25, and -28-4 plasmids, and the chip hybridization locations for these plasmids were excluded from the analysis.

The number of genes regulated in genomic locations or in functional categories was quantified using filters coded in RStudio (Boston, MA). Affymetrix probe sets representing the gene comparisons with above-background signal, ANOVA value (*P* < 0.05), and relative expression difference of 2-fold or more were selected for representation. The number of genes that passed the criteria were totaled for each genomic segment, or alternatively, each higher- or lower-expressed gene was categorized by gene ontology, as previously described (30, 31). The total gene numbers were visualized with Prism (GraphPad, La Jolla, CA).

### SDS-PAGE and immunoblotting.

Total cell lysates were prepared from 45-ml cultures. Spirochetes were pelleted at 4°C and 3,200 × *g* for 17 min. Spirochetes were washed twice with HN buffer (10 mM HEPES, 10 mM NaCl [pH 8.0]) and subsequently lysed in lysis buffer (4% SDS, 0.1 M Tris-HCl [pH 8.0]). The lysate loading was equalized to 4 µg per sample, roughly 5 × 10^7^ spirochetes, by a bicinchoninic acid (BCA) assay (Thermo Fisher Scientific, Grand Island, NY). SDS-PAGE was performed on the Mini-Tetra system (Bio-Rad, Hercules, CA). Proteins were detected using the EzStain system on the Gel Doc EZ imager (Bio-Rad). Protein was transferred to a polyvinylidene difluoride (PVDF) membrane with the Transblot Turbo system (Bio-Rad). The DYKDDDDK(FLAG) tag monoclonal mouse antibody, at 1 µg ml^−1^, (Thermo Fisher Scientific) was diluted 1:2,000 in Tris-buffered saline with Tween 20 (TBST) for blotting for recombinant protein detection. Rabbit anti-DksA antibody was diluted at 1:2,000 in TBST for DksA protein detection (GenScript, Piscataway, NJ). Mouse serum from B31-A3-infected mice was diluted 1:200 for immunogenic protein blotting. The antibody binding was detected with the addition of horseradish peroxidase (HRP)-conjugated secondary antibody and subsequent imaging using ECL chemiluminescence substrate (Li-Cor, Lincoln, NE) and the ChemiDoc imaging system (Bio-Rad).

### Measurement of (p)ppGpp.

Relative quantities of (p)ppGpp were measured by TLC of radiolabeled nucleotides, as previously described (30). B. burgdorferi 297 wild-type, the isogenic Δ*dksA* mutant, and Δ*dksA* pDksA mutant strains were cultured to 1 × 10^8^ spirochetes ml^−1^ in BSK II medium containing 20 µCi/ml [^32^P]orthophosphate (PerkinElmer, Waltham, MA) in 500 µl, pelleted by centrifugation at 9,000 × *g* for 7 min, and resuspended in RPMI 1640 medium. Cultures were collected by centrifugation at 20,800 × *g* for 5 min at 4°C, cells were washed once with Dulbecco’s phosphate-buffered saline (dPBS), and the cell pellet was lysed with 6.5 M formic acid (Thermo Fisher Scientific, Grand Island, NY). Cell debris was removed by centrifugation at 20,800 × *g* for 5 min at 4°C. The nucleotides were separated by TLC on polyethylenimine cellulose plates (EMD Millipore, Burlington, MA) in 1.5 M KH_2_PO_4_ (pH 3.4) buffer. After drying the TLC plates, radioactivity was detected by a 48- to 72-h exposure to an intensifying screen, and screens were imaged by a FLA-3000G phosphorimager (Fujifilm Life Sciences, Stamford, CT). Values are expressed as the ratio (p)ppGpp/[(p)ppGpp + GTP] from the densitometry measurements from three independent experiments. The mean values from three independent experiments were analyzed using one-way ANOVA and Tukey’s *post hoc* test to determine if differences were statistically significant.

### Data availability.

The microarray data have been submitted to the Gene Expression Omnibus (GEO accession number GSE119023).

## Supplementary Material

Supplemental file 1

## References

[1] Samuels DS 2011 Gene regulation in *Borrelia burgdorferi*. Annu Rev Microbiol 65:479–499. doi:10.1146/annurev.micro.112408.134040. 21801026

[2] Radolf JD, Caimano MJ, Stevenson B, Hu LT 2012 Of ticks, mice and men: understanding the dual-host lifestyle of Lyme disease spirochaetes. Nat Rev Microbiol 10:87–99. doi:10.1038/nrmicro2714. 22230951 PMC3313462

[3] Caimano MJ, Drecktrah D, Kung F, Samuels DS 2016 Interaction of the Lyme disease spirochete with its tick vector. Cell Microbiol 18:919–927. doi:10.1111/cmi.12609. 27147446 PMC5067140

[4] Gray JS, Kahl O, Lane RS, Levin ML, Tsao JI 2016 Diapause in ticks of the medically important *Ixodes ricinus* species complex. Ticks Tick Borne Dis 7:992–1003. doi:10.1016/j.ttbdis.2016.05.006. 27263092 PMC5659180

[5] Sonenshine DE 1991 Biology of ticks. Oxford University Press, New York, NY.

[6] Bourret TJ, Lawrence KA, Shaw JA, Lin T, Norris SJ, Gherardini FC 2016 The nucleotide excision repair pathway protects *Borrelia burgdorferi* from nitrosative stress in *Ixodes scapularis* ticks. Front Microbiol 7:1397. doi:10.3389/fmicb.2016.01397. 27656169 PMC5013056

[7] Bontemps-Gallo S, Lawrence K, Gherardini FC 2016 Two different virulence-related regulatory pathways in *Borrelia burgdorferi* are directly affected by osmotic fluxes in the blood meal of feeding Ixodes ticks. PLoS Pathog 12:e1005791. doi:10.1371/journal.ppat.1005791. 27525653 PMC4985143

[8] Dulebohn DP, Richards CL, Su H, Lawrence KA, Gherardini FC 2017 Weak organic acids decrease *Borrelia burgdorferi* cytoplasmic pH, eliciting an acid stress response and impacting RpoN- and RpoS-dependent gene expression. Front Microbiol 8:1734. doi:10.3389/fmicb.2017.01734. 29033900 PMC5626856

[9] Iyer R, Caimano MJ, Luthra A, Axline D Jr, Corona A, Iacobas DA, Radolf JD, Schwartz I 2015 Stage-specific global alterations in the transcriptomes of Lyme disease spirochetes during tick feeding and following mammalian host adaptation. Mol Microbiol 95:509–538. doi:10.1111/mmi.12882. 25425211 PMC4429771

[10] Gulia-Nuss M, Nuss AB, Meyer JM, Sonenshine DE, Roe RM, Waterhouse RM, Sattelle DB, de la Fuente J, Ribeiro JM, Megy K, Thimmapuram J, Miller JR, Walenz BP, Koren S, Hostetler JB, Thiagarajan M, Joardar VS, Hannick LI, Bidwell S, Hammond MP, Young S, Zeng Q, Abrudan JL, Almeida FC, Ayllón N, Bhide K, Bissinger BW, Bonzon-Kulichenko E, Buckingham SD, Caffrey DR, Caimano MJ, Croset V, Driscoll T, Gilbert D, Gillespie JJ, Giraldo-Calderón GI, Grabowski JM, Jiang D, Khalil SMS, Kim D, Kocan KM, Koči J, Kuhn RJ, Kurtti TJ, Lees K, Lang EG, Kennedy RC, Kwon H, Perera R, Qi Y, 2016 Genomic insights into the *Ixodes scapularis* tick vector of Lyme disease. Nat Commun 7:10507. doi:10.1038/ncomms10507. 26856261 PMC4748124

[11] Iyer R, Schwartz I 2016 Microarray-based comparative genomic and transcriptome analysis of *Borrelia burgdorferi*. Microarrays (Basel) 5:E9. 27600075 10.3390/microarrays5020009 PMC5003485

[12] Groshong AM, Dey A, Bezsonova I, Caimano MJ, Radolf JD 2017 Peptide uptake is essential for *Borrelia burgdorferi* viability and involves structural and regulatory complexity of its oligopeptide transporter. mBio 8:e02047-17. 29259089 10.1128/mBio.02047-17 PMC5736914

[13] Fraser CM, Casjens S, Huang WM, Sutton GG, Clayton R, Lathigra R, White O, Ketchum KA, Dodson R, Hickey EK, Gwinn M, Dougherty B, Tomb JF, Fleischmann RD, Richardson D, Peterson J, Kerlavage AR, Quackenbush J, Salzberg S, Hanson M, van Vugt R, Palmer N, Adams MD, Gocayne J, Weidman J, Utterback T, Watthey L, McDonald L, Artiach P, Bowman C, Garland S, Fuji C, Cotton MD, Horst K, Roberts K, Hatch B, Smith HO, Venter JC 1997 Genomic sequence of a Lyme disease spirochaete, *Borrelia burgdorferi*. Nature 390:580–586. doi:10.1038/37551. 9403685

[14] Gherardini FC, Boylan JA, Lawrence KA, Skare JT 2010 Metabolism and physiology of *Borrelia*, p 103–138. *In* Samuels DS, Radolf JD (ed), Borrelia: molecular biology, host interaction and pathogenesis. Caister Academic Press, Norfolk, United Kingdom.

[15] Pappas CJ, Iyer R, Petzke MM, Caimano MJ, Radolf JD, Schwartz I 2011 *Borrelia burgdorferi* requires glycerol for maximum fitness during the tick phase of the enzootic cycle. PLoS Pathog 7:e1002102. doi:10.1371/journal.ppat.1002102. 21750672 PMC3131272

[16] Tilly K, Elias AF, Errett J, Fischer E, Iyer R, Schwartz I, Bono JL, Rosa P 2001 Genetics and regulation of chitobiose utilization in *Borrelia burgdorferi*. J Bacteriol 183:5544–5553. doi:10.1128/JB.183.19.5544-5553.2001. 11544216 PMC95445

[17] He M, Ouyang Z, Troxell B, Xu H, Moh A, Piesman J, Norgard MV, Gomelsky M, Yang XF 2011 Cyclic di-GMP is essential for the survival of the Lyme disease spirochete in ticks. PLoS Pathog 7:e1002133. doi:10.1371/journal.ppat.1002133. 21738477 PMC3128128

[18] Li X, Pal U, Ramamoorthi N, Liu X, Desrosiers DC, Eggers CH, Anderson JF, Radolf JD, Fikrig E 2007 The Lyme disease agent *Borrelia burgdorferi* requires BB0690, a Dps homologue, to persist within ticks. Mol Microbiol 63:694–710. doi:10.1111/j.1365-2958.2006.05550.x. 17181780

[19] Promnares K, Kumar M, Shroder DY, Zhang X, Anderson JF, Pal U 2009 *Borrelia burgdorferi* small lipoprotein Lp6.6 is a member of multiple protein complexes in the outer membrane and facilitates pathogen transmission from ticks to mice. Mol Microbiol 74:112–125. doi:10.1111/j.1365-2958.2009.06853.x. 19703109 PMC2754595

[20] Patton TG, Brandt KS, Nolder C, Clifton DR, Carroll JA, Gilmore RD 2013 *Borrelia burgdorferi bba66* gene inactivation results in attenuated mouse infection by tick transmission. Infect Immun 81:2488–2498. doi:10.1128/IAI.00140-13. 23630963 PMC3697616

[21] Hardy PO, Chaconas G 2013 The nucleotide excision repair system of *Borrelia burgdorferi* is the sole pathway involved in repair of DNA damage by UV light. J Bacteriol 195:2220–2231. doi:10.1128/JB.00043-13. 23475971 PMC3650546

[22] Troxell B, Yang XF 2013 Metal-dependent gene regulation in the causative agent of Lyme disease. Front Cell Infect Microbiol 3:79. doi:10.3389/fcimb.2013.00079. 24298449 PMC3828560

[23] Steinchen W, Bange G 2016 The magic dance of the alarmones (p)ppGpp. Mol Microbiol 101:531–544. doi:10.1111/mmi.13412. 27149325

[24] Traxler MF, Summers SM, Nguyen HT, Zacharia VM, Hightower GA, Smith JT, Conway T 2008 The global, ppGpp-mediated stringent response to amino acid starvation in *Escherichia coli*. Mol Microbiol 68:1128–1148. doi:10.1111/j.1365-2958.2008.06229.x. 18430135 PMC3719176

[25] Potrykus K, Cashel M 2008 (p)ppGpp: still magical? Annu Rev Microbiol 62:35–51. doi:10.1146/annurev.micro.62.081307.162903. 18454629

[26] Hauryliuk V, Atkinson GC, Murakami KS, Tenson T, Gerdes K 2015 Recent functional insights into the role of (p)ppGpp in bacterial physiology. Nat Rev Microbiol 13:298–309. doi:10.1038/nrmicro3448. 25853779 PMC4659695

[27] Durfee T, Hansen AM, Zhi H, Blattner FR, Jin DJ 2008 Transcription profiling of the stringent response in *Escherichia coli*. J Bacteriol 190:1084–1096. doi:10.1128/JB.01092-07. 18039766 PMC2223561

[28] Paul BJ, Berkmen MB, Gourse RL 2005 DksA potentiates direct activation of amino acid promoters by ppGpp. Proc Natl Acad Sci U S A 102:7823–7828. doi:10.1073/pnas.0501170102. 15899978 PMC1142371

[29] Gentry DR, Hernandez VJ, Nguyen LH, Jensen DB, Cashel M 1993 Synthesis of the stationary-phase sigma factor sigma s is positively regulated by ppGpp. J Bacteriol 175:7982–7989. doi:10.1128/jb.175.24.7982-7989.1993. 8253685 PMC206978

[30] Drecktrah D, Lybecker M, Popitsch N, Rescheneder P, Hall LS, Samuels DS 2015 The *Borrelia burgdorferi* RelA/SpoT homolog and stringent response regulate survival in the tick vector and global gene expression during starvation. PLoS Pathog 11:e1005160. doi:10.1371/journal.ppat.1005160. 26371761 PMC4570706

[31] Bugrysheva JV, Pappas CJ, Terekhova DA, Iyer R, Godfrey HP, Schwartz I, Cabello FC 2015 Characterization of the Rel_Bbu_ regulon in *Borrelia burgdorferi* reveals modulation of glycerol metabolism by (p)ppGpp. PLoS One 10:e0118063. doi:10.1371/journal.pone.0118063. 25688856 PMC4331090

[32] Drecktrah D, Hall LS, Rescheneder P, Lybecker M, Samuels DS 2018 The stringent response-regulated sRNA transcriptome of *Borrelia burgdorferi*. Front Cell Infect Microbiol 8:231. doi:10.3389/fcimb.2018.00231. 30027068 PMC6041397

[33] Concepcion MB, Nelson DR 2003 Expression of *spoT* in *Borrelia burgdorferi* during serum starvation. J Bacteriol 185:444–452. doi:10.1128/JB.185.2.444-452.2003. 12511489 PMC145309

[34] Dalebroux ZD, Svensson SL, Gaynor EC, Swanson MS 2010 ppGpp conjures bacterial virulence. Microbiol Mol Biol Rev 74:171–199. doi:10.1128/MMBR.00046-09. 20508246 PMC2884408

[35] Kvint K, Farewell A, Nystrom T 2000 RpoS-dependent promoters require guanosine tetraphosphate for induction even in the presence of high levels of sigma(s). J Biol Chem 275:14795–14798. doi:10.1074/jbc.C000128200. 10747855

[36] Brown L, Gentry D, Elliott T, Cashel M 2002 DksA affects ppGpp induction of RpoS at a translational level. J Bacteriol 184:4455–4465. doi:10.1128/JB.184.16.4455-4465.2002. 12142416 PMC135238

[37] Bernardo LM, Johansson LU, Skarfstad E, Shingler V 2009 sigma54-promoter discrimination and regulation by ppGpp and DksA. J Biol Chem 284:828–838. doi:10.1074/jbc.M807707200. 19008221

[38] Łyżeń R, Maitra A, Milewska K, Kochanowska-Łyżeń M, Hernandez VJ, Szalewska-Pałasz A 2016 The dual role of DksA protein in the regulation of *Escherichia coli* pArgX promoter. Nucleic Acids Res 44:10316–10325. doi:10.1093/nar/gkw912. 27915292 PMC5137449

[39] Magnusson LU, Gummesson B, Joksimovic P, Farewell A, Nystrom T 2007 Identical, independent, and opposing roles of ppGpp and DksA in *Escherichia coli*. J Bacteriol 189:5193–5202. doi:10.1128/JB.00330-07. 17496080 PMC1951846

[40] Dalebroux ZD, Yagi BF, Sahr T, Buchrieser C, Swanson MS 2010 Distinct roles of ppGpp and DksA in *Legionella pneumophila* differentiation. Mol Microbiol 76:200–219. doi:10.1111/j.1365-2958.2010.07094.x. 20199605 PMC2908999

[41] Holley CL, Zhang X, Fortney KR, Ellinger S, Johnson P, Baker B, Liu Y, Janowicz DM, Katz BP, Munson RS Jr, Spinola SM 2015 DksA and (p)ppGpp have unique and overlapping contributions to *Haemophilus ducreyi* pathogenesis in humans. Infect Immun 83:3281–3292. doi:10.1128/IAI.00692-15. 26056381 PMC4496623

[42] Pal RR, Bag S, Dasgupta S, Das B, Bhadra RK 2012 Functional characterization of the stringent response regulatory gene *dksA* of *Vibrio cholerae* and its role in modulation of virulence phenotypes. J Bacteriol 194:5638–5648. doi:10.1128/JB.00518-12. 22904284 PMC3458680

[43] Sharma AK, Payne SM 2006 Induction of expression of *hfq* by DksA is essential for *Shigella flexneri* virulence. Mol Microbiol 62:469–479. doi:10.1111/j.1365-2958.2006.05376.x. 17020583

[44] Nakanishi N, Abe H, Ogura Y, Hayashi T, Tashiro K, Kuhara S, Sugimoto N, Tobe T 2006 ppGpp with DksA controls gene expression in the locus of enterocyte effacement (LEE) pathogenicity island of enterohaemorrhagic *Escherichia coli* through activation of two virulence regulatory genes. Mol Microbiol 61:194–205. doi:10.1111/j.1365-2958.2006.05217.x. 16824105

[45] Branny P, Pearson JP, Pesci EC, Kohler T, Iglewski BH, Van Delden C 2001 Inhibition of quorum sensing by a *Pseudomonas aeruginosa dksA* homologue. J Bacteriol 183:1531–1539. doi:10.1128/JB.183.5.1531-1539.2001. 11160083 PMC95037

[46] Henard CA, Vázquez-Torres A 2012 DksA-dependent resistance of Salmonella enterica serovar Typhimurium against the antimicrobial activity of inducible nitric oxide synthase. Infect Immun 80:1373–1380. doi:10.1128/IAI.06316-11. 22311927 PMC3318398

[47] Webb C, Moreno M, Wilmes-Riesenberg M, Curtiss R III, Foster JW 1999 Effects of DksA and ClpP protease on sigma S production and virulence in *Salmonella* Typhimurium. Mol Microbiol 34:112–123. doi:10.1046/j.1365-2958.1999.01581.x. 10540290

[48] Moore GE 1969 Lymphoblastoid cell lines from normal persons and those with nonmalignant diseases. J Surg Res 9:139–141. doi:10.1016/0022-4804(69)90044-4. 5774617

[49] Moore GE, Gerner RE, Franklin HA 1967 Culture of normal human leukocytes. JAMA 199:519–524. doi:10.1001/jama.1967.03120080053007. 4960081

[50] Barbour AG 1984 Isolation and cultivation of Lyme disease spirochetes. Yale J Biol Med 57:521–525. 6393604 PMC2589996

[51] Livengood JA, Schmit VL, Gilmore RD Jr. 2008 Global transcriptome analysis of *Borrelia burgdorferi* during association with human neuroglial cells. Infect Immun 76:298–307. doi:10.1128/IAI.00866-07. 17984208 PMC2223675

[52] Voyich JM, Braughton KR, Sturdevant DE, Whitney AR, Said-Salim B, Porcella SF, Long RD, Dorward DW, Gardner DJ, Kreiswirth BN, Musser JM, DeLeo FR 2005 Insights into mechanisms used by *Staphylococcus aureus* to avoid destruction by human neutrophils. J Immunol 175:3907–3919. doi:10.4049/jimmunol.175.6.3907. 16148137

[53] Sumby P, Whitney AR, Graviss EA, DeLeo FR, Musser JM 2006 Genome-wide analysis of group a *streptococci* reveals a mutation that modulates global phenotype and disease specificity. PLoS Pathog 2:e5. doi:10.1371/journal.ppat.0020005. 16446783 PMC1354197

[54] Sebbane F, Lemaitre N, Sturdevant DE, Rebeil R, Virtaneva K, Porcella SF, Hinnebusch BJ 2006 Adaptive response of *Yersinia pestis* to extracellular effectors of innate immunity during bubonic plague. Proc Natl Acad Sci U S A 103:11766–11771. doi:10.1073/pnas.0601182103. 16864791 PMC1518801

[55] Blaby-Haas CE, Furman R, Rodionov DA, Artsimovitch I, de C-, Lagard V 2011 Role of a Zn-independent DksA in Zn homeostasis and stringent response. Mol Microbiol 79:700–715. doi:10.1111/j.1365-2958.2010.07475.x. 21255113 PMC3076637

[56] Ross W, Sanchez-Vazquez P, Chen AY, Lee JH, Burgos HL, Gourse RL 2016 ppGpp binding to a site at the RNAP-DksA interface accounts for its dramatic effects on transcription initiation during the stringent response. Mol Cell 62:811–823. doi:10.1016/j.molcel.2016.04.029. 27237053 PMC4912440

[57] Perederina A, Svetlov V, Vassylyeva MN, Tahirov TH, Yokoyama S, Artsimovitch I, Vassylyev DG 2004 Regulation through the secondary channel–structural framework for ppGpp-DksA synergism during transcription. Cell 118:297–309. doi:10.1016/j.cell.2004.06.030. 15294156

[58] Lennon CW, Ross W, Martin-Tumasz S, Toulokhonov I, Vrentas CE, Rutherford ST, Lee JH, Butcher SE, Gourse RL 2012 Direct interactions between the coiled-coil tip of DksA and the trigger loop of RNA polymerase mediate transcriptional regulation. Genes Dev 26:2634–2646. doi:10.1101/gad.204693.112. 23207918 PMC3521624

[59] Furman R, Tsodikov OV, Wolf YI, Artsimovitch I 2013 An insertion in the catalytic trigger loop gates the secondary channel of RNA polymerase. J Mol Biol 425:82–93. doi:10.1016/j.jmb.2012.11.008. 23147217 PMC5522729

[60] Henard CA, Tapscott T, Crawford MA, Husain M, Doulias PT, Porwollik S, Liu L, McClelland M, Ischiropoulos H, Vazquez-Torres A 2014 The 4-cysteine zinc-finger motif of the RNA polymerase regulator DksA serves as a thiol switch for sensing oxidative and nitrosative stress. Mol Microbiol 91:790–804. doi:10.1111/mmi.12498. 24354846 PMC4053250

[61] Crawford MA, Tapscott T, Fitzsimmons LF, Liu L, Reyes AM, Libby SJ, Trujillo M, Fang FC, Radi R, Vazquez-Torres A 2016 Redox-active sensing by bacterial DksA transcription factors is determined by cysteine and zinc content. mBio 7:e02161-15. doi:10.1128/mBio.02161-15. 27094335 PMC4850274

[62] Zimmermann L, Stephens A, Nam SZ, Rau D, Kubler J, Lozajic M, Gabler F, Soding J, Lupas AN, Alva V 2018 A completely reimplemented MPI bioinformatics toolkit with a new HHpred server at its core. J Mol Biol 430:2237–2243. doi:10.1016/j.jmb.2017.12.007. 29258817

[63] Larkin MA, Blackshields G, Brown NP, Chenna R, McGettigan PA, McWilliam H, Valentin F, Wallace IM, Wilm A, Lopez R, Thompson JD, Gibson TJ, Higgins DG 2007 Clustal W and Clustal X version 2.0. Bioinformatics 23:2947–2948. doi:10.1093/bioinformatics/btm404. 17846036

[64] Elias AF, Bono JL, Kupko JJ III, Stewart PE, Krum JG, Rosa PA 2003 New antibiotic resistance cassettes suitable for genetic studies in *Borrelia burgdorferi*. J Mol Microbiol Biotechnol 6:29–40. doi:10.1159/000073406. 14593251

[65] Schneider CA, Rasband WS, Eliceiri KW 2012 NIH Image to ImageJ: 25 years of image analysis. Nat Methods 9:671–675. doi:10.1038/nmeth.2089. 22930834 PMC5554542

[66] Paul BJ, Barker MM, Ross W, Schneider DA, Webb C, Foster JW, Gourse RL 2004 DksA: a critical component of the transcription initiation machinery that potentiates the regulation of rRNA promoters by ppGpp and the initiating NTP. Cell 118:311–322. doi:10.1016/j.cell.2004.07.009. 15294157

[67] Lemke JJ, Durfee T, Gourse RL 2009 DksA and ppGpp directly regulate transcription of the *Escherichia coli* flagellar cascade. Mol Microbiol 74:1368–1379. doi:10.1111/j.1365-2958.2009.06939.x. 19889089 PMC2806482

[68] Åberg A, Fernández-Vázquez J, Cabrer-Panes JD, Sánchez A, Balsalobre C 2009 Similar and divergent effects of ppGpp and DksA deficiencies on transcription in *Escherichia coli*. J Bacteriol 191:3226–3236. doi:10.1128/JB.01410-08. 19251846 PMC2687150

[69] Furman R, Danhart EM, NandyMazumdar M, Yuan C, Foster MP, Artsimovitch I 2015 pH dependence of the stress regulator DksA. PLoS One 10:e0120746. doi:10.1371/journal.pone.0120746. 25799498 PMC4370453

[70] Gourse RL, Chen AY, Gopalkrishnan S, Sanchez-Vazquez P, Myers A, Ross W 2018 Transcriptional responses to ppGpp and DksA. Annu Rev Microbiol 72:163–184. doi:10.1146/annurev-micro-090817-062444. 30200857 PMC6586590

[71] Lafay B, Lloyd AT, McLean MJ, Devine KM, Sharp PM, Wolfe KH 1999 Proteome composition and codon usage in spirochaetes: species-specific and DNA strand-specific mutational biases. Nucleic Acids Res 27:1642–1649. doi:10.1093/nar/27.7.1642. 10075995 PMC148367

[72] Lin YP, Chen Q, Ritchie JA, Dufour NP, Fischer JR, Coburn J, Leong JM 2015 Glycosaminoglycan binding by *Borrelia burgdorferi* adhesin BBK32 specifically and uniquely promotes joint colonization. Cell Microbiol 17:860–875. doi:10.1111/cmi.12407. 25486989 PMC4437914

[73] Garcia BL, Zhi H, Wager B, Hook M, Skare JT 2016 *Borrelia burgdorferi* BBK32 inhibits the classical pathway by blocking activation of the C1 complement complex. PLoS Pathog 12:e1005404. doi:10.1371/journal.ppat.1005404. 26808924 PMC4725857

[74] Burtnick MN, Downey JS, Brett PJ, Boylan JA, Frye JG, Hoover TR, Gherardini FC 2007 Insights into the complex regulation of *rpoS* in *Borrelia burgdorferi*. Mol Microbiol 65:277–293. doi:10.1111/j.1365-2958.2007.05813.x. 17590233 PMC1976401

[75] Caimano MJ, Iyer R, Eggers CH, Gonzalez C, Morton EA, Gilbert MA, Schwartz I, Radolf JD 2007 Analysis of the RpoS regulon in *Borrelia burgdorferi* in response to mammalian host signals provides insight into RpoS function during the enzootic cycle. Mol Microbiol 65:1193–1217. doi:10.1111/j.1365-2958.2007.05860.x. 17645733 PMC2967192

[76] Tilly K, Krum JG, Bestor A, Jewett MW, Grimm D, Bueschel D, Byram R, Dorward D, Vanraden MJ, Stewart P, Rosa P 2006 *Borrelia burgdorferi* OspC protein required exclusively in a crucial early stage of mammalian infection. Infect Immun 74:3554–3564. doi:10.1128/IAI.01950-05. 16714588 PMC1479285

[77] Chandrangsu P, Lemke JJ, Gourse RL 2011 The *dksA* promoter is negatively feedback regulated by DksA and ppGpp. Mol Microbiol 80:1337–1348. doi:10.1111/j.1365-2958.2011.07649.x. 21496125 PMC3103630

[78] Margos G, Hepner S, Mang C, Marosevic D, Reynolds SE, Krebs S, Sing A, Derdakova M, Reiter MA, Fingerle V 2017 Lost in plasmids: next generation sequencing and the complex genome of the tick-borne pathogen *Borrelia burgdorferi*. BMC Genomics 18:422. doi:10.1186/s12864-017-3804-5. 28558786 PMC5450258

[79] Casjens SR, Mongodin EF, Qiu WG, Luft BJ, Schutzer SE, Gilcrease EB, Huang WM, Vujadinovic M, Aron JK, Vargas LC, Freeman S, Radune D, Weidman JF, Dimitrov GI, Khouri HM, Sosa JE, Halpin RA, Dunn JJ, Fraser CM 2012 Genome stability of Lyme disease spirochetes: comparative genomics of *Borrelia burgdorferi* plasmids. PLoS One 7:e33280. doi:10.1371/journal.pone.0033280. 22432010 PMC3303823

[80] Dowdell AS, Murphy MD, Azodi C, Swanson SK, Florens L, Chen S, Zuckert WR 2017 Comprehensive spatial analysis of the *Borrelia burgdorferi* lipoproteome reveals a compartmentalization bias toward the bacterial surface. J Bacteriol 199:e00658-16. 28069820 10.1128/JB.00658-16 PMC5331670

[81] Kenedy MR, Lenhart TR, Akins DR 2012 The role of *Borrelia burgdorferi* outer surface proteins. FEMS Immunol Med Microbiol 66:1–19. doi:10.1111/j.1574-695X.2012.00980.x. 22540535 PMC3424381

[82] Caine JA, Coburn J 2016 Multifunctional and redundant roles of *Borrelia burgdorferi* outer surface proteins in tissue adhesion, colonization, and complement evasion. Front Immunol 7:442. doi:10.3389/fimmu.2016.00442. 27818662 PMC5073149

[83] Drecktrah D, Hall LS, Hoon-Hanks LL, Samuels DS 2013 An inverted repeat in the *ospC* operator is required for induction in *Borrelia burgdorferi*. PLoS One 8:e68799. doi:10.1371/journal.pone.0068799. 23844242 PMC3700930

[84] Sze CW, Morado DR, Liu J, Charon NW, Xu H, Li C 2011 Carbon storage regulator A (CsrA_Bb_) is a repressor of Borrelia burgdorferi flagellin protein FlaB. Mol Microbiol 82:851–864. doi:10.1111/j.1365-2958.2011.07853.x. 21999436 PMC3212630

[85] Sanjuan E, Esteve-Gassent MD, Maruskova M, Seshu J 2009 Overexpression of CsrA (BB0184) alters the morphology and antigen profiles of Borrelia burgdorferi. Infect Immun 77:5149–5162. doi:10.1128/IAI.00673-09. 19737901 PMC2772508

[86] Sherlock ME, Sudarsan N, Breaker RR 2018 Riboswitches for the alarmone ppGpp expand the collection of RNA-based signaling systems. Proc Natl Acad Sci U S A 115:6052–6057. doi:10.1073/pnas.1720406115. 29784782 PMC6003355

[87] Caimano MJ, Dunham-Ems S, Allard AM, Cassera MB, Kenedy M, Radolf JD 2015 Cyclic di-GMP modulates gene expression in Lyme disease spirochetes at the tick-mammal interface to promote spirochete survival during the blood meal and tick-to-mammal transmission. Infect Immun 83:3043–3060. doi:10.1128/IAI.00315-15. 25987708 PMC4496621

[88] Rogers EA, Terekhova D, Zhang HM, Hovis KM, Schwartz I, Marconi RT 2009 Rrp1, a cyclic-di-GMP-producing response regulator, is an important regulator of *Borrelia burgdorferi* core cellular functions. Mol Microbiol 71:1551–1573. doi:10.1111/j.1365-2958.2009.06621.x. 19210621 PMC2843504

[89] Ouyang Z, Blevins JS, Norgard MV 2008 Transcriptional interplay among the regulators Rrp2, RpoN and RpoS in *Borrelia burgdorferi*. Microbiology 154:2641–2658. doi:10.1099/mic.0.2008/019992-0. 18757798

[90] Boardman BK, He M, Ouyang Z, Xu H, Pang X, Yang XF 2008 Essential role of the response regulator Rrp2 in the infectious cycle of *Borrelia burgdorferi*. Infect Immun 76:3844–3853. doi:10.1128/IAI.00467-08. 18573895 PMC2519420

[91] Rao NN, Liu S, Kornberg A 1998 Inorganic polyphosphate in *Escherichia coli*: the phosphate regulon and the stringent response. J Bacteriol 180:2186–2193. 9555903 10.1128/jb.180.8.2186-2193.1998 PMC107147

[92] Richards CL, Lawrence KA, Su H, Yang Y, Yang XF, Dulebohn DP, Gherardini FC 2015 Acetyl-phosphate is not a global regulatory bridge between virulence and central metabolism in *Borrelia burgdorferi*. PLoS One 10:e0144472. doi:10.1371/journal.pone.0144472. 26681317 PMC4683003

[93] Elias AF, Stewart PE, Grimm D, Caimano MJ, Eggers CH, Tilly K, Bono JL, Akins DR, Radolf JD, Schwan TG, Rosa P 2002 Clonal polymorphism of *Borrelia burgdorferi* strain B31 MI: implications for mutagenesis in an infectious strain background. Infect. Immun 70:2139–2150. doi:10.1128/IAI.70.4.2139-2150.2002. PMC127854 11895980

[94] Hughes CA, Kodner CB, Johnson RC 1992 DNA analysis of *Borrelia burgdorferi* NCH-1, the first northcentral U.S. human Lyme disease isolate. J Clin Microbiol 30:698–703. 1551988 10.1128/jcm.30.3.698-703.1992 PMC265135

[95] Xiang X, Yang Y, Du J, Lin T, Chen T, Yang XF, Lou Y 2017 Investigation of *ospC* expression variation among *Borrelia burgdorferi* strains. Front Cell Infect Microbiol 7:131. doi:10.3389/fcimb.2017.00131. 28473966 PMC5397415

[96] Bunikis I, Kutschan-Bunikis S, Bonde M, Bergström S 2011 Multiplex PCR as a tool for validating plasmid content of *Borrelia burgdorferi*. J Microbiol Methods 86:243–247. doi:10.1016/j.mimet.2011.05.004. 21605603

[97] Samuels DS, Drecktrah D, Hall LS 2018 Genetic transformation and complementation. Methods Mol Biol 1690:183–200. doi:10.1007/978-1-4939-7383-5_15. 29032546 PMC5806694

[98] Benjamini Y, Hochberg Y 1995 Controlling the false discovery rate: a practical and powerful approach to multiple testing. J R Stat Soc Series B Stat Methodol 57:289–300.

