# Supplementary material for: DksA Controls the Response of the Lyme Disease Spirochete Borrelia burgdorferi to Starvation
Source: J Bacteriol. 2019 Jan 28;201(4):e00582-18. doi: 10.1128/JB.00582-18 (PMC6351744; doi:10.1128/JB.00582-18)
Supplement: Supplemental file 1 [file 1c5fbe8dfa9d679e6dfea008908777ef_JB.00582-18-s0001.pdf]

Supplementary Material

Materials and methods

Genetic transformation

The *bb0168* mutant strain, Δ*dksA*, was generated in the *B. burgdorferi* B31-A3 background by homologous recombination. Homologous regions flanking the *bb0168* ORF (accession no. NP_212302.1) immediately upstream and downstream were amplified by PCR into DNA fragments. The upstream fragment was amplified using the TB-bb0168-F1-5′ primer containing BamHI restriction site (ACTGCTGGATCCAGTTATGGACTTCAAGGCTG) and the TB-bb0168-F1-3′ primer containing EcoRI restriction site (AACAGCTTTTTGCATGAATTCTCCCTATAAATTACA), creating a 495 bp fragment (F1). The downstream fragment was amplified using the TB-bb0168-F2-5′ primer containing the ClaI restriction site (GAAAAAAAGAACAAAAATCGATACATCTTAAGTTAGTTA) and the TB-bb0168-F2 3′ primer containing the XhoI restriction site (ACTGCTCTCGAGAACCAACATTTTAAAATGATTGG), creating a 499 bp fragment (F2). The homologous fragments were joined to a streptomycin resistance cassette, *aadA*, driven by *flgB* promoter. The F1 and F2 fragments were digested using EcoRI and ClaI restriction enzymes, respectively. The *aadA* expression cassette was amplified from pKFSS1 (Frank et al., 2003) using the primers *aadA*-5′-EcoRI (ACTGCTGAATTCTACCCGAGCTTCAAGGAA) and *aadA*-3′-ClaI (ACTGCTATCGATTATTTGCCGACTACCTTG), and then digested with both EcoRI and ClaI. Each DNA fragment was purified by PCR-purification kit (Thermo Fisher Scientific, Grand Island, NY, United States) and then ligated with T4 DNA ligase. The homologous recombination construct containing fragments F1, *flgBp-aadA*, and F2 was inserted into a pPCR-script plasmid with a subsequent round of restriction digestion with BamHI and XhoI, and ligation reaction. The resulting plasmid, pCm-Δ*dksA-aadA*, was electroporated into *B. burgdorferi* B31-A3 and *B. burgdorferi* were plated on pBSK with streptomycin for selection. PCR was used to assess the presence of the *dksA* gene, the *aadA* gene, and plasmid content in transformed *B. burgdorferi* (Blevins et al., 2008; Samuels et al., 2018).

A Δ*dksA* strain was also generated in the *B. burgdorferi* 297 strain (Hughes et al., 1992) background by homologous recombination. The upstream fragment (F1) was amplified to include an engineered AscI site at the 3′ end and produced a 982 bp product (AG-bb0168-F1-5′ CAGCGTAAATAAGCAAGGAGAATATACAATAGGAGC and AG-bb0168-F1-3′ GGCGCGCCGGCTTGTGAAAGAGAGATTGCTAGGGAG). The downstream fragment (F2) was amplified to include an engineered AscI site at the 5′ end and produced a 1,079 bp product (AG-bb0168-F2-5′ GGCGCGCCTCTTCAATAAACTCATGCTCAGAAACAGC and AG-bb0168-F2-3′ GCGCGCGATGCTGCACTTATCAAGATAGATAACATTC). The F1 and F2 fragments were individually TA cloned into pGEM-T Easy (Promega Corp., Madison, WI, United States) and confirmed by sequencing. The F2 fragment was excised from pGEM-T Easy using BssHII and ligated into the AscI-linearized F1 vector. A kanamycin cassette, *flgBp-aphI*-T7t (Groshong et al., 2012), was inserted between the F1 and F2 fragments via the engineered AscI site. The resulting mutagenesis construct (pJSB636A) was confirmed by sequencing and contains the kanamycin cassette in the forward orientation. pJSB636A was electroporated into *B. burgdorferi* 297 and plated with kanamycin for selection. Positive transformants were recovered and evaluated by PCR for kanamycin replacement of *bb0168* and for plasmid content (Blevins et al., 2008).

A *dksA trans* complementation vector, pDksA, was constructed in the pBSV2G (Elias et al., 2003) and pKFSS1 (Frank et al., 2003) backgrounds to restore the expression of DksA in the Δ*dksA* strains. The genomic DNA fragment containing the *dksA* gene and its 600 bp upstream sequence was amplified by PCR using primers *dksA* KpnI F and *dksA* FLG XhoI R. The amplification product retains the predicted native transcriptional start site for *dksA* and introduces a C-terminal DYKDDDDK(FLAG) epitope tag. The *dksA* amplicon was inserted in the background plasmid by restriction digest with KpnI and XhoI and ligation with T4 DNA ligase. Expression of *dksA* RNA and translated protein was detected by RT-qPCR and western blot, respectively.

Blevins, J.S., Hagman, K.E., and Norgard, M.V. (2008). Assessment of decorin-binding protein A to the infectivity of Borrelia burgdorferi in the murine models of needle and tick infection. *BMC Microbiol* 8**,** 82.

Elias, A.F., Bono, J.L., Kupko, J.J., 3rd, Stewart, P.E., Krum, J.G., and Rosa, P.A. (2003). New antibiotic resistance cassettes suitable for genetic studies in Borrelia burgdorferi. *J Mol Microbiol Biotechnol* 6**,** 29-40.

Elias, A.F., Stewart, P.E., Grimm, D., Caimano, M.J., Eggers, C.H., Tilly, K., Bono, J.L., Akins, D.R., Radolf, J.D., Schwan, T.G., and Rosa, P. (2002). Clonal polymorphism of Borrelia burgdorferi strain B31 MI: implications for mutagenesis in an infectious strain background. *Infect Immun* 70**,** 2139-2150.

Frank, K.L., Bundle, S.F., Kresge, M.E., Eggers, C.H., and Samuels, D.S. (2003). aadA confers streptomycin resistance in Borrelia burgdorferi. *J Bacteriol* 185**,** 6723-6727.

Groshong, A.M., Gibbons, N.E., Yang, X.F., and Blevins, J.S. (2012). Rrp2, a prokaryotic enhancer-like binding protein, is essential for viability of Borrelia burgdorferi. *J Bacteriol* 194**,** 3336-3342.

Hughes, C.A., Kodner, C.B., and Johnson, R.C. (1992). DNA analysis of Borrelia burgdorferi NCH-1, the first northcentral U.S. human Lyme disease isolate. *J Clin Microbiol* 30**,** 698-703.

Samuels, D.S., Drecktrah, D., and Hall, L.S. (2018). Genetic Transformation and Complementation. *Methods Mol Biol* 1690**,** 183-200.

```
Borrelia burgdorferi B31   MQKAVSEHEFIEEIKKFLSAEKREILDSIKSVENSKKEIINNDMYPKDVVDIAFDNMDGN   60
Borrelia garinii PBi       MQKASSEHEFIEEIKKFLSAEKREILDSIKSVENSKKEIINNDMYPKDVVDIAFDNMDGN   60
Borrelia afzelii PKo       MQKAGSEHEFIEEIKKFLSAEKKEILDSIKSVENSKKEIINNDMYPKDVVDIAFDNMDGN   60
Borrelia recurrentis A1    MQKSNFEHEFVEKMQTLLLELKKEILNSIRSVEDSKREIINNDMHLKDIVDIAFDNMDGN   60
Borrelia hermsii DAH       MQKSNFEHEFIEKMHNFLLESKKEILNSIRSVEDSKREIINNDMHLKDIIDIAFDNMDGN   60
Borrelia turicatae 91E135  MQKSNFEHEFVEKMRNFLLESKKEILNSIRSVEDSKREIINNDMHLKDIIDIAFDNMDGN   60
                           ***:  ****:*:::.:*   *:***:**:***:**:*******: **::**********

Borrelia burgdorferi B31   NLEALGFVEKRKLNLINQALYRISQNSYGKCLACEREIARERLLAIPYAFLCISCQTKKE   120
Borrelia garinii PBi       NLEALGFVEKRKLNLINQALYRISQNSYGKCLACEKEIARERLLAIPYAFLCISCQTKKE   120
Borrelia afzelii PKo       NLEALGFVEKRKLNLINQALYRISQNSYGKCLACEKEIARERLLAIPYAFLCISCQTKKE   120
Borrelia recurrentis A1    NLEALSSVEKKKLNLINQALYRISHNIYGHCLACDKDIAQERLEAIPYAFLCISCQTKKE   120
Borrelia hermsii DAH       NLEALSSVEKKKLHLINQALYRISQNTYGNCLACDKNIARERLEAIPYAFLCISCQTKKE   120
Borrelia turicatae 91E135  NLEALSSVEKKKLHLINQALYRISQNTYGNCLACDKSITRERLVAIPYAFLCISCQTKKE   120
                           *****. ***:**:**********:* **:****::.*::*** ****************

Borrelia burgdorferi B31   KKNKR--  125
Borrelia garinii PBi       KKSKR--  125
Borrelia afzelii PKo       KKSKR--  125
Borrelia recurrentis A1    KKSRRSI  127
Borrelia hermsii DAH       KKSRRSV  127
Borrelia turicatae 91E135  KKGKRSI  127
                           **.:*
```

Borrelia burgdorferi B31	100.0% sequence identity
Borrelia garinii PBi	97.6%
Borrelia afzelii PKo	96.8%
Borrelia recurrentis A1	73.6%
Borrelia hermsii DAH	75.2%
Borrelia turicatae 91E135	74.4%

Figure S1. Clustal Omega alignment indicates DksA amino acid sequence identity is high among *Borrelia*. The (*) indicates an amino acid residue conserved in all species, (:) indicates strong similarity, and (.) indicates weak similarity based on the PAM 250 matrix.

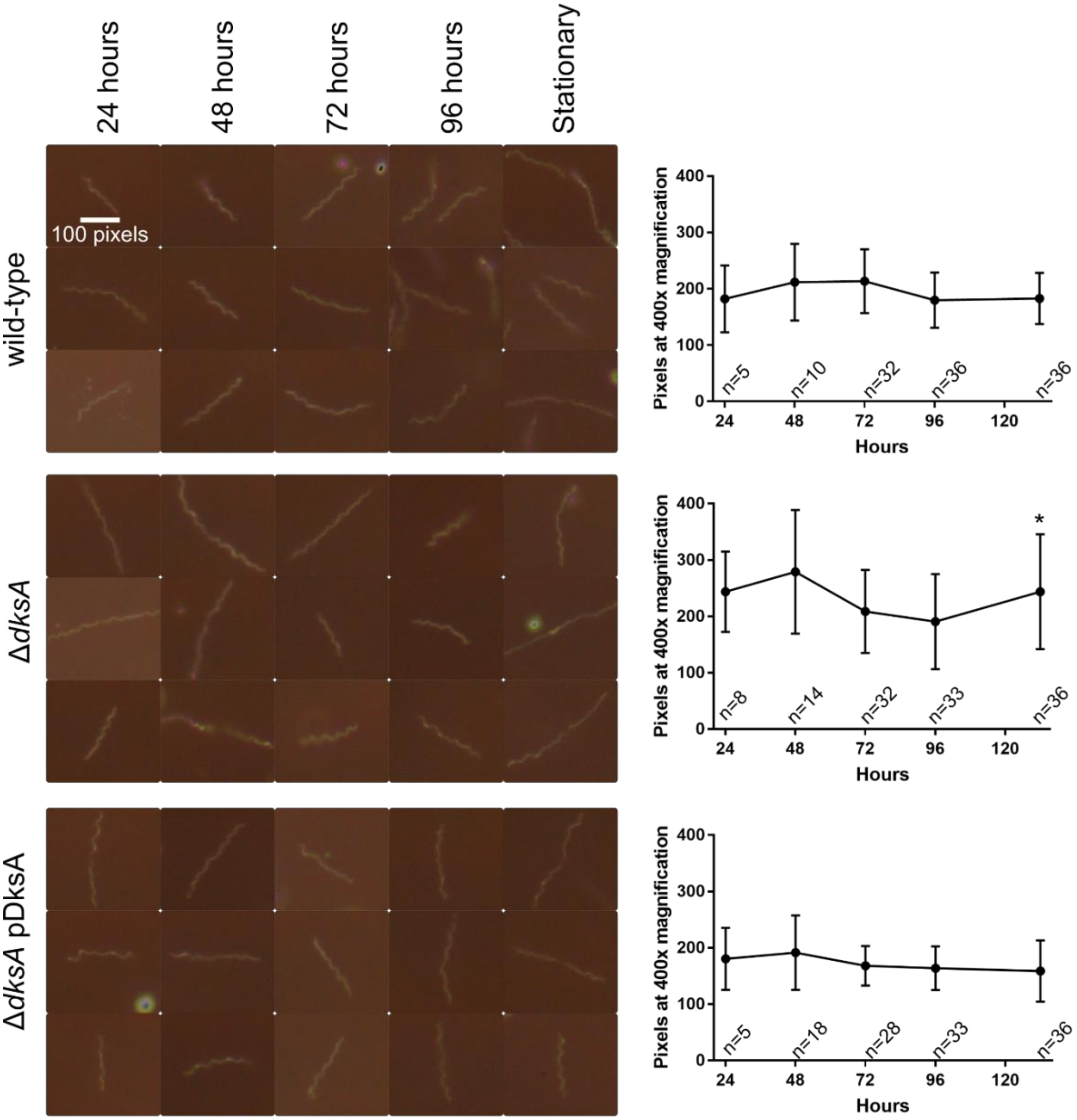

Figure S2. Dark field microscopy of wild-type, Δ*dksA*, and Δ*dksA* pDksA strains. *B. burgdorferi* cultures in BSK-II were passaged at a density of 1 x 10^5 spirochetes ml^{-1}, then cultures were wet mounted for imaging every 24 h. At 24 h, 48 h, and at stationary phase, elongated forms of Δ*dksA* strain are detectable. The relative lengths of spirochetes were determined by measuring pixel lengths in ImageJ. Error bars represent standard deviation. ANOVA with Tukey's post-hoc test was performed to compare the mean lengths of spirochetes between strains on each day. Asterisks represent statistical significance (p = 0.004).

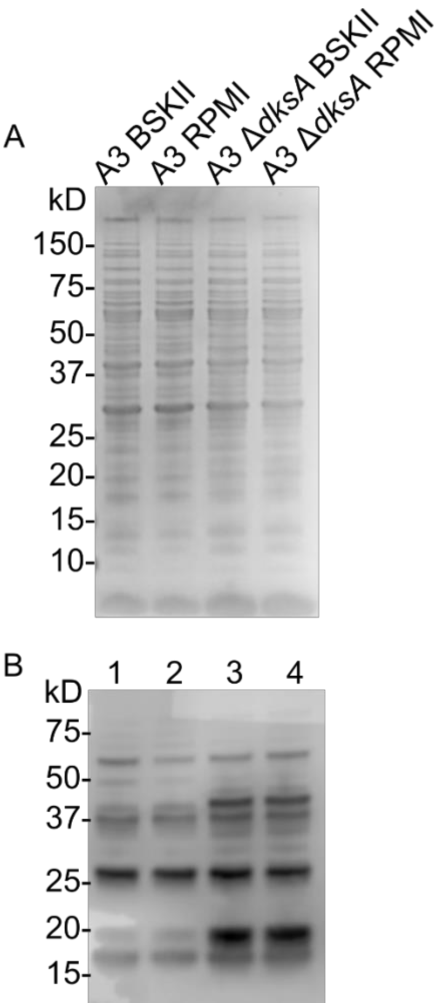

Figure S3. Protein expression of wild-type and Δ*dksA* spirochetes do not change significantly during starvation. (A) SDS-PAGE gel of lysates harvested from spirochetes at logarithmic growth at 5 x 10^7 spirochetes ml^{-1} in BSK-II and spirochetes following 6 h of starvation in RPMI. (B) A western blot was performed with protein lysates represented in (A) with serum from wild-type *B. burgdorferi*-infected mice. Lanes 1-4 in (B) correspond to respective lanes in (A).

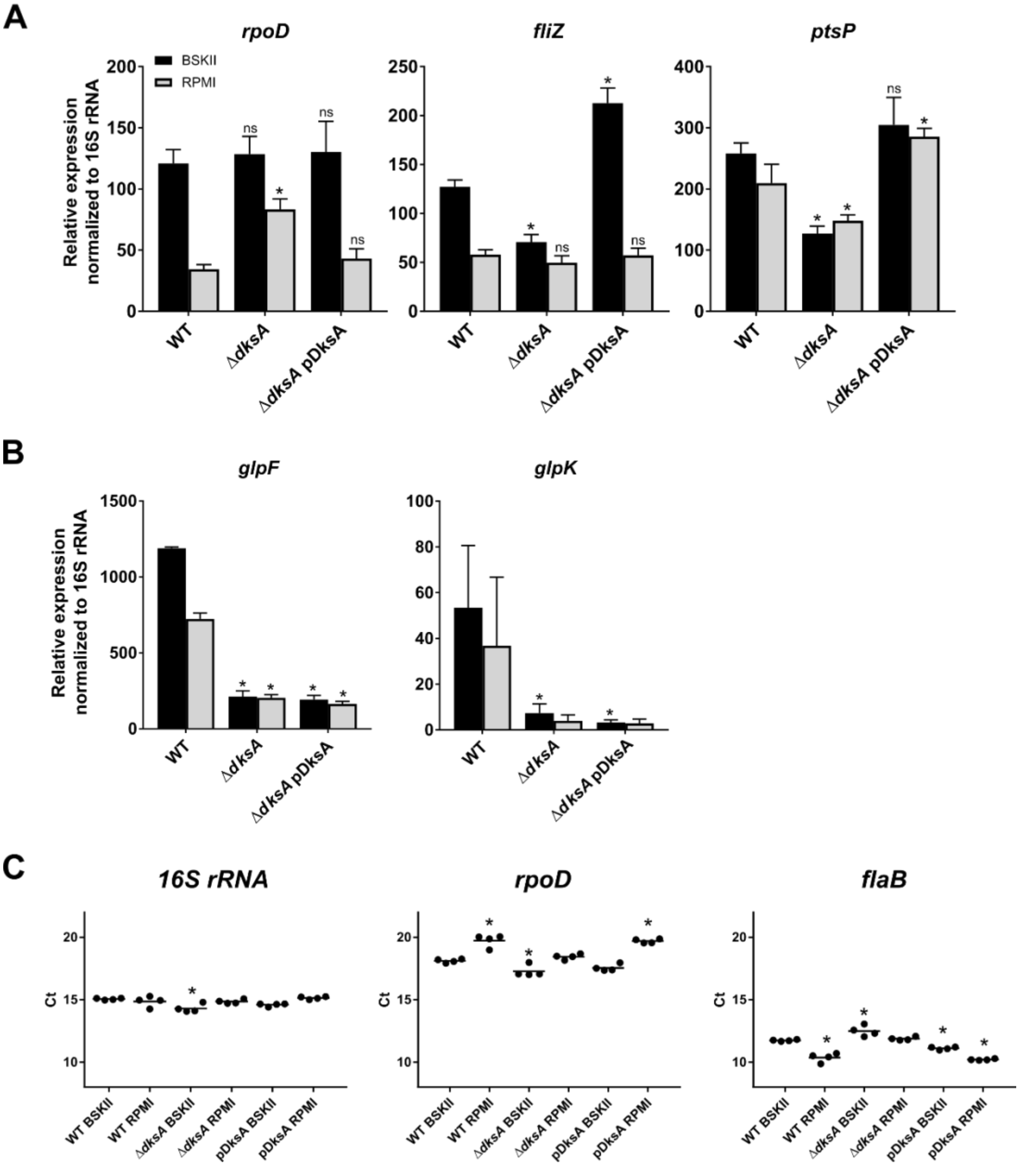

Figure S4. Expression of starvation-regulated genes among wild-type, Δ*dksA*, and Δ*dksA* pDksA strains. (A and B) RT-qPCR was performed on RNA extracted from wild-type (WT), Δ*dksA*, and Δ*dksA* pDksA mid-logarithmic phase cultures (black) and cultures starved in RPMI (gray). Error bars represent standard deviation calculated from four biological replicates. The Dunnett's multiple comparison test was performed between strains for BSK II and RPMI conditions. The asterisk indicates a p-value < 0.01 for expression level compared to the wild-type strain in respective BSK II or RPMI. (A) Relative changes in expression of *rpoD*, *fliZ*, and *ptsP* between logarithmic growth and starvation is restored in the Δ*dksA* pDksA. (B) While the expression of glycerol utilization genes (*glpF* and *glpK*) were reduced in the Δ*dksA* background,

the in *trans* complementation did not rescue the expression of these genes. (C) Reference gene RNA expression in response to starvation. The Ct value for a RT-qPCR with a normalized 10 ng RNA equivalent input of cDNA and primer sets for three commonly utilized reference genes, 16S rRNA, *rpoD*, and *flaB* are shown. The Dunnett's multiple comparison test was performed between WT BSK-II RNA against all other samples. The asterisk indicates p-value < 0.01 for Ct value. 16S rRNA provided least variable Ct values per ng RNA input.

Table S1. Genes expressed higher by DksA-deficient *B. burgdorferi* during logarithmic growth.

ID	Symbol	Description	Relative expression Δ*dksA* / WT	FDR adjusted P-value	Category	Δ*rel* $_{Bbu}$ / WT comparison at logarithmic or stationary phase (Bugrysheva, et al.)	Δ*rel* $_{Bbu}$/ WT comparison at stationary phase (Drecktrah, et al.)
BB_L01		phage portal protein	11.01	8.88E-09	Bacteriophage		
BB_A31		pbsx family phage terminase	5.60	0.0015452	Bacteriophage	2.94	
BB_N23	blyA	BlyA family holin	4.05	0.0224116	Bacteriophage	3.09	
BB_A38		phage portal protein	3.89	1.42E-07	Bacteriophage	3.1	7.31
BB_L23	blyA	BlyA family holin	3.64	0.0362139	Bacteriophage		
BB_L24	blyB	BlyB family holin	3.23	0.0001344	Bacteriophage		
BB_G21		putative phage terminase large subunit	2.95	0.0011066	Bacteriophage		
BB_H13		RepU	930.77	8.88E-09	Cell Division		
BB_H28		PF-32 Protein	34.37	1.29E-07	Cell Division		
BB_0301	ftsQ	cell division protein B	3.92	2.97E-07	Cell Division		
BB_H29		Plasmid partitioning protein	3.91	0.0096163	Cell Division		
BB_0431		CobQ/CobB/MinD/ParA nucleotide binding domain-containing protein	3.47	8.57E-05	Cell Division		
BB_F24		PF-32 Protein	3.40	2.24E-06	Cell Division		
BB_0472	murA	UDP-N-acetylglucosamine 1-carboxyvinyltransferase	3.22	1.16E-07	Cell Division		
BB_G08	Spo0J	stage 0 sprulation protein	2.87	1.60E-05	Cell Division		
BB_0302	ftsW	cell division protein FtsW	2.74	0.0096729	Cell Division		7.78
BB_J18		PF-32 Protein	2.09	0.0492359	Cell Division		
BB_0300	ftsA	cell division protein FtsA	2.02	0.0001561	Cell Division	4.03	

BB_H06	cspZ	complement regulator-acquiring surface protein	177.50	8.88E-09	Cell Envelope	
BB_H32		P35 antigen	86.69	6.30E-09	Cell Envelope	
BB_H37		lipoprotein	21.05	5.04E-07	Cell Envelope	
BB_A69		putative surface protein	10.72	1.71E-08	Cell Envelope	
BB_A57		P45-13	10.06	1.04E-05	Cell Envelope	
BB_0759		membrane protein	7.52	0.0100028	Cell Envelope	
BB_0117		hemolysin III	6.32	0.0012111	Cell Envelope	
BB_K50		immunogenic protein P37	6.08	1.02E-06	Cell Envelope	
BB_0365	la7	lipoprotein LA7	5.95	6.30E-09	Cell Envelope	
BB_0017		integral membrane protein	5.10	0.0014213	Cell Envelope	
BB_0584		integral membrane protein	4.69	2.53E-05	Cell Envelope	
BB_B27		lipoprotien	4.37	4.06E-06	Cell Envelope	
BB_J09	ospD	outer surface protein D	4.12	1.10E-06	Cell Envelope	
BB_M37	bppC	BppC Protein	3.97	0.0377093	Cell Envelope	
BB_0016	glpE	GlpE protein	3.96	1.06E-07	Cell Envelope	
BB_A68	cspA	complement regulator-acquiring surface protein 1	3.82	0.0001531	Cell Envelope	
BB_A03		outer membrane protein	3.59	1.15E-05	Cell Envelope	
BB_0473		Integral membrane protein	3.56	1.34E-05	Cell Envelope	
BB_K07		lipoprotein	3.51	1.38E-05	Cell Envelope	
BB_J34		lipoprotein	3.49	0.0001068	Cell Envelope	
BB_0583		integral membrane protein	2.78	1.40E-05	Cell Envelope	
BB_0628		lipoprotein	2.64	7.22E-05	Cell Envelope	
BB_0167		outer membrane protein	2.61	0.0143426	Cell Envelope	
BB_A52		outer membrane protein	2.52	0.0001037	Cell Envelope	
BB_R28	mlpD	mlpD	2.50	0.0037356	Cell Envelope	
BB_S41	erpG	ErpG protein	2.43	9.02E-05	Cell Envelope	
BB_0383	bmpA	basic membrane protein A	2.43	4.43E-06	Cell Envelope	
BB_0384	bmpC	basic membrane protein C	2.19	0.0003251	Cell Envelope	
BB_0616		Integral membrane protein	2.11	0.0099123	Cell Envelope	
BB_0158		antigen, S2	2.09	0.0011092	Cell Envelope	16.00
BB_N38	erpP	ErpP protein	2.07	0.0007647	Cell Envelope	

BB_0719	mrdB	rod shape-determining protein RodA	2.03	0.0004314	Cell Envelope		
BB_0726	ylxH-3	flagellar biosynthesis protein FlhG	2.83	4.50E-05	Chemotaxis and motility	3.87	3.05
BB_0578	mcp-1	methyl-accepting chemotaxis protein	2.34	2.74E-06	Chemotaxis and motility		8.34
BB_0288	fliI	flagellum-specific ATP synthase FliI	2.25	0.0003979	Chemotaxis and motility		
BB_0775	flhO	flagellar hook-basal body complex protein	2.17	2.79E-05	Chemotaxis and motility		2.04
BB_0046	rnhB	ribonuclease HII	10.75	0.0001379	DNA replication and repair		
BB_G32		replicative DNA helicase	4.61	1.10E-06	DNA replication and repair		
BB_0461		DNA polymerase III subunits gamma and tau	4.05	2.06E-08	DNA replication and repair		
BB_0829		exonuclease SbcD	3.56	0.0234384	DNA replication and repair		
BB_0053	ung	uracil-DNA glycosylase	3.52	0.0014391	DNA replication and repair		
BB_0552	ligA	NAD-dependent DNA ligase LigA	3.38	0.0068545	DNA replication and repair		6.23
BB_0836	uvrB	excinuclease ABC, B subunit (uvrB)	2.63	0.000235	DNA replication and repair		5.10
BB_0035	parC	DNA topoisomerase IV subunit A	2.57	0.0001746	DNA replication and repair		
BB_0623	mfd	transcription-repair coupling factor	2.07	0.0373433	DNA replication and repair		
BB_0036	parE	DNA topoisomerase IV subunit B	2.07	0.0003259	DNA replication and repair		
BB_0422		3-methyladenine DNA glycosylase	2.07	0.0001452	DNA replication and repair		
BB_H33		adenine deaminase	70.02	9.59E-06	Metabolism		
BB_0656	hemN	oxygen-independent coproporphyrinogen III oxidase	9.86	5.91E-08	Metabolism		
BB_0760	mazG	gene 37 protein (Gp37)	8.01	1.06E-07	Metabolism		
BB_0621		4-methyl-5(b-hydroxyethyl)-thiazole monophosphate biosynthesis protein	7.70	3.87E-12	Metabolism		
BB_0015	udk	uridine kinase	7.22	3.96E-09	Metabolism		4.53
BB_A76	thyX	FAD-dependent thymidylate synthase	6.86	1.37E-08	Metabolism		
BB_0644		N-acetylmannosamine-6-phosphate 2-epimerase	6.12	3.13E-07	Metabolism		
BB_0605		serine-type D-Ala-D-Ala carboxypeptidase	6.04	3.43E-08	Metabolism		
BB_0618	cdd	cytidine deaminase	5.60	0.0069376	Metabolism		
BB_0677	mglA	ribose/galactose ABC transporter, ATP-binding protein (mglA)	5.49	2.38E-07	Metabolism		5.74

BB_0408		PTS system, fructose-specific IIABC component (fruA-1)	5.16	1.02E-06	Metabolism	
BB_0645	ptsG	PTS system, glucose-specific IIBC component (ptsG)	4.84	3.82E-06	Metabolism	
BB_0793	tmk	thymidylate kinase	4.35	0.0004759	Metabolism	
BB_0109		acetyl-CoA C-acetyltransferase	4.30	3.11E-08	Metabolism	
BB_0515	trxB;	thioredoxin reductase	4.12	3.21E-08	Metabolism	
BB_K17		adenine deaminase	3.89	2.12E-05	Metabolism	
BB_0636	zwf	glucose-6-phosphate 1-dehydrogenase	3.39	4.71E-07	Metabolism	
BB_0679	mglC2	ribose/galactose ABC transporter, permease protein	3.36	2.45E-08	Metabolism	
BB_0407	manA	mannose-6-phosphate isomerase	3.30	1.57E-07	Metabolism	
BB_0635	pncB	nicotinate phosphoribosyltransferase	3.23	0.0017069	Metabolism	
BB_0421		haloacid dehalogenase-like hydrolase	3.08	2.78E-07	Metabolism	4.59
BB_0084		cysteine desulfurase	3.07	0.0004776	Metabolism	5.58
BB_0625		N-acetylmuramoyl-L-alanine amidase	2.54	6.83E-08	Metabolism	
BB_0364	mgsA	methylglyoxal synthase	2.53	0.0007647	Metabolism	
BB_0037		1-acyl-sn-glycerol-3-phosphate acyltransferase	2.45	0.0003096	Metabolism	
BB_0725		serine-type D-Ala-D-Ala carboxypeptidase	2.43	0.0301233	Metabolism	
BB_0601	glyA	serine hydroxymethyltransferase	2.39	2.71E-06	Metabolism	
BB_0561	gnd	6-phosphogluconate dehydrogenase	2.23	2.00E-06	Metabolism	
BB_0303	mraY	phospho-N-acetylmuramoyl-pentapeptide- transferase	2.14	7.65E-05	Metabolism	
BB_0709		aminodeoxychorismate lyase, putative	2.09	0.0039143	Metabolism	2.06
BB_0761	nlpD	peptidoglycan-binding protein	9.82	5.59E-05	Protein degradation	
BB_0369	clpA	ATP-dependent Clp protease, subunit A	5.00	0.001243	Protein degradation	
BB_0757	clpP	Clp protease	3.22	6.89E-08	Protein degradation	
BB_0185		glycoprotease family protein	2.41	0.0106663	Protein degradation	
BB_H18		pseudogene	80.66	5.49E-07	Pseudogene	
BB_L05		pseudogene	13.78	6.29E-09	Pseudogene	

BB_F13		pseudogene	9.28	0.0030244	Pseudogene		
BB_J10		pseudogene	7.74	1.93E-05	Pseudogene		
BB_J20		pseudogene	7.59	0.031771	Pseudogene		
BB_A55		pseudogene	7.31	8.53E-07	Pseudogene		
BB_F05		pseudogene	7.16	0.0333887	Pseudogene		
BB_K02		pseudogene	6.53	0.0013042	Pseudogene		
BB_F32		pseudogene	6.19	3.96E-09	Pseudogene	3.09	
BB_A50		pseudogene	5.75	0.0154108	Pseudogene		
BB_J15		pseudogene	5.41	0.0007437	Pseudogene		
BB_A39		pseudogene	5.32	0.0045571	Pseudogene		2.39
BB_F22		pseudogene	5.24	2.63E-06	Pseudogene		
BB_A14		pseudogene	5.05	0.0069506	Pseudogene		7.36
BB_F16		pseudogene	4.33	0.0001763	Pseudogene		
BB_0845		pseudogene	3.95	0.0014541	Pseudogene		
BB_A56		pseudogene	3.42	2.44E-05	Pseudogene		
BB_A22		pseudogene	3.18	0.0006357	Pseudogene		3.34
BB_M22		pseudogene	3.14	0.0007859	Pseudogene		
BB_G06		pseudogene	2.27	1.05E-05	Pseudogene		
BB_0519	grpE	GrpE protein	3.88	8.08E-06	Stress Response		
BB_0518	dnaK	molecular chaperone DnaK	2.72	4.44E-07	Stress Response	2.34	4.14
BB_0264	Hsp70	heat shock protein 70	2.31	4.17E-05	Stress Response		
BB_0517	dnaJ	chaperone protein DnaJ	2.18	0.0015527	Stress Response	2.84	
BB_D18		hypothetical protein (rpoS repression)	5.98	1.10E-06	Transcription and regulation		
BB_0781	obg	GTPase Obg	2.96	1.02E-08	Transcription and regulation	4.98	3.86
BB_0693	badR	xylose operon regulatory protein	2.35	2.88E-06	Transcription and regulation		
BB_0427		rRNA small subunit methyltransferase I	10.91	1.16E-07	Translation		
BB_0263	lepB	signal peptidase I	7.16	0.0001174	Translation		
BB_0590	ksgA	KsgA/Dim1 family 16S ribosomal RNA methyltransferase	4.56	0.0099215	Translation		
BB_0135	hisS	histidyl-tRNA synthetase	4.55	4.44E-07	Translation		
BB_0682	trmU	tRNA-specific 2-thiouridylase MnmA	4.41	0.0023755	Translation		
BB_0214	efp	elongation factor P	4.22	3.38E-07	Translation		
BB_0788	tilS	tRNA(Ile)-lysidine synthase	3.91	0.0019798	Translation		2.25

BB_0734		Sua5/YciO/YrdC/YwlC family protein	3.19	0.0119751	Translation		
BB_0787	pth	peptidyl-tRNA hydrolase	2.96	2.08E-06	Translation		
BB_0143		membrane protein insertion efficiency factor	2.71	1.72E-05	Translation		
BB_0169	infA	translation initiation factor IF-1	2.65	0.0023389	Translation		
BB_0483	rplV	50S ribosomal protein L22	2.59	2.71E-06	Translation	2.94	
BB_0780	rpmA	50S ribosomal protein L27	2.55	0.0020275	Translation	2.89	2.04
BB_0659	lysS	lysyl-tRNA synthetase	2.41	0.000947	Translation		
BB_0484	rpsC	30S ribosomal protein S3	2.29	0.0001353	Translation	2.78	
BB_0482	rpsS	30S ribosomal protein S19	2.17	0.0001577	Translation	2.51	
BB_0256	rpsU	30S ribosomal protein S21	2.14	4.42E-05	Translation		
BB_0660	era	GTPase Era	2.11	0.0003595	Translation		
BB_0481	rplB	50S ribosomal protein L2	2.10	4.77E-06	Translation	2.18	
BB_0186	tsaE	tRNA threonylcarbamoyladenosine biosynthesis protein	2.02	0.022608	Translation		
BB_B23	pbuG	guanine/xanthine permease	6.92	3.16E-06	Transporter Proteins		7.94
BB_A74	oms28	outer membrane porin	5.83	2.07E-08	Transporter Proteins		
BB_0453		small conductance mechanosensitive ion channel	4.61	1.93E-05	Transporter Proteins		
BB_0641	potH	putrescine transport system permease protein PotH	4.58	0.0166154	Transporter Proteins	2.5	
BB_B05	chbA	chitobiose transporter protein	4.42	0.0002703	Transporter Proteins		16.56
BB_0216	pstC	phosphate ABC transporter permease	4.25	0.0010279	Transporter Proteins		2.64
BB_0724		potassium transporter	4.16	1.00E-08	Transporter Proteins		2.69
BB_0642	potA	spermidine/putrescine ABC transporter ATP-binding protein	3.85	0.0020046	Transporter Proteins		53.08
BB_0447		Na+/H+ antiporter (putative)	3.66	8.83E-07	Transporter Proteins		
BB_0452		chromate transporter superfamily	3.63	0.0195114	Transporter Proteins		
BB_0678	mglC1	sugar ABC transporter permease	2.73	0.0003444	Transporter Proteins		
BB_B06	chbB	chitobiose transporter protein	2.28	0.0190565	Transporter Proteins		32.45
BB_0451		chromate transport protein	2.17	0.0169344	Transporter Proteins		
BB_0448	hpr	phosphocarrier protein	2.04	0.0074269	Transporter Proteins		
BB_H26		hypothetical protein	335.70	1.88E-07	Unknown		

BB_H35	hypothetical protein	62.59	1.23E-06	Unknown		
BB_H31	hypothetical protein	49.83	4.24E-06	Unknown		
BB_F14	hypothetical protein	46.63	0.000176	Unknown		
BB_H25	hypothetical protein	26.80	1.17E-05	Unknown		
BB_L06	hypothetical protein	18.73	4.08E-05	Unknown		
BB_M08	hypothetical protein	18.02	0.0013665	Unknown		8.22
BB_K30	hypothetical protein	17.99	0.0001353	Unknown		
BB_G15	hypothetical protein	16.51	4.21E-05	Unknown	3.42	
BB_L07	hypothetical protein	13.82	2.38E-07	Unknown		
BB_A23	hypothetical protein	13.35	6.85E-07	Unknown	2.43	3.05
BB_L03	hypothetical protein	13.16	4.93E-06	Unknown		
BB_H19	hypothetical protein	12.80	0.0037313	Unknown		
BB_H18	hypothetical protein	12.20	0.0004197	Unknown		
BB_K11	hypothetical protein	11.84	0.0005807	Unknown		5.46
BB_L04	hypothetical protein	11.28	2.37E-06	Unknown		
BB_D16	hypothetical protein	10.72	0.0019452	Unknown		
BB_O21	hypothetical protein	10.66	0.0009617	Unknown	3.93	10.41
BB_M06	hypothetical protein	10.25	0.000275	Unknown		
BB_M03	hypothetical protein	9.45	0.0003782	Unknown		
BB_G24	hypothetical protein	9.36	3.04E-06	Unknown		
BB_N11	hypothetical protein	9.20	0.0035848	Unknown		
BB_L09	hypothetical protein	8.46	0.0001775	Unknown		
BB_M09	hypothetical protein	8.14	0.0009309	Unknown		
BB_H27	hypothetical protein	8.10	0.0039803	Unknown		
BB_M05	hypothetical protein	7.83	0.0006836	Unknown		
BB_G11	hypothetical protein	7.58	0.0096934	Unknown		
BB_J13	hypothetical protein	7.51	0.0002043	Unknown		
BB_F26	hypothetical protein	7.07	1.05E-05	Unknown		
BB_K31	hypothetical protein	6.92	0.0127393	Unknown		
BB_D12	hypothetical protein	6.66	2.74E-06	Unknown		
BB_F02	hypothetical protein	6.63	0.0394181	Unknown		
BB_G29	hypothetical protein	6.22	0.0001562	Unknown		
BB_K43	hypothetical protein	6.13	0.0054348	Unknown		

BB_F08	hypothetical protein	5.96	0.0223915	Unknown		
BB_M07	hypothetical protein	5.80	7.73E-05	Unknown		
BB_A51	hypothetical protein	5.61	0.0027271	Unknown	5.6	
BB_B10	hypothetical protein	5.50	2.59E-05	Unknown		
BB_A40	hypothetical protein	5.43	3.11E-08	Unknown	5.23	
BB_K18	hypothetical protein	5.37	0.0018844	Unknown		
BB_R31	hypothetical protein	5.34	1.89E-06	Unknown	2.69	
BB_B24	hypothetical protein	5.29	4.78E-06	Unknown		
BB_A47	hypothetical protein	5.27	0.0122292	Unknown	4.65	
BB_J11	hypothetical protein	5.26	0.0001775	Unknown		
BB_G30	hypothetical protein	5.26	0.0009201	Unknown		
BB_F22	hypothetical protein	5.24	2.63E-06	Unknown		
BB_A48	hypothetical protein	5.21	0.0072997	Unknown	4.52	
BB_J37	hypothetical protein	5.08	0.0296013	Unknown		
BB_A46	hypothetical protein	4.93	0.000675	Unknown	4.55	
BB_K42	hypothetical protein	4.93	0.0099566	Unknown		
BB_L13	hypothetical protein	4.91	0.0009559	Unknown		
BB_A09	hypothetical protein	4.76	0.0015748	Unknown	2.05	34.78
BB_L14	hypothetical protein	4.73	0.0113177	Unknown		
BB_A35	hypothetical protein	4.61	0.002625	Unknown		
BB_A10	hypothetical protein	4.53	0.000405	Unknown	2.24	
BB_A61	hypothetical protein	4.52	5.01E-06	Unknown		
BB_A30	hypothetical protein	4.46	1.29E-07	Unknown		
BB_A08	hypothetical protein	4.45	0.0116609	Unknown		
BB_L15	hypothetical protein	4.38	0.0062498	Unknown		
BB_B14	hypothetical protein	4.32	5.29E-05	Unknown		
BB_A11	hypothetical protein	4.26	0.0297429	Unknown	2.94	12.04
BB_0162	hypothetical protein	4.26	3.81E-07	Unknown		
BB_G25	hypothetical protein	4.16	0.0188209	Unknown		
BB_K49	hypothetical protein	4.15	9.31E-06	Unknown		
BB_G20	hypothetical protein	4.14	0.0113876	Unknown		
BB_0206	methyltransferase	4.05	5.49E-07	Unknown		
BB_G13	hypothetical protein	4.03	0.0189226	Unknown		

BB_O17	hypothetical protein	3.91	0.0016975	Unknown		
BB_G31	hypothetical protein	3.80	0.0014444	Unknown		
BB_S43	hypothetical protein	3.68	0.0333284	Unknown		
BB_B25	hypothetical protein	3.59	0.0008719	Unknown		
BB_H17	hypothetical protein	3.55	0.0042122	Unknown		
BB_O30	hypothetical protein	3.47	2.06E-08	Unknown		
BB_M10	hypothetical protein	3.34	0.0145176	Unknown		
BB_0050	hypothetical protein	3.30	0.0062498	Unknown		
BB_R29	hypothetical protein	3.26	0.0002024	Unknown	2.35	7.11
BB_A41	hypothetical protein	3.26	1.85E-05	Unknown	4.53	
BB_0525	hypothetical protein	3.26	4.94E-06	Unknown		5.54
BB_N02	hypothetical protein	3.21	0.0113876	Unknown	11.8	
BB_A13	hypothetical protein	3.21	0.0012265	Unknown	4.76	7.89
BB_K05	hypothetical protein	3.14	0.0443233	Unknown		
BB_J38	hypothetical protein	3.06	0.0008719	Unknown		
BB_S44	hypothetical protein	3.05	0.0333887	Unknown		
BB_K47	hypothetical protein	3.03	1.72E-05	Unknown		
BB_A53	Bbs27 protein	2.90	0.0012467	Unknown	2.33	
BB_0733	hypothetical protein	2.88	4.54E-05	Unknown		
BB_J19	hypothetical protein	2.86	3.97E-05	Unknown		
BB_A54	hypothetical protein	2.81	0.0008587	Unknown		
BB_0134	hypothetical protein	2.79	0.0443233	Unknown		
BB_O29	hypothetical protein	2.76	4.75E-05	Unknown		5.82
BB_Q29	hypothetical protein	2.73	0.0001965	Unknown		
BB_G14	hypothetical protein	2.72	0.0373439	Unknown		
BB_L22	hypothetical protein	2.67	0.0003895	Unknown		
BB_0592	hypothetical protein	2.64	0.0004495	Unknown		
BB_N14	hypothetical protein	2.57	0.0066743	Unknown		
BB_B02	hypothetical protein	2.49	0.0030244	Unknown		3.76
BB_M17	hypothetical protein	2.45	0.0461417	Unknown		
BB_0748	hypothetical protein	2.42	0.0166998	Unknown		8.82
BB_F25	hypothetical protein	2.34	4.20E-06	Unknown		
BB_0207	hypothetical protein	2.22	0.0258281	Unknown		

BB_M30	hypothetical protein	2.20	0.0004818	Unknown	
BB_K23	hypothetical protein	2.20	0.0044889	Unknown	
BB_J48	hypothetical protein	2.19	0.0001117	Unknown	
BB_0165	hypothetical protein	2.15	2.11E-05	Unknown	
BB_G19	hypothetical protein	2.13	0.0033304	Unknown	
BB_0526	hypothetical protein	2.06	0.000514	Unknown	
BB_G33	hypothetical protein	2.03	0.0125006	Unknown	3.76
BB_G18	hypothetical protein	2.00	0.0121709	Unknown	6.4

Table S2. Gene expressed higher by wild-type *B. burgdorferi* during logarithmic growth.

ID	Symbol	Description	Relative expression Δ*dksA* / WT	FDR adjusted P-value	Category	Δ*rel* Bbu / WT comparison at logarithmic or stationary phase (Bugrysheva, et al.)	Δ*rel* Bbu/ WT comparison at stationary phase (Drecktrah, et al.)
BB_Q40		PF-32 Protein	-19.94	4.08E-05	Cell Division		
BB_O33		Plasmid partitioning protein	-2.95	0.0297429	Cell Division	-2.23	-9.32
BB_A20		PF-32 Protein	-2.89	0.0167075	Cell Division		-4.26
BB_0785		regulatory protein SpoVG	-2.77	4.22E-06	Cell Division		
BB_0437	dnaA	protein	-2.18	0.0008586	Cell Division		
BB_P33		PF-32 Protein	-2.02	0.0033179	Cell Division		
BB_A64	p35	antigen P35	-66.91	2.49E-07	Cell Envelope		
BB_Q06		membrane protein	-38.19	2.16E-06	Cell Envelope	-2.95	
BB_B19	ospC	outer surface protein C	-21.98	2.82E-10	Cell Envelope		
BB_A65		lipoprotein	-15.95	1.40E-05	Cell Envelope		
BB_Q03		outer membrane protein	-14.85	7.89E-05	Cell Envelope		
BB_A73	P35	antigen P35	-13.39	5.87E-08	Cell Envelope	-2.24	
BB_A07	chpAI	ChpAI protein	-12.60	1.85E-07	Cell Envelope		
BB_A25	dbpB	decorin-binding protein B	-12.47	3.07E-09	Cell Envelope		
BB_A24	dbpA	decorin-binding protein A	-8.64	1.48E-06	Cell Envelope		
BB_Q05	P35	antigen P35	-6.35	0.0389522	Cell Envelope	-2.92	
BB_A66		outersurface protein	-5.78	0.0007865	Cell Envelope		
BB_Q35	mlpJ	multicopy lipoprotein J	-5.36	0.0014814	Cell Envelope	-2.74	
BB_A05	S1	S1 Antigen	-4.95	0.012313	Cell Envelope		
BB_0250		inner membrane protein	-4.81	0.0126331	Cell Envelope		
BB_0071		membrane protein	-4.49	4.89E-05	Cell Envelope		

BB_A15	ospA	outer surface protein A	-4.31	1.50E-07	Cell Envelope	-2.07	-3.51
BB_A16	ospB	outer surface protein B	-4.17	6.21E-07	Cell Envelope		-2.91
BB_0385	bmpD	basic membrane protein D	-4.11	5.19E-09	Cell Envelope		-7.62
BB_M34	bdrK	BdrK protien	-3.11	0.0098305	Cell Envelope		
BB_A36		lipoprotein	-3.06	1.94E-06	Cell Envelope		
BB_0674		membrane protein	-3.05	0.0215104	Cell Envelope		
BB_0603	p66	membrane-associated protein p66	-2.98	2.85E-06	Cell Envelope		
BB_0155		lipoprotein	-2.95	0.001775	Cell Envelope		
BB_Q42	bdrV	BdrV protien	-2.86	0.0001116	Cell Envelope		
BB_O39	erpL	ErpL protein	-2.83	4.63E-06	Cell Envelope		
BB_O40	erpM	ErMG protein	-2.60	6.13E-05	Cell Envelope	-2.2	
BB_L35		BdrO protein	-2.58	1.40E-05	Cell Envelope		
BB_F20		lipoprotein	-2.53	0.0003296	Cell Envelope	-3.05	-3.58
BB_0795	bamA	outer membrane protein	-2.27	0.0001982	Cell Envelope		
BB_M38	erpK/osp	ErpK/ospF protein	-2.14	0.0004823	Cell Envelope	-2.13	
BB_A62	lp6.6	lipoprotein	-2.11	0.0003259	Cell Envelope	-2.9	-5.98
BB_0353		membrane protein	-2.05	0.001276	Cell Envelope		
BB_0034		outer membrane protein P13	-2.03	0.0008068	Cell Envelope		
BB_0744	p83/100	antigen, p83/100	-2.01	0.0001982	Cell Envelope		
BB_0597	mcp3	methyl-accepting chemotaxis protein	-3.89	2.09E-06	Chemotaxis and motility		
BB_0183	fliW	flagellar assembly protein FliW	-3.72	6.86E-05	Chemotaxis and motility	-2.61	
BB_0182	flgL	flagellar hook-associated protein	-3.69	9.24E-07	Chemotaxis and motility		
BB_0596		methyl-accepting chemotaxis protein	-3.58	0.0001068	Chemotaxis and motility		
BB_0147	flaB	flagellar filament 41 kDa core protein	-2.58	0.0003264	Chemotaxis and motility		-2.48
BB_0570	cheY	chemotaxis protein CheY	-2.32	0.0001787	Chemotaxis and motility		
BB_0681	mcp-5	methyl-accepting chemotaxis protein	-2.13	0.0005452	Chemotaxis and motility	-3.74	
BB_0286		flagellar protein	-2.06	0.0120597	Chemotaxis and motility		
BB_0270	flhF	flagellar biosynthesis regulator FlhF	-2.00	7.49E-05	Chemotaxis and motility		
BB_Q67		adenine specific DNA methyltransferase	-8.82	0.0145176	DNA replication and repair	-2	
BB_0014	priA	primosomal protein N	-3.39	1.07E-05	DNA replication and repair	-2.17	
BB_0438	dnaN	DNA polymerase III subunit beta	-2.82	8.74E-06	DNA replication and repair		

BB_0254	recJ	single-stranded-DNA-specific exonuclease	-2.63	0.0270294	DNA replication and repair		
BB_0457	uvrC	excinuclease ABC subunit C	-2.62	0.0307362	DNA replication and repair	-2.13	
BB_0344	uvrD	DNA helicase	-2.53	0.0005163	DNA replication and repair		
BB_0827	hrpA	ATP-dependent helicase	-2.36	0.0009091	DNA replication and repair		
BB_0241	glpK	glycerol kinase	-23.09	2.11E-09	Metabolism		-44.32
BB_0243	glpD	glycerol-3-phosphate dehydrogenase	-14.05	1.84E-07	Metabolism		-13.83
BB_0248	pepF	oligoendopeptidase F	-6.88	0.0024371	Metabolism		
BB_0348	pyk	pyruvate kinase	-5.06	9.44E-07	Metabolism		-9.92
BB_0588	bgp	MTA/SAH nucleosidase	-4.23	7.06E-07	Metabolism		
BB_0249		phosphatidylcholine synthase	-4.05	0.0090386	Metabolism		
BB_0238	lnt	apolipoprotein N-acyltransferase	-3.90	3.46E-08	Metabolism		
BB_0152	nagB	glucosamine-6-phosphate deaminase	-3.45	0.0023125	Metabolism		
BB_0763	atoC	acetoacetate metabolism regulatory protein AtoC	-3.22	0.000286	Metabolism		
BB_0010	acpS	holo-acyl-carrier protein synthase	-3.00	9.38E-05	Metabolism	-2.93	-4.17
BB_0020	pfp	diphosphate--fructose-6-phosphate 1-phosphotransferase	-2.97	4.30E-08	Metabolism		-2.27
BB_0819		cytidylate kinase	-2.59	7.65E-06	Metabolism		
BB_0337	eno	enolase	-2.40	3.33E-07	Metabolism		-2.36
BB_0086		Mg chelatase-like protein	-2.21	0.0149923	Metabolism		
BB_0120	uppS	undecaprenyl pyrophosphate synthase	-2.00	0.0019362	Metabolism		
BB_0536	pqqL	zinc protease	-6.01	4.44E-07	Protein degradation	-2.43	-4.63
BB_0627	apeB/pep	aminopeptidase	-5.12	0.0013199	Protein degradation		
BB_0366	yscI	aminopeptidase I	-3.40	5.07E-06	Protein degradation		-4.53
BB_0253	lon	ATP-dependent protease LA	-2.86	3.11E-08	Protein degradation		
BB_Q41		pseudogene	-78.88	8.06E-08	Pseudogene		
BB_A71		pseudogene	-17.72	0.0001105	Pseudogene		
BB_0560	htpG	chaperone protein HtpG	-3.84	5.65E-06	Stress Response		
BB_0690	napA	neutrophil activating protein A (napA)	-3.32	2.45E-06	Stress Response	-3.02	
BB_0649	groL	chaperonin GroEL	-2.48	3.97E-06	Stress Response		
BB_0168	dksA	dnaK suppressor	-88.12	4.68E-05	Transcription and regulation		
BB_0626	rnmV	ribonuclease M5	-10.48	7.61E-05	Transcription and regulation		

BB_0198	rel	guanosine-3',5'-bis(diphosphate) 3'-pyrophosphohydrolase	-7.46	0.0002795	Transcription and regulation		
BB_0502	rpoA	DNA-directed RNA polymerase subunit alpha	-3.52	3.11E-08	Transcription and regulation		
BB_0820	rpoZ	DNA-directed RNA polymerase subunit omega	-2.47	0.0003972	Transcription and regulation		
BB_0033	smpB	SsrA-binding protein	-6.74	0.0025972	Translation	-3.79	
BB_0012	truA	tRNA pseudouridine synthase A	-6.28	0.0022215	Translation		
BB_0594	argS	arginyl-tRNA synthetase	-4.10	3.17E-07	Translation		
BB_0203	hflK	membrane protease subunit HflK	-3.79	0.0001094	Translation		-2.89
BB_0204	hflC	HflC protein	-3.27	0.0001146	Translation		
BB_0346		export chaperone	-2.79	0.0011614	Translation		-2.10
BB_0112	rplI	50S ribosomal protein L9	-2.74	2.21E-06	Translation		
BB_0503	rplQ	50S ribosomal protein L17	-2.58	1.26E-05	Translation		
BB_0122	tsf	elongation factor Ts	-2.55	2.07E-08	Translation		
BB_0127		30S ribosomal protein S1	-2.51	3.26E-06	Translation		
BB_0653	secF	preprotein translocase subunit SecF	-2.50	3.90E-05	Translation		
BB_0501	rpsK	30S ribosomal protein S11	-2.34	5.93E-05	Translation		
BB_0691	fusA	elongation factor G	-2.29	7.06E-07	Translation		
BB_0088	lepA	elongation factor EF-4	-2.21	4.52E-06	Translation		
BB_0123	rpsB	30S ribosomal protein S2	-2.17	3.18E-06	Translation		
BB_0101	asnS	asparaginyl-tRNA synthetase	-2.11	1.06E-05	Translation		
BB_0121	frr	ribosome recycling factor	-2.11	0.0005268	Translation		
BB_0358		16S ribosomal RNA methyltransferase	-2.06	0.0003545	Translation		
BB_0695	rpsP	30S ribosomal protein S16	-2.05	1.89E-05	Translation		
BB_0240	glpF	glycerol uptake facilitator	-9.02	6.31E-10	Transporter Proteins	-2.05	-27.10
BB_0401		glutamate transporter	-8.13	2.35E-06	Transporter Proteins	-2.63	
BB_0329	oppA-2	oligopeptide permease peptide binding protein	-7.41	2.07E-08	Transporter Proteins	-2.31	-2.51
BB_0454		lipopolysaccharide biosynthesis-like protein	-5.09	1.33E-08	Transporter Proteins		
BB_0328	oppA-1	oligopeptide permease peptide binding protein	-4.96	3.07E-09	Transporter Proteins	-2.29	-6.28

BB_0144		glycine/betaine ABC transporter substrate-binding protein	-2.52	9.23E-05	Transporter Proteins		
BB_0559	ccr	PTS system glucose-specific transporter	-2.21	2.71E-06	Transporter Proteins		
BB_0318		methylgalactoside ABC transporter ATP-binding protein	-2.00	0.0010265	Transporter Proteins		
BB_B22	pbuG1	guanine/xanthine permease	-2.00	0.0001901	Transporter Proteins		
BB_0242		hypothetical protein	-29.70	4.76E-06	Unknown	-2.64	
BB_Q49		hypothetical protein	-28.65	0.0003814	Unknown		
BB_Q78		hypothetical protein	-22.38	0.000675	Unknown		
BB_D24		hypothetical protein	-18.45	0.0001181	Unknown	-2.39	
BB_A72		hypothetical protein	-16.43	8.88E-09	Unknown		
BB_A37		hypothetical protein	-9.19	0.0003814	Unknown	-4.18	
BB_J01		hypothetical protein	-8.16	0.0164636	Unknown		
BB_0013		hypothetical protein	-6.91	0.0082159	Unknown	-3.58	
BB_A06		hypothetical protein	-6.71	0.0188698	Unknown		
BB_0212		hypothetical protein	-6.54	0.0166631	Unknown	-2.94	
BB_0011		hypothetical protein	-5.78	0.0008974	Unknown		
BB_0367		hypothetical protein	-5.65	0.0008218	Unknown		-3.97
BB_0617		hypothetical protein	-5.20	0.0213989	Unknown	-2.33	
BB_0595		hypothetical protein	-4.90	0.0034075	Unknown		
BB_0363		hypothetical protein	-4.85	0.0001982	Unknown	-2.11	
BB_0794		hypothetical protein	-4.24	4.72E-07	Unknown		
BB_0024		hypothetical protein	-4.14	1.72E-05	Unknown	-2.58	-4.92
BB_D13		hypothetical protein	-4.03	5.55E-08	Unknown		-3.78
BB_0323		hypothetical protein	-3.80	5.38E-07	Unknown		
BB_0261		hypothetical protein	-3.69	5.13E-06	Unknown		
BB_0569		hypothetical protein	-3.63	1.13E-07	Unknown		
BB_0397		hypothetical protein	-3.60	0.0166065	Unknown		
BB_0110		hypothetical protein	-3.59	0.0156739	Unknown		
BB_J23		hypothetical protein	-3.56	1.10E-06	Unknown	-2.07	
BB_0228		hypothetical protein	-3.54	0.0050987	Unknown		
BB_0650		hypothetical protein	-3.50	3.66E-07	Unknown		

BB_0028	hypothetical protein	-3.49	7.16E-07	Unknown		
BB_0345	hypothetical protein	-3.41	1.24E-06	Unknown	-2.01	
BB_0614	hypothetical protein	-3.18	7.41E-05	Unknown		
BB_0535	hypothetical protein	-3.15	0.0083064	Unknown		
BB_0072	hypothetical protein	-3.14	0.0006038	Unknown		
BB_0032	hypothetical protein	-3.03	0.006664	Unknown		
BB_0019	hypothetical protein	-2.99	0.001243	Unknown		
BB_0354	hypothetical protein	-2.92	0.0007373	Unknown		
BB_0124	hypothetical protein	-2.90	0.0003489	Unknown		
BB_0156	hypothetical protein	-2.85	0.0134276	Unknown		
BB_0662	hypothetical protein	-2.83	0.0004088	Unknown		
BB_0839	hypothetical protein	-2.73	0.0140067	Unknown		
BB_0752	hypothetical protein	-2.71	0.0011066	Unknown		
BB_0161	hypothetical protein	-2.70	0.000104	Unknown	-2.29	
BB_0661	hypothetical protein	-2.62	0.0001132	Unknown		
BB_0139	hypothetical protein	-2.60	2.53E-05	Unknown		-3.34
BB_0675	hypothetical protein	-2.60	0.0322228	Unknown		
BB_0210	hypothetical protein	-2.56	0.0388898	Unknown		
BB_0163	hypothetical protein	-2.49	7.69E-06	Unknown		
BB_0564	hypothetical protein	-2.45	0.0011932	Unknown		
BB_0731	hypothetical protein	-2.42	0.0050987	Unknown		
BB_0696	hypothetical protein	-2.38	4.40E-06	Unknown		
BB_0405	hypothetical protein	-2.36	8.84E-06	Unknown		
BB_0418	hypothetical protein	-2.33	0.0125928	Unknown		
BB_0648	hypothetical protein	-2.32	0.0490717	Unknown		
BB_0322	hypothetical protein	-2.31	1.64E-05	Unknown		
BB_0157	hypothetical protein	-2.30	0.0011616	Unknown		
BB_0631	hypothetical protein	-2.29	5.29E-05	Unknown	-2	-4.11
BB_0751	hypothetical protein	-2.28	0.0002035	Unknown	-2.22	
BB_0423	hypothetical protein	-2.26	3.04E-06	Unknown		
BB_0192	hypothetical protein	-2.25	0.0411471	Unknown		
BB_0252	hypothetical protein	-2.21	0.0030244	Unknown		
BB_0663	hypothetical protein	-2.21	0.0078689	Unknown		

BB_0126	hypothetical protein	-2.18	0.0256829	Unknown		
BB_K40	hypothetical protein	-2.13	1.34E-05	Unknown		
BB_0044	hypothetical protein	-2.13	0.0085875	Unknown	-2.71	
BB_0089	hypothetical protein	-2.11	0.0026725	Unknown		
BB_0541	hypothetical protein	-2.11	0.006215	Unknown		
BB_0818	hypothetical protein	-2.10	0.0024371	Unknown		
BB_0459	hypothetical protein	-2.09	0.0001289	Unknown	-2.47	
BB_R34	hypothetical protein	-2.08	0.0001068	Unknown		
BB_0359	hypothetical protein	-2.08	7.89E-05	Unknown		-1.96
BB_0816	hypothetical protein	-2.06	0.0013312	Unknown		
BB_0549	hypothetical protein	-2.05	0.0440098	Unknown		
BB_0319	hypothetical protein	-2.05	0.0074889	Unknown		
BB_0049	hypothetical protein	-2.04	0.0227149	Unknown		-2.36
BB_0070	hypothetical protein	-2.03	0.001834	Unknown		

Table S3. Genes differentially regulated by wild type in response to starvation condition.

ID	Symbol	Description	Relative expression RPMI / BSKII	FDR adjusted P-value	Category	Relative expression Δd*ksA* / WT during logarithmic growth
BB_L01		phage portal protein	4.5	1.00E-06	Bacteriophage	11.01
BB_L23	*blyA*	BlyA family holin	8.08	0.004621	Bacteriophage	3.64
BB_0443		spoIIIJ-associtated protein	-5.04	0	Cell Division	
BB_0817	*murC*	UDP-N-acetylmuramate--L-alanine ligase	-4.27	1.00E-06	Cell Division	
BB_B03	*resT*	telomere resolvase	-2.79	9.60E-05	Cell Division	
BB_A20		PF-32 Protein	-2.56	0.045462	Cell Division	-2.89
BB_0195		cell division control protein 27	2.01	0	Cell Division	
BB_B12		PF-32 Protein	2.17	0.023364	Cell Division	
BB_0715		cell division protein FtsA	2.19	4.30E-05	Cell Division	
BB_0598	*murB*	UDP-N-acetylenolpyruvoylglucosamine reductase	2.32	0.016245	Cell Division	
BB_F24		PF-32 Protein	2.89	1.00E-05	Cell Division	3.40
BB_J18		PF-32 Protein	3.2	0.006731	Cell Division	2.09
BB_N32		PF-32 Protein	3.57	0.008724	Cell Division	
BB_0431		CobQ/CobB/MinD/ParA nucleotide binding domain-containing protein	3.7	9.60E-05	Cell Division	3.47
BB_F23		PF-32 Protein	3.77	0.018732	Cell Division	
BB_J16		Plasmid partitioning protein	3.92	2.90E-05	Cell Division	
BB_M32		PF-32 Protein	4.39	0.000265	Cell Division	
BB_K24		PF-32 Protein	5.62	0	Cell Division	
BB_B13		Plasmid partitioning protein	7.3	0.001141	Cell Division	
BB_Q07		Plasmid partitioning protein	19.81	0.00017	Cell Division	
BB_0574		integral membrane protein	-8.39	0.000615	Cell Envelope	
BB_0193		lipoprotein	-6.01	0.012091	Cell Envelope	
BB_0234		integral membrane protein	-5.83	9.00E-06	Cell Envelope	

BB_0664		lipoprotein	-5.01	5.90E-05	Cell Envelope	
BB_0317		Integral Membrane protein	-4.59	0.000681	Cell Envelope	
BB_0353		membrane protein	-3.94	6.00E-06	Cell Envelope	-2.05
BB_0806		lipoprotein	-3.82	7.00E-06	Cell Envelope	
BB_0155		lipoprotein	-3.79	0.000196	Cell Envelope	-2.95
BB_0398		lipoprotein	-3.05	1.00E-05	Cell Envelope	
BB_K15		antigen, P35	-3.02	0.001636	Cell Envelope	
BB_F20		lipoprotein	-2.65	0.000421	Cell Envelope	-2.53
BB_S42	*bapA*	BapA protein	-2.56	0.002471	Cell Envelope	
BB_0108		basic membrane protein	-2.42	0.005115	Cell Envelope	
BB_0172		von Willebrand factor type A domain-containing	-2.42	0.002032	Cell Envelope	
BB_0071		membrane protein	-2.01	0.007912	Cell Envelope	-4.49
BB_0473		Integral membrane protein	2.06	0.003515	Cell Envelope	3.56
BB_0732		penicillin-binding protein	2.08	0.000224	Cell Envelope	
BB_J08		surface protein	2.17	2.00E-06	Cell Envelope	
BB_0719	*mrdB*	rod shape-determining protein RodA	2.33	0.000166	Cell Envelope	2.03
BB_K32		fibronectin-binding protein	2.55	1.40E-05	Cell Envelope	
BB_A52		outer membrane protein	2.6	0.000168	Cell Envelope	2.52
BB_J34		lipoprotein	2.61	0.001304	Cell Envelope	
BB_L40	*erpO*	ErpO protein	2.77	2.00E-06	Cell Envelope	
BB_0167		outer membrane protein	2.78	0.045462	Cell Envelope	2.61
BB_0603	*p66*	membrane-associated protein p66	2.83	5.00E-06	Cell Envelope	
BB_0136		penicillin-binding protein	2.85	0.004309	Cell Envelope	
BB_O28	*mlpG*	mlpG lipoprotein	2.89	2.90E-05	Cell Envelope	
BB_A68	*cspA*	complement regulator-acquiring surface protein 1	2.91	0.000959	Cell Envelope	3.82
BB_N39	*erpQ*	ErpQ protein	3.14	8.10E-05	Cell Envelope	
BB_K48		immunogenic protein P37	3.7	8.10E-05	Cell Envelope	
BB_K53		outer membrane protein	3.8	0	Cell Envelope	
BB_L39	*erpN*	ErpN protein	3.89	1.30E-05	Cell Envelope	
BB_0584		integral membrane protein	4.03	0.000165	Cell Envelope	4.69
BB_O40	*erpM*	ErMG protein	4.41	1.00E-06	Cell Envelope	
BB_K01		lipoprotein	4.69	4.00E-06	Cell Envelope	

BB_N28	*mlpI*	mlpI lipoprotein	4.81	0.001156	Cell Envelope	
BB_0117		hemolysin III	4.94	0.017783	Cell Envelope	6.32
BB_A60	*p27*	surface lipoprotein P27	4.96	5.00E-06	Cell Envelope	
BB_S41	*erpG*	ErpG protein	5.08	1.00E-06	Cell Envelope	2.43
BB_R42	*erpY*	ErpY protein	5.11	0.032463	Cell Envelope	
BB_Q06		membrane protein	5.2	0.002945	Cell Envelope	
BB_K50		immunogenic protein P37	5.57	3.00E-06	Cell Envelope	6.08
BB_J36		lipoprotein	5.67	0	Cell Envelope	
BB_A36		lipoprotein	5.73	0	Cell Envelope	
BB_0213		lipoprotein	5.85	0.002526	Cell Envelope	
BB_0017		integral membrane protein	6.1	0.002095	Cell Envelope	5.10
BB_0844		lipoprotein	6.11	0	Cell Envelope	
BB_R28	*mlpD*	mlpD	6.13	2.50E-05	Cell Envelope	2.50
BB_K19		lipoprotein	6.28	0	Cell Envelope	
BB_M27	*revA*	RevA fibronectin binding protein	6.44	1.70E-05	Cell Envelope	
BB_Q03		outer membrane protein	6.58	0.001829	Cell Envelope	
BB_Q35	*mlpJ*	multicopy lipoprotein J	7.1	0.003706	Cell Envelope	
BB_A59		lipoprotein	7.34	0	Cell Envelope	
BB_L28	*mlpH*	lipoprotein MlpH	7.37	0	Cell Envelope	
BB_A04	*S2*	S2 Antigen	7.44	7.20E-05	Cell Envelope	
BB_0383	*bmpA*	basic membrane protein A	8.17	0	Cell Envelope	2.43
BB_0034		outer membrane protein P13	8.76	0	Cell Envelope	
BB_A69		putative surface protein	9.01	0	Cell Envelope	10.72
BB_O39	*erpL*	ErpL protein	9.4	0	Cell Envelope	
BB_S30	*mlpE*	mlpC	9.8	3.00E-06	Cell Envelope	
BB_M38	*erpK/ospF*	ErpK/ospF protein	9.83	0	Cell Envelope	
BB_H41		membrane protein	10.25	0.000217	Cell Envelope	
BB_A03		outer membrane protein	11.17	0	Cell Envelope	3.59
BB_P28	*mlpA*	surface lipoprotein	11.66	0	Cell Envelope	
BB_M28	*mlpF*	lipoprotein mlpF	13.09	0	Cell Envelope	
BB_0365	*la7*	lipoprotein LA7	13.9	0	Cell Envelope	5.95
BB_A57		P45-13	14.57	5.00E-06	Cell Envelope	10.06
BB_K07		lipoprotein	15.12	0	Cell Envelope	3.51

BB_J09	*ospD*	outer surface protein D	33.88	0	Cell Envelope	4.12
BB_0286		flagellar protein	-10.66	1.00E-06	Chemotaxis and motility	-2.06
BB_0287	*flbD*	flagellar protein FlbA	-5.9	0.001924	Chemotaxis and motility	
BB_0274	*fliQ*	flagellar biosynthesis protein FliQ	-4.61	0.032696	Chemotaxis and motility	
BB_0149	*fliD*	flagellar hook-associated protein FliD	-4.09	1.00E-06	Chemotaxis and motility	
BB_0270	*flhF*	flagellar biosynthesis regulator FlhF	-3.96	0	Chemotaxis and motility	-2.00
BB_0284	*flgD*	flagellar hook capping protein	-3.51	0.000289	Chemotaxis and motility	
BB_0276	*fliZ*	flagellar biosynthesis protein FliZ	-3.47	0.001114	Chemotaxis and motility	
BB_0289	*fliH*	flagellar assembly protein H	-2.89	9.60E-05	Chemotaxis and motility	
BB_0597	*mcp3*	methyl-accepting chemotaxis protein	-2.83	4.20E-05	Chemotaxis and motility	-3.89
BB_0290	*fliG*	flagellar motor switch protein G	-2.69	2.00E-06	Chemotaxis and motility	
BB_0550	*fliS*	flagellar protein FliS	-2.46	0.022746	Chemotaxis and motility	
BB_0282	*flbD*	flagellar protein FlbD	-2.45	0.000143	Chemotaxis and motility	
BB_0288	*fliI*	flagellum-specific ATP synthase FliI	-2.37	0.00039	Chemotaxis and motility	
BB_0568	*CheB*	chemotaxis protein CheB	-2.17	3.70E-05	Chemotaxis and motility	
BB_0596		methyl-accepting chemotaxis protein	-2.13	0.004492	Chemotaxis and motility	-3.58
BB_0279	*fliL*	flagellar basal body-associated protein FliL	-2.06	0.001198	Chemotaxis and motility	
BB_0271	*flhA*	flagellar biosynthesis protein FlhA	-2.05	0.001434	Chemotaxis and motility	
BB_0147	*flaB*	flagellar filament 41 kDa core protein	7.51	0	Chemotaxis and motility	
BB_0438	*dnaN*	DNA polymerase III subunit beta	-5.18	0	DNA replication and repair	-2.82
BB_0457	*uvrC*	excinuclease ABC subunit C	-3.86	0.006927	DNA replication and repair	-2.62
BB_0114		single-stranded DNA-binding protein	-3.53	1.00E-06	DNA replication and repair	
BB_0435	*gyrA*	DNA gyrase subunit A	-3.43	0	DNA replication and repair	
BB_0111	*dnaB*	replicative DNA helicase	-3.22	0.001459	DNA replication and repair	
BB_0254	*recJ*	single-stranded-DNA-specific exonuclease	-2.67	0.031751	DNA replication and repair	-2.63
BB_0534	*xth*	exodeoxyribonuclease III	-2.66	0.000115	DNA replication and repair	
BB_0232	*hbb*	HbbU protein	-2.61	0.000114	DNA replication and repair	
BB_0436	*gyrB*	DNA gyrase subunit B	-2.41	0.000439	DNA replication and repair	
BB_0297		smf protein	-2.3	9.70E-05	DNA replication and repair	
BB_0581	*recG*	ATP-dependent DNA helicase RecG	-2.18	0.010905	DNA replication and repair	
BB_0035	*parC*	DNA topoisomerase IV subunit A	2.46	0.000522	DNA replication and repair	2.57
BB_0836	*uvrB*	excinuclease ABC, B subunit (uvrB)	2.82	0.000347	DNA replication and repair	2.63
BB_0745	*nth*	endonuclease III	2.85	0.03429	DNA replication and repair	

BB_0798	*comF*	competence protein F	2.87	0.000309	DNA replication and repair	
BB_G32		replicative DNA helicase	3	4.40E-05	DNA replication and repair	4.61
BB_0014	*priA*	primosomal protein N	3.11	2.40E-05	DNA replication and repair	
BB_0633	*recB*	exodeoxyribonuclease V subunit beta	4.56	1.00E-06	DNA replication and repair	
BB_0053	*ung*	uracil-DNA glycosylase	7.58	8.00E-05	DNA replication and repair	3.52
BB_0248	*pepF*	oligoendopeptidase F	-12.07	0.000352	Metabolism	-6.88
BB_0243	*glpD*	glycerol-3-phosphate dehydrogenase	-4.8	3.50E-05	Metabolism	-14.05
BB_0791		thymidine kinase	-4.65	0	Metabolism	
BB_0683	*hmgs*	3-hydroxy-3-methylglutaryl-CoA synthase	-4.07	1.30E-05	Metabolism	
BB_0259		transglycosylase SLT domain protein	-3.72	0.000161	Metabolism	
BB_0812	*coaBC*	bifunctional phosphopantothenoylcysteine decarboxylase/phosphopantothenate--cysteine ligase	-3.5	0.000219	Metabolism	
BB_0249		phosphatidylcholine synthase	-3.43	0.009466	Metabolism	-4.05
BB_0120	*uppS*	undecaprenyl pyrophosphate synthase	-3.25	3.10E-05	Metabolism	-2.00
BB_0381	*treA*	trehalase	-3.16	1.00E-05	Metabolism	
BB_0544		phosphoribosylpyrophosphate synthetase	-3.07	2.00E-06	Metabolism	
BB_0241	*glpK*	glycerol kinase	-2.84	6.70E-05	Metabolism	-23.09
BB_0444		nucleotide sugar epimerase	-2.82	4.00E-06	Metabolism	
BB_0709		aminodeoxychorismate lyase, putative	-2.76	0.000331	Metabolism	
BB_0061	*trx*	thioredoxin	-2.64	3.00E-06	Metabolism	
BB_0782	*nadD*	nicotinate (nicotinamide) nucleotide adenylyltransferase	-2.61	0.000179	Metabolism	
BB_0819		cytidylate kinase	-2.49	2.70E-05	Metabolism	-2.59
BB_0725		serine-type D-Ala-D-Ala carboxypeptidase	-2.43	0.010543	Metabolism	
BB_0467		laccase domain containing protein	-2.4	0.015287	Metabolism	
BB_0575	*pyrG*	CTP synthetase	-2.13	0.018686	Metabolism	
BB_0137		long-chain-fatty-acid CoA ligase	2.01	6.10E-05	Metabolism	
BB_0445	*fbaA*	fructose-bisphosphate aldolase	2.1	7.40E-05	Metabolism	
BB_0463	*ndk*	nucleoside diphosphate kinase	2.25	7.00E-06	Metabolism	
BB_0621		4-methyl-5(b-hydroxyethyl)-thiazole monophosphate biosynthesis protein	2.35	0	Metabolism	7.70
BB_0377	*luxS*	S-ribosylhomocysteinase	2.36	0.002831	Metabolism	

BB_0630	*pfkB*	1-phosphofructokinase	2.41	0.001102	Metabolism	
BB_A76	*thyX*	FAD-dependent thymidylate synthase	2.49	4.60E-05	Metabolism	6.86
BB_0588	*bgp*	MTA/SAH nucleosidase	2.51	0.000201	Metabolism	
BB_0084		cysteine desulfurase	2.6	0.002428	Metabolism	3.07
BB_0593	*fadD*	long-chain-fatty-acid CoA ligase	2.65	0.000548	Metabolism	
BB_0561	*gnd*	6-phosphogluconate dehydrogenase	2.82	0	Metabolism	2.23
BB_0622	*ackA*	acetate kinase	2.83	0.000734	Metabolism	
BB_0004	*pgm*	phosphoglucomutase	2.9	0.027206	Metabolism	
BB_0601	*glyA*	serine hydroxymethyltransferase	3.08	1.00E-06	Metabolism	2.39
BB_K17		adenine deaminase	3.1	9.70E-05	Metabolism	3.89
BB_0166	*malQ*	4-alpha-glucanotransferase	3.11	5.00E-06	Metabolism	
BB_0768		pyridoxal kinase	3.23	0.006772	Metabolism	
BB_0770		divergent polysaccharide deacetylase superfamily protein	3.24	1.00E-05	Metabolism	
BB_0677	*mglA*	ribose/galactose ABC transporter, ATP-binding protein (mglA)	3.32	1.40E-05	Metabolism	5.49
BB_0629	*fruA2*	pts system, fructose-specific iiabc component	3.59	1.00E-06	Metabolism	
BB_0524		inositol monophosphatase	3.66	1.20E-05	Metabolism	
BB_0109		acetyl-CoA C-acetyltransferase	3.87	0	Metabolism	4.30
BB_0605		serine-type D-Ala-D-Ala carboxypeptidase	4.1	1.00E-06	Metabolism	6.04
BB_0608		aminoacyl-histidine dipeptidase	4.11	2.00E-06	Metabolism	
BB_0657	*rpiA*	ribose 5-phosphate isomerase	4.24	1.00E-06	Metabolism	
BB_0635	*pncB*	nicotinate phosphoribosyltransferase	4.8	0.000377	Metabolism	3.23
BB_0407	*manA*	mannose-6-phosphate isomerase	4.86	0	Metabolism	3.30
BB_0057	*gap*	glyceraldehyde 3-phosphate dehydrogenase	5.11	1.00E-06	Metabolism	
BB_0658		phosphoglycerate mutase	5.47	0	Metabolism	
BB_0417		adenylate kinase	5.67	0.000581	Metabolism	
BB_0337	*eno*	enolase	5.68	0	Metabolism	
BB_0793	*tmk*	thymidylate kinase	5.8	0.000563	Metabolism	4.35
BB_0644		N-acetylmannosamine-6-phosphate 2-epimerase	6.35	1.00E-06	Metabolism	6.12
BB_0760	*mazG*	gene 37 protein (Gp37)	6.55	1.00E-06	Metabolism	8.01
BB_0515	*trxB;*	thioredoxin reductase	7.04	0	Metabolism	4.12

BB_0408		PTS system, fructose-specific IIABC component (fruA-1)	12.72	0	Metabolism	5.16
BB_0364	*mgsA*	methylglyoxal synthase	12.88	0	Metabolism	2.53
BB_0604		L-lactate permease	18.56	0	Metabolism	
BB_0645	*ptsG*	PTS system, glucose-specific IIBC component (ptsG)	21.9	0	Metabolism	4.84
BB_0612	*clpX*	ATP-dependent protease ATP-binding subunit ClpX	-3.91	0	Protein degradation	
BB_0253	*lon*	ATP-dependent protease LA	-3.14	0	Protein degradation	-2.86
BB_0536	*pqqL*	zinc protease	-2.93	0.000117	Protein degradation	-6.01
BB_0246		M23 peptidase domain-containing protein	-2.49	0.000454	Protein degradation	
BB_0067		peptidase	-2.14	0	Protein degradation	
BB_0296	*hslV*	ATP-dependent protease subunit HslV	-2.02	0.046379	Protein degradation	
BB_0834		ATP-dependent Clp protease subunit C	2.62	0.000852	Protein degradation	
BB_0757	*clpP*	Clp protease	2.78	1.00E-06	Protein degradation	3.22
BB_0761	*nlpD*	peptidoglycan-binding protein	2.96	0.019335	Protein degradation	9.82
BB_A71		pseudogene	-4.56	0.00257	Pseudogene	-17.72
BB_F16		pseudogene	-4.21	0.000223	Pseudogene	
BB_0522		pseudogene	-3.75	0.000249	Pseudogene	
BB_0511		pseudogene	-3.73	0.000173	Pseudogene	
BB_0140		pseudogene	-2.9	0.000143	Pseudogene	
BB_0021		queA	-2.81	0.005675	Pseudogene	
BB_B11		pseudogene	-2.81	0.03085	Pseudogene	
BB_J10		pseudogene	2.33	0.019959	Pseudogene	7.74
BB_A14		pseudogene	3.39	0.031266	Pseudogene	5.05
BB_M22		pseudogene	3.74	0.000283	Pseudogene	3.14
BB_0530		pseudogene	3.78	0.00892	Pseudogene	
BB_G06		pseudogene	4.33	0	Pseudogene	2.27
BB_A55		pseudogene	4.91	1.30E-05	Pseudogene	7.31
BB_A22		pseudogene	7.51	5.00E-06	Pseudogene	3.18
BB_K39		pseudogene	8.24	7.00E-06	Pseudogene	
BB_F10		pseudogene	9.57	0.01396	Pseudogene	
BB_F22		pseudogene	10.91	0	Pseudogene	5.24

BB_Q01		pseudogene	15.69	2.30E-05	Pseudogene	
BB_F32		pseudogene	15.9	0	Pseudogene	6.19
BB_Q57		pseudogene	19.7	0	Pseudogene	
BB_0295	*hslU*	heat shock protein HslVU, ATPase subunit HslU	-2.74	0.001674	Stress Response	
BB_0519	*grpE*	GrpE protein	2.32	0.001171	Stress Response	3.88
BB_0518	*dnaK*	molecular chaperone DnaK	3.01	0	Stress Response	2.72
BB_0741	*groS*	chaperonin GroS	3.26	1.00E-05	Stress Response	
BB_0517	*dnaJ*	chaperone protein DnaJ	5.14	2.00E-06	Stress Response	2.18
BB_0107	*nusB*	transcription antitermination factor NusB	-7.59	0	Transcription and regulation	
BB_0060		endoribonuclease	-7.39	0	Transcription and regulation	
BB_0502	*rpoA*	DNA-directed RNA polymerase subunit alpha	-6.17	0	Transcription and regulation	-3.52
BB_0712	*rpoD*	RNA polymerase sigma factor RpoD	-3.69	8.00E-06	Transcription and regulation	
BB_0198	*rel*	guanosine-3',5'-bis(diphosphate) 3'-pyrophosphohydrolase	-3.59	0.001886	Transcription and regulation	-7.46
BB_0132	*greA*	transcription elongation factor GreA	-3.49	1.00E-06	Transcription and regulation	
BB_0820	*rpoZ*	DNA-directed RNA polymerase subunit omega	-3.48	2.10E-05	Transcription and regulation	-2.47
BB_0419	*rrp-1*	response regulatory protein 1	-2.81	0.019055	Transcription and regulation	
BB_0504		ribonuclease Y	-2.71	6.60E-05	Transcription and regulation	
BB_0800		transcription elongation factor NusA	-2.4	9.30E-05	Transcription and regulation	
BB_0388	*rpoC*	DNA-directed RNA polymerase subunit beta'	-2.16	6.00E-06	Transcription and regulation	
BB_0693	*badR*	xylose operon regulatory protein	2.32	1.20E-05	Transcription and regulation	2.35
BB_0416		pheromone shutdown protein	2.63	0.000893	Transcription and regulation	
BB_0771	*rpoS*	RNA polymerase sigma factor	3.73	5.10E-05	Transcription and regulation	
BB_D18		hypothetical protein (rpoS repression)	4.27	1.40E-05	Transcription and regulation	5.98
BB_0168	*dksA*	dnaK suppressor	4.36	0.048919	Transcription and regulation	
BB_0184	*csrA*	carbon storage regulator	6.79	6.50E-05	Transcription and regulation	
BB_0803	*truB*	tRNA pseudouridine 55 synthase	-10.81	0	Translation	
BB_0005	*trpS*	tryptophanyl-tRNA synthetase	-7.47	2.00E-06	Translation	
BB_0122	*tsf*	elongation factor Ts	-7.38	0	Translation	-2.55
BB_0031	*lepB*	signal peptidase I	-4.77	4.00E-06	Translation	
BB_0088	*lepA*	elongation factor EF-4	-4.24	0	Translation	-2.21
BB_0343	*gatC*	aspartyl/glutamyl-tRNA amidotransferase subunit C	-4.24	0.000527	Translation	

BB_0235	*ychF*	GTP-binding protein YchF	-3.95	3.00E-06	Translation	
BB_0697	*rimM*	16S rRNA-processing protein RimM	-3.86	2.00E-06	Translation	
BB_0033	*smpB*	SsrA-binding protein	-3.68	0.005293	Translation	-6.74
BB_0121	*frr*	ribosome recycling factor	-3.31	2.00E-05	Translation	-2.11
BB_0113	*rpsR*	30S ribosomal protein S18	-3.25	1.30E-05	Translation	
BB_0030	*lepB*	signal peptidase I	-3.18	0.002411	Translation	
BB_0499	*rpmJ*	50S ribosomal protein L36	-3.17	0.000279	Translation	
BB_0123	*rpsB*	30S ribosomal protein S2	-3.08	0	Translation	-2.17
BB_0486	*rpmC*	50S ribosomal protein L29	-3.02	0.000303	Translation	
BB_0653	*secF*	preprotein translocase subunit SecF	-3.02	1.30E-05	Translation	-2.50
BB_0233	*rpsT*	30S ribosomal protein S20	-2.95	1.00E-06	Translation	
BB_0346		export chaperone	-2.92	0.000494	Translation	-2.79
BB_0115		30S ribosomal protein S6	-2.91	1.30E-05	Translation	
BB_0112	*rplI*	50S ribosomal protein L9	-2.8	3.00E-06	Translation	-2.74
BB_0610	*tig*	trigger factor	-2.7	0.0021	Translation	
BB_0643	*ylqF*	GTPase YlqF	-2.65	0.010774	Translation	
BB_0204	*hflC*	HflC protein	-2.61	0.000438	Translation	-3.27
BB_0500	*rpsM*	30S ribosomal protein S13	-2.47	5.70E-05	Translation	
BB_0497	*rplO*	50S ribosomal protein L15	-2.45	3.50E-05	Translation	
BB_0489	*rplX*	50S ribosomal protein L24	-2.42	0.003357	Translation	
BB_0370	*tyrS*	tyrosyl-tRNA synthetase	-2.35	0.000255	Translation	
BB_0695	*rpsP*	30S ribosomal protein S16	-2.33	3.00E-06	Translation	-2.05
BB_0064	*fmt*	methionyl-tRNA formyltransferase	-2.31	1.00E-06	Translation	
BB_0801	*infB*	translation initiation factor IF-2	-2.3	5.00E-06	Translation	
BB_0496		50S ribosomal protein L30	-2.26	7.10E-05	Translation	
BB_0508	*engA*	GTP-binding protein Der	-2.26	0.002171	Translation	
BB_0492	*rpsH*	30S ribosomal protein S8	-2.21	1.40E-05	Translation	
BB_0786		50S ribosomal protein L25/general stress protein	-2.18	0.000629	Translation	
BB_0493		50S ribosomal protein L6	-2.17	0.000185	Translation	
BB_0127		30S ribosomal protein S1	-2.13	5.80E-05	Translation	-2.51
BB_0802	*rbfA*	ribosome-binding factor A	-2.09	1.80E-05	Translation	
BB_0442	*yidC*	membrane protein insertase YidC	-2.08	1.40E-05	Translation	
BB_0395	*secE*	preprotein translocase subunit SecE	-2.03	0.002575	Translation	

BB_0229	*rpmE*	50S ribosomal protein L31	2.04	0.000153	Translation	
BB_0390	*rplL*	50S ribosomal protein L7/L12	2.06	9.00E-06	Translation	
BB_0427		rRNA small subunit methyltransferase I	2.15	0.009449	Translation	10.91
BB_0313	*rrmJ*	ribosomal RNA large subunit methyltransferase J	2.24	0.000175	Translation	
BB_0738	*valS*	valyl-tRNA synthetase	2.28	5.00E-06	Translation	
BB_0251	*leuS*	leucyl-tRNA synthetase	2.34	0.005878	Translation	
BB_0338	*rpsI*	30S ribosomal protein S9	2.36	5.00E-05	Translation	
BB_0769	*tsaD*	UGMP family protein	2.55	0.002727	Translation	
BB_0446	*aspS*	aspartyl-tRNA synthetase	2.65	4.00E-06	Translation	
BB_0833	*ileS*	isoleucyl-tRNA synthetase	2.68	0.009654	Translation	
BB_0214	*efp*	elongation factor P	2.83	1.30E-05	Translation	4.22
BB_0482	*rpsS*	30S ribosomal protein S19	3.13	8.00E-06	Translation	2.17
BB_0135	*hisS*	histidyl-tRNA synthetase	3.78	4.00E-06	Translation	4.55
BB_0143		membrane protein insertion efficiency factor	4.11	1.00E-06	Translation	2.71
BB_0256	*rpsU*	30S ribosomal protein S21	4.16	0	Translation	2.14
BB_0196	*prfA*	peptide chain release factor 1	4.25	2.00E-06	Translation	
BB_0660	*era*	GTPase Era	4.3	3.00E-06	Translation	2.11
BB_0263	*lepB*	signal peptidase I	4.31	0.003998	Translation	7.16
BB_0169	*infA*	translation initiation factor IF-1	4.75	0.000117	Translation	2.65
BB_0476	*tuf*	elongation factor Tu	5.01	1.40E-05	Translation	
BB_0225		tRNA-dihydrouridine synthase A	8.78	0.006922	Translation	
BB_0318		methylgalactoside ABC transporter ATP-binding	-3.68	6.00E-06	Transporter Proteins	-2.00
BB_0466		ABC transporter ATP-binding protein	-3.32	0	Transporter Proteins	
BB_0141	*besA*	membrane fusion protein	-3.02	0.004492	Transporter Proteins	
BB_0146		glycine/betaine ABC transporter ATP-binding protein	-2.9	4.00E-05	Transporter Proteins	
BB_0573		ABC transporter ATP-binding protein	-2.82	0.000439	Transporter Proteins	
BB_0042	*phoU*	phosphate transport system regulatory protein	-2.72	4.00E-05	Transporter Proteins	
BB_0808		hypothetical protein (lipopolysaccharide export system permease protein)	-2.15	0.020888	Transporter Proteins	
BB_0742		ABC transporter ATP-binding protein	-2.13	0.000135	Transporter Proteins	
BB_J26		ABC transporter, ATP-binding protein	-2.1	0.010073	Transporter Proteins	

BB_0145		glycine/betaine ABC transporter permease	-2.05	0.002023	Transporter Proteins	
BB_0329	*oppA-2*	oligopeptide permease peptide binding protein	-2.05	0.00059	Transporter Proteins	-7.41
BB_B06	*chbB*	chitobiose transporter protein	-2.02	0.01931	Transporter Proteins	
BB_B29	*malX2*	PTS system transporter subunit IIBC	2.22	0.002571	Transporter Proteins	
BB_0729	*yhcL*	dicarboxylate/amino acid:cation symporter	2.26	0.000321	Transporter Proteins	
BB_0559	*ccr*	PTS system glucose-specific transporter	2.42	2.00E-06	Transporter Proteins	
BB_0332		peptide ABC transporter permease	2.46	0	Transporter Proteins	
BB_0334	*oppD*	peptide ABC transporter ATP-binding protein	2.49	9.40E-05	Transporter Proteins	
BB_B05	*chbA*	chitobiose transporter protein	2.79	0.01609	Transporter Proteins	4.42
BB_B04	*chcB*	chitobiose transporter protein	2.84	0.000128	Transporter Proteins	
BB_0447		Na+/H+ antiporter (putative)	3.08	7.00E-06	Transporter Proteins	3.66
BB_0218	*pstB*	phosphate ABC transporter ATP-binding protein	3.2	0.031751	Transporter Proteins	
BB_0638		Na+/H+ antiporter	3.72	0.001125	Transporter Proteins	
BB_0219		metal cation transporter permease gufA protein	3.74	2.00E-06	Transporter Proteins	
BB_0637		Na+/H+ antiporter family	4.02	5.00E-06	Transporter Proteins	
BB_A74	*oms28*	outer membrane porin	4.24	0	Transporter Proteins	5.83
BB_B23	*pbuG*	guanine/xanthine permease	5.44	3.00E-05	Transporter Proteins	6.92
BB_0335	*oppF*	peptide ABC transporter ATP-binding protein	5.77	1.80E-05	Transporter Proteins	
BB_A34	*oppA5*	extracellular solute-binding protein, family 5	6.17	5.00E-06	Transporter Proteins	
BB_0453		small conductance mechanosensitive ion channel	9.7	1.00E-06	Transporter Proteins	4.61
BB_0242		hypothetical protein	-46.52	1.00E-06	Unknown	-29.70
BB_0319		hypothetical protein	-14.34	0	Unknown	-2.05
BB_0126		hypothetical protein	-11.07	1.40E-05	Unknown	-2.18
BB_0011		hypothetical protein	-10.81	3.70E-05	Unknown	-5.78
BB_0535		hypothetical protein	-7.3	0.000107	Unknown	-3.15
BB_0298		TPR domain protein	-7.13	1.00E-06	Unknown	
BB_0675		hypothetical protein	-6.84	0.000271	Unknown	-2.60
BB_0439		hypothetical protein	-6.67	4.00E-06	Unknown	
BB_0713		hypothetical protein	-6.5	0	Unknown	
BB_0227		hypothetical protein	-6.26	0	Unknown	
BB_0110		hypothetical protein	-6.18	0.000698	Unknown	-3.59
BB_0397		hypothetical protein	-5.89	0.000631	Unknown	-3.60

BB_0818	hypothetical protein	-5.71	4.00E-06	Unknown	-2.10
BB_0792	hypothetical protein	-5.58	1.00E-06	Unknown	
BB_0320	hypothetical protein	-5.34	0.000165	Unknown	
BB_0266	hypothetical protein	-4.98	2.00E-05	Unknown	
BB_0063	hypothetical protein	-4.85	0	Unknown	
BB_0468	hypothetical protein	-4.78	1.00E-06	Unknown	
BB_0794	hypothetical protein	-4.63	0	Unknown	-4.24
BB_0336	hypothetical protein	-4.57	4.50E-05	Unknown	
BB_0796	hypothetical protein	-4.48	0.003828	Unknown	
BB_0322	hypothetical protein	-4.23	0	Unknown	-2.31
BB_0661	hypothetical protein	-4.13	3.00E-06	Unknown	-2.62
BB_0260	hypothetical protein	-4.09	0.000999	Unknown	
BB_0058	hypothetical protein	-4.07	1.00E-06	Unknown	
BB_0223	hypothetical protein	-3.99	0.000969	Unknown	
BB_A72	hypothetical protein	-3.97	1.30E-05	Unknown	-16.43
BB_0692	hypothetical protein	-3.77	0.030077	Unknown	
BB_0356	hypothetical protein	-3.74	0.003073	Unknown	
BB_0345	hypothetical protein	-3.72	1.00E-06	Unknown	-3.41
BB_0707	hypothetical protein	-3.72	0.001796	Unknown	
BB_0564	hypothetical protein	-3.71	6.90E-05	Unknown	-2.45
BB_0700	hypothetical protein	-3.71	0.002411	Unknown	
BB_0700	hypothetical protein	-3.71	0.002411	Unknown	
BB_0059	CBS domain-containing protein - hemolysin C	-3.65	1.00E-06	Unknown	
BB_0002	hypothetical protein	-3.59	0.002427	Unknown	
BB_0619	DHH family	-3.47	1.80E-05	Unknown	
BB_0066	hypothetical protein	-3.46	4.90E-05	Unknown	
BB_0192	hypothetical protein	-3.43	0.001931	Unknown	-2.25
BB_0400	hypothetical protein	-3.39	0.002701	Unknown	
BB_0701	hypothetical protein	-3.29	0.002761	Unknown	
BB_0231	hypothetical protein	-3.27	0	Unknown	
BB_0662	hypothetical protein	-3.06	0.000265	Unknown	-2.83
BB_0363	hypothetical protein	-3.05	0.003581	Unknown	-4.85
BB_0170	hypothetical protein	-3.04	9.00E-06	Unknown	

BB_0458	hypothetical protein	-3.04	0	Unknown	
BB_0174	hypothetical protein	-3.03	0.000853	Unknown	
BB_0403	hypothetical protein	-2.99	3.00E-05	Unknown	
BB_0409	hypothetical protein	-2.99	0.016415	Unknown	
BB_0554	hypothetical protein	-2.98	2.00E-05	Unknown	
BB_0790	hypothetical protein	-2.97	0.000234	Unknown	
BB_0019	hypothetical protein	-2.9	0.002156	Unknown	-2.99
BB_0125	hypothetical protein	-2.81	8.00E-06	Unknown	
BB_0418	hypothetical protein	-2.77	0.003705	Unknown	-2.33
BB_0711	hypothetical protein	-2.71	0.00013	Unknown	
BB_0044	hypothetical protein	-2.7	0.002352	Unknown	-2.13
BB_0089	hypothetical protein	-2.67	0.000479	Unknown	-2.11
BB_0156	hypothetical protein	-2.67	0.005365	Unknown	-2.85
BB_0470	hypothetical protein	-2.67	6.80E-05	Unknown	
BB_0569	hypothetical protein	-2.62	3.00E-06	Unknown	-3.63
BB_0471	hypothetical protein	-2.6	0.000265	Unknown	
BB_0538	hypothetical protein	-2.58	0.000232	Unknown	
BB_0459	hypothetical protein	-2.53	1.60E-05	Unknown	-2.09
BB_0708	hypothetical protein	-2.51	0.015153	Unknown	
BB_0373	hypothetical protein	-2.47	0.000962	Unknown	
BB_N14	hypothetical protein	-2.43	0.010229	Unknown	
BB_0106	TPR domain protein	-2.37	3.10E-05	Unknown	
BB_0007	hypothetical protein	-2.35	9.40E-05	Unknown	
BB_0307	hypothetical protein	-2.32	0.00018	Unknown	
BB_0624	hypothetical protein	-2.32	1.80E-05	Unknown	
BB_0663	hypothetical protein	-2.3	0.005273	Unknown	-2.21
BB_0617	hypothetical protein	-2.29	0.036744	Unknown	-5.20
BB_0043	hypothetical protein	-2.23	3.10E-05	Unknown	
BB_0665	hypothetical protein	-2.23	0.00095	Unknown	
BB_0648	hypothetical protein	-2.2	0.017091	Unknown	-2.32
BB_0171	hypothetical protein	-2.17	0.000534	Unknown	
BB_0163	hypothetical protein	-2.14	9.70E-05	Unknown	-2.49
BB_0261	hypothetical protein	-2.11	0.000999	Unknown	-3.69

BB_0130	hypothetical protein	-2.09	0.015602	Unknown	
BB_0268	hypothetical protein	-2.08	0.00095	Unknown	
BB_0267	hypothetical protein	-2.07	5.00E-05	Unknown	
BB_B28	hypothetical protein	-2.07	0.004433	Unknown	
BB_0354	hypothetical protein	-2.06	0.007237	Unknown	-2.92
BB_0070	hypothetical protein	-2.03	0.003543	Unknown	-2.03
BB_0209	hypothetical protein	-2.01	0.00024	Unknown	
BB_0159	hypothetical protein	2.01	0.030852	Unknown	
BB_A41	hypothetical protein	2.04	0.001775	Unknown	3.26
BB_M19	hypothetical protein	2.05	0.000541	Unknown	
BB_0722	hypothetical protein	2.07	0.026144	Unknown	
BB_0539	hypothetical protein	2.08	6.80E-05	Unknown	
BB_P19	hypothetical protein	2.08	0.000379	Unknown	
BB_L19	hypothetical protein	2.12	0.000527	Unknown	
BB_0165	hypothetical protein	2.16	2.20E-05	Unknown	2.15
BB_B26	hypothetical protein	2.17	0.036016	Unknown	
BB_J19	hypothetical protein	2.23	0.000301	Unknown	2.86
BB_G09	hypothetical protein	2.29	0.002647	Unknown	
BB_0541	hypothetical protein	2.31	0.003154	Unknown	
BB_L22	hypothetical protein	2.34	0.001125	Unknown	2.67
BB_A23	hypothetical protein	2.37	0.029657	Unknown	13.35
BB_A13	hypothetical protein	2.4	0.009654	Unknown	3.21
BB_J28	hypothetical protein	2.44	9.00E-06	Unknown	
BB_Q29	hypothetical protein	2.47	0.00047	Unknown	2.73
BB_K40	hypothetical protein	2.54	3.00E-06	Unknown	
BB_0739	hypothetical protein	2.58	8.10E-05	Unknown	
BB_0428	hypothetical protein	2.6	4.10E-05	Unknown	
BB_0811	hypothetical protein	2.62	1.80E-05	Unknown	
BB_0510	hypothetical protein	2.65	0.000438	Unknown	
BB_0825	hypothetical protein	2.81	0.000234	Unknown	
BB_O29	hypothetical protein	2.81	4.90E-05	Unknown	2.76
BB_0432	hypothetical protein	2.83	0.005391	Unknown	
BB_A09	hypothetical protein	2.84	0.029267	Unknown	4.76

BB_M17	hypothetical protein	3.07	0.039143	Unknown	2.45
BB_A40	hypothetical protein	3.09	4.00E-06	Unknown	5.43
BB_0449	hypothetical protein	3.28	1.00E-06	Unknown	
BB_0208	hypothetical protein	3.43	0.029958	Unknown	
BB_M41	hypothetical protein	3.43	0.000187	Unknown	
BB_0206	methyltransferase	3.46	3.00E-06	Unknown	4.05
BB_G29	hypothetical protein	3.55	0.009908	Unknown	6.22
BB_B10	hypothetical protein	3.56	0.000932	Unknown	5.50
BB_G17	hypothetical protein	3.56	0.034712	Unknown	
BB_M30	hypothetical protein	3.56	1.40E-05	Unknown	2.20
BB_D12	hypothetical protein	3.62	0.000156	Unknown	6.66
BB_L04	hypothetical protein	3.62	0.001493	Unknown	11.28
BB_0563	hypothetical protein	3.72	1.00E-06	Unknown	
BB_R44	hypothetical protein	3.77	0.004174	Unknown	
BB_K35	hypothetical protein	3.83	0.000455	Unknown	
BB_O43	hypothetical protein	3.86	0.047234	Unknown	
BB_0525	hypothetical protein	3.87	3.00E-06	Unknown	3.26
BB_M03	hypothetical protein	3.94	0.027709	Unknown	9.45
BB_0207	hypothetical protein	4.04	0.002233	Unknown	2.22
BB_F26	hypothetical protein	4.23	0.00052	Unknown	7.07
BB_O30	hypothetical protein	4.23	0	Unknown	3.47
BB_K47	hypothetical protein	4.39	2.00E-06	Unknown	3.03
BB_A54	hypothetical protein	4.4	4.90E-05	Unknown	2.81
BB_J46	hypothetical protein	4.48	0.000427	Unknown	
BB_L12	hypothetical protein	4.48	0.009449	Unknown	
BB_0077	hypothetical protein	4.53	0.002565	Unknown	
BB_0324	hypothetical protein	4.61	2.50E-05	Unknown	
BB_0756	hypothetical protein	4.67	0.003285	Unknown	
BB_R31	hypothetical protein	4.85	7.00E-06	Unknown	5.34
BB_L41	hypothetical protein	4.97	0.003423	Unknown	
BB_M07	hypothetical protein	4.98	0.000398	Unknown	5.80
BB_R29	hypothetical protein	4.99	3.00E-05	Unknown	3.26
BB_J48	hypothetical protein	5	0	Unknown	2.19

BB_N02	hypothetical protein	5.08	0.00311	Unknown	3.21
BB_G19	hypothetical protein	5.11	4.00E-06	Unknown	2.13
BB_0547	hypothetical protein	5.13	0.001003	Unknown	
BB_G31	hypothetical protein	5.35	7.00E-04	Unknown	3.80
BB_0048	hypothetical protein	5.46	0.000358	Unknown	
BB_J31	hypothetical protein	5.52	5.00E-06	Unknown	
BB_L18	hypothetical protein	5.69	0.042986	Unknown	
BB_0740	hypothetical protein	5.71	1.00E-06	Unknown	
BB_0523	hypothetical protein	5.89	3.30E-05	Unknown	
BB_J11	hypothetical protein	6.21	0.000128	Unknown	5.26
BB_0773	hypothetical protein	6.27	0.009977	Unknown	
BB_0762	hypothetical protein	6.31	1.20E-05	Unknown	
BB_Q78	hypothetical protein	6.33	0.015565	Unknown	
BB_B14	hypothetical protein	6.61	8.00E-06	Unknown	4.32
BB_F25	hypothetical protein	6.68	0	Unknown	2.34
BB_G18	hypothetical protein	6.88	2.10E-05	Unknown	2.00
BB_D16	hypothetical protein	7.15	0.021134	Unknown	10.72
BB_0631	hypothetical protein	7.9	0	Unknown	
BB_M39	hypothetical protein	8.08	3.00E-06	Unknown	
BB_K49	hypothetical protein	8.69	0	Unknown	4.15
BB_G12	hypothetical protein	9.62	1.00E-06	Unknown	
BB_O16	hypothetical protein	9.81	3.30E-05	Unknown	
BB_F22	hypothetical protein	10.91	0	Unknown	
BB_R22	hypothetical protein	11.61	3.00E-06	Unknown	
BB_N42	hypothetical protein	12.8	0	Unknown	
BB_Q18	hypothetical protein	13.78	5.50E-05	Unknown	
BB_O42	hypothetical protein	16.24	0.00089	Unknown	
BB_0049	hypothetical protein	19.08	0	Unknown	
BB_J38	hypothetical protein	23.36	0	Unknown	3.06
BB_0162	hypothetical protein	25.25	0	Unknown	4.26
BB_C08	hypothetical protein	28.87	0.004078	Unknown	
BB_S32	hypothetical protein	43.27	0	Unknown	

Table S4 Genes differentially regulated by the DksA-deficient strain in response to starvation condition.

ID	Symbol	Description	Relative expression RPMI / BSKII	FDR adjusted P-value	Category
BB_D21		Plasmid partitioning protein	2.12	7.90E-05	Cell Division
BB_Q06		membrane protein	4.34	0.007243	Cell Envelope
BB_J09	*ospD*	outer surface protein D	3.39	7.30E-05	Cell Envelope
BB_H41		membrane protein	3	0.027645	Cell Envelope
BB_0034		outer membrane protein P13	2.95	7.30E-05	Cell Envelope
BB_H06	*cspZ*	complement regulator-acquiring surface protein	2.84	0.018053	Cell Envelope
BB_B19	*ospC*	outer surface protein C	2.82	0.000143	Cell Envelope
BB_M27	*revA*	fibronectin binding protein	2.45	0.027645	Cell Envelope
BB_A24	*dbpA*	decorin-binding protein A	2.26	0.041396	Cell Envelope
BB_A36		lipoprotein	2.24	0.000304	Cell Envelope
BB_K01		lipoprotein	2.17	0.006755	Cell Envelope
BB_J36		lipoprotein	2.02	0.000143	Cell Envelope
BB_0716	*mreC*	rod shape-determining protein	-2.15	0.004589	Cell Envelope
BB_0234		integral membrane protein	-2.34	0.015231	Cell Envelope
BB_0574		integral membrane protein	-4.03	0.037345	Cell Envelope
BB_0147	*flaB*	flagellar filament core protein	2.55	0.000478	Chemotaxis and motility
BB_0570	*cheY*	chemotaxis protein CheY	2.05	0.005487	Chemotaxis and motility
BB_0276	*fliZ*	flagellar biosynthesis protein	-2.97	0.022595	Chemotaxis and motility
BB_0057	*gap*	glyceraldehyde 3-phosphate dehydrogenase	2.2	0.00343	Metabolism
BB_0630	*pfkB*	1-phosphofructokinase	2.13	0.003473	Metabolism
BB_0092	*atpD*	V-type ATPase subunit D	-3.25	7.80E-05	Metabolism
BB_F32		pseudogene	2.86	1.00E-05	Pseudogene
BB_Q57		pseudogene	2.05	0.032989	Pseudogene
BB_J15		pseudogene	-2.83	0.023975	Pseudogene
BB_0741	*groS*	chaperonin GroS	2.06	0.004867	Stress Response
BB_0697	*rimM*	16S rRNA-processing protein	-2.03	0.002797	Translation
BB_0487	*rpsQ*	30S ribosomal protein S17	-2.06	0.041396	Translation
BB_0469	*lspA*	lipoprotein signal peptidase	-2.19	0.002797	Translation

BB_B04	*chcB*	chitobiose transporter protein	2.24	0.00323	Transporter Proteins
BB_0448	*hpr*	phosphocarrier protein	-2.07	0.010755	Transporter Proteins
BB_0318		methylgalactoside ABC transporter ATP-binding protein	-2.15	0.003187	Transporter Proteins
BB_0640	*potC*	spermidine/putrescine ABC transporter permease	-2.45	0.014362	Transporter Proteins
BB_S32		hypothetical protein	3.56	0.003473	Unknown
BB_0541		hypothetical protein	2.27	0.023975	Unknown
BB_0049		hypothetical protein	2.2	0.009009	Unknown
BB_0162		hypothetical protein	2.07	0.002797	Unknown
BB_0752		hypothetical protein	-2.01	0.017376	Unknown
BB_0818		hypothetical protein	-2.06	0.028409	Unknown
BB_A54		hypothetical protein	-2.07	0.039268	Unknown
BB_0174		hypothetical protein	-2.14	0.041396	Unknown
BB_0790		hypothetical protein	-2.15	0.015231	Unknown
BB_0345		hypothetical protein	-2.37	0.000179	Unknown
BB_0139		hypothetical protein	-2.41	0.000312	Unknown
BB_G24		hypothetical protein	-2.6	0.006602	Unknown
BB_A13		hypothetical protein	-2.98	0.004566	Unknown
BB_0538		hypothetical protein	-3.25	0.000179	Unknown
BB_0851		hypothetical protein	-8.85	0.034811	Unknown

Table S5. Bacterial strains, plasmids, and oligonucleotides used in this study.

**Bacterial strain**	**Missing plasmids**	**Source**
B. burgdorferi B31-A3	lp-5,	(Elias et al., 2002)
B. burgdorferi B31-A3 Δ*dksA*	lp-5, 21, 25, 28-4	This study
B. burgdorferi B31-A3 Δrel_{Bbu}	lp-5, 21, 25, 28-4	This study
B. burgdorferi B31-A3 Δ*dksA*::pDksA	lp-5, 21, 25, 28-4	This study
B. burgdorferi 297	cp32- 6	(Hughes et al., 1992)
B. burgdorferi 297 Δ*dksA*	cp32- 6	This study
B. burgdorferi 297 Δ*dksA*::pDksA	lp28-1, 28-5, 38, cp32- 6	This study
Plasmid	Purpose	Source
pCm::Δ*dksA-aadA*	Deletion of *dksA* by homologous recombination in *B. burgdorferi* B31-A3	This study
pJSB636A	Deletion of *dksA* by homologous recombination in *B. burgdorferi* 297	This study
pCm::Δrel_{Bbu}-*aadA*	Deletion of rel_{Bbu} by homologous recombination in *B. burgdorferi* B31-A3	This study
pKFSS-1	*B. burgdorferi* plasmid vector, confers streptomycin resistance	(Frank et al., 2003)
pBSV2G	*B. burgdorferi* plasmid vector, confers gentamicin resistance	(Elias et al., 2003)
pKFSS-1::*dksA*-FLAG (pDksA)	Confer streptomycin resistance and expression of *dksA*	This study
pBSV2G::*dksA*-FLAG (pDksA)	Confer gentamicin resistance and expression of *dksA*	This study
Primers	Sequence (5′ to 3′)	Application
bb0332 RT F	ACCTCTGGATGGATTACAGAAAG	RT-qPCR
bb0332 RT R	CTACGTTGGGCATGCTAAGA	RT-qPCR
dksA RT F	AATCTTGAAGCGTTAGGTTTTGTT	RT-qPCR
dksA RT R	AAAGCATAAGGAATAGCTAAAAGTCTC	RT-qPCR
flaB RT F	AGCTCCCTCACCAGAGAAA	RT-qPCR
flaB RT R	GCATCACTTTCAGGGTCTCA	RT-qPCR

fliZ RT F	GTCGGAAGAAGAGCTGGA	RT-qPCR
fliZ RT R	CCTCAAGTTTAACATAATCATTCCC	RT-qPCR
rel$_{Bbu}$ RT F	GGAGCAAACAAAGAGCAAAG	RT-qPCR
rel$_{Bbu}$ RT R	CATTGTGCACTATTATTTCGTCTT	RT-qPCR
rplL RT F	TTCAGTAGGTTCGGCTGATTC	RT-qPCR
rplL RT R	CTCCAAGACCAAGTCCTGTAAT	RT-qPCR
rpoD RT F	TCTGATCAAGCTCGCACAAT	RT-qPCR
rpoD RT R	ATCCAAGCCTGTCTGAAAGC	RT-qPCR
ptsP RT F	CTTTCGGGCACTTAGGATGTAT	RT-qPCR
ptsP RT R	GCATAGGAACCATTACCCTTATCT	RT-qPCR
glpF RT F	AAGTCCCGAAATACCAGGAG	RT-qPCR
glpF RT R	TTCTTGCTGCTGTGTAAATACC	RT-qPCR
glpK RT F	TTATCTATTGATCAAGGTACTACTAGCTCG	RT-qPCR
glpK RT R	CCTGTATTTTTTTCCCATATAACCG	RT-qPCR
bba66 RT F	CTGCTTCTGGTGTGTTAGAGTT	RT-qPCR
bba66 RT R	GCTAGATGTTATTTCAAGTGCTAAAG	RT-qPCR
ospC RT F	CAGGGAAAGATGCGAATACATCTGC	RT-qPCR
ospC RT R	TAAGCTAAAGCTAACAATGATCC	RT-qPCR
dbpA RT F	GCTGCTCTTAAGGGCGTAAA	RT-qPCR
dbpA RT R	TAGCTCGCACTTTTGCTTCA	RT-qPCR
mlpI RT F	ACTGGTGCTGGAGAATTTGAT	RT-qPCR
mlpI RT R	CCTTAACCACCTCTTTGAAGGT	RT-qPCR
TB-bb0168-F1-5′	ACTGCT**GGATCC**AGTTATGGACTTCAAGGCTG	Mutagenesis
TB-bb0168-F1-3′	AACAGCTTTTGCAT**GAATTC**TCCCTATAAATTACA	Mutagenesis
TB-bb0168-F2-5′	GAAAAAAAGAACAAAA**ATCGAT**ACATCTTAAGTTAGTTA	Mutagenesis
TB-bb0168-F2-3′	ACTGCT**CTCGAG**AACCAACATTTTAAAATGATTGG	Mutagenesis
aadA-5′-EcoR1	ACTGCT**GAATTC**TACCCGAGCTTCAAGGAA	Mutagenesis
aadA-3′-ClaI	ACTGCT**ATCGAT**TATTTGCCGACTACCTTG	Mutagenesis
AG-bb0168-F1-5′	CAGCGTAAATAAGCAAGGAGAATATACAATAGGAGC	Mutagenesis
AG-bb0168-F1-3′	**GGCGCGCC**GGCTTGTGAAAGAGAGATTGCTAGGGAG	Mutagenesis
AG-bb0168-F2-5′	**GGCGCGCC**TCTTCAATAAACTCATGCTCAGAAACAGC	Mutagenesis
AG-bb0168-F2-3′	**GCGCGC**GATGCTGCACTTATCAAGATAGATAACATTC	Mutagenesis

flgBp-aphI-T7t 5′	**<u>GGCGCGCC</u>**TAATACCCGAGCTTCAAGGAAG	Mutagenesis
flgBp-aphI-T7t 3′	**<u>GGCGCGCC</u>**AGATCCGGATATAGTTCCTCCTTTC	Mutagenesis
dksA KpnI F	ACTGTA**<u>GGTACC</u>**TACAATTCCTTCAGTTTCAATAGC TTC	pDksA plasmid construction
dksA FLG XhoI R	ACTGCT**<u>CTCGAG</u>**TTATTTATCTGTCGTCATCGTCTT TGT AGTCTCTTTTGTTCTTTTTTTCTTTTTTTGTCTG	pDksA plasmid construction
P1	GCGGGATCCGGATTCTTTTACAATTCC	Mutant Confirmation
P2	AAAGCATAAGGAATAGCTAAAAGTCTC	Mutant Confirmation
P3	ACTGCTGAATTCTACCCGAGCTTCAAG	Mutant Confirmation
P4	ACTGCTGAATTCTATTTGCCGACTACC	Mutant Confirmation
aacC1 F	GCGGCTAGCTAGGTAATACCCGAGCTT	Mutant Confirmation
aacC1 R	CGCCTGCAGTTAGGTGGCGGTACTTGGG	Mutant Confirmation
dksA flanking F	TTAAAAATGTAATTTATAGGGAGG	Mutant Confirmation
dksA flanking R	TCCTAAGTCCTCCCC	Mutant Confirmation
aadA F	ACTGCTGAATTCTACCCGAGCTTCAAG	Mutant Confirmation
aadA R	ACTGCTGAATTCTATTTGCCGACTACC	Mutant Confirmation
kan F	ATGAGCCATATTCAACGGG	Mutant Confirmation
kan R	TTAGAAAAACTCATCGAGCATCAAATG	Mutant Confirmation
rel_{Bbu} flanking F	GAGCAAGGGCGCTGATATTGGGG	Mutant Confirmation
$re_{Bbu}l$ flanking R	GTGGGGCCTGTTGAGCTAAGC	Mutant Confirmation
